# Estimating Plant Species Richness With Sentinel and Landsat Data Across Ecosystems in China

**DOI:** 10.1002/ece3.72899

**Published:** 2026-03-16

**Authors:** Keman Wang, Yu Peng, Ying Ye, Yue Qi, Lizheng He

**Affiliations:** ^1^ College of Life and Environmental Sciences Minzu University of China Beijing China

**Keywords:** ecosystems, plant diversity, remote sensing, satellite, species diversity

## Abstract

The existing remote‐sensing models for estimating plant alpha diversity typically exhibit relatively low accuracy. This study endeavors to develop a high‐precision remote‐sensing model for plant species richness across multiple scales and diverse ecosystems in China. Sentinel‐2 and Landsat data were collected. A range of spectral vegetation indices, band indices, and their statistical metrics (mean, variance, coefficient of variation) were selected as predictor variables, with plant species richness serving as the response variable. Three modeling approaches, namely simple linear regression, multiple stepwise regression, and partial least squares regression (PLSR), were employed to evaluate the predictive capabilities of these variables across five vegetation types. The findings revealed that the PLSR model demonstrated the optimal performance, followed by multiple stepwise regression and simple linear regression. Among the spectral indices, EVI, DVI, PSRI, NDVI, PRI, GNDVI, and GMEVI were identified as powerful indicators for predicting plant species richness. Additionally, Sentinel‐2 data showed higher prediction accuracy compared to Landsat data, highlighting the crucial role of spectral and spatial resolution in the estimation of plant diversity.

## Graphical abstract

438 field plots were used to estimate plant diversity across ecosystems; 18 spectral indices were derived from Sentinel and Landsat data; EVI, DVI, PSRI, NDVI, PRI, GNDVI, and GMEVI were identified as powerful indicators for predicting plant alpha diversity.

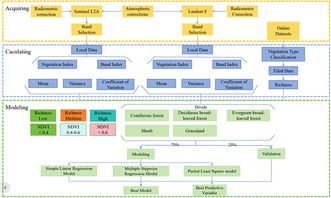

## 1 Introduction

As primary producers in ecosystems, plants provide habitats and ecological resources permeating the entire system, which is crucial for maintaining ecosystem balance, protecting overall biodiversity, preserving the human environment, and ensuring essential ecosystem services. As a vital component of biodiversity, plants have garnered extensive research attention (Bonar et al. 2011). Plant diversity encompasses the diversity of plant forms, levels, combinations, and their intricate interactions with other organisms and the environment, reflecting both the characteristics of plant communities and their relationships with the surrounding environment. However, amid escalating global warming, frequent disturbances, and intense human activities—major drivers of biodiversity loss (Imran et al. 2021)—plant diversity is under significant threat. Global biodiversity is declining at an unprecedented rate (Brondízio et al. 2021), with severe losses due to climate change regarded as an early warning of the sixth mass extinction (Barnosky et al. 2011; Ceballos et al. 2017; Dirzo et al. 2014; Mittermeier et al. 2011). Thus, assessing the current status, changes, and spatiotemporal patterns of biodiversity has become an urgent task, with growing demand for biodiversity assessment and monitoring research and application globally. Among these, species diversity is the most intuitive and core manifestation of biodiversity.

Species richness is widely used to quantify plant diversity (Thukral 2017). Traditional field measurements of such indices are laborious, costly (Bonar et al. 2011), and inconsistent in plot size, sampling units, and design across studies (Chiarucci 2007). They require expert species identification, with results varying by method (Bonar et al. 2011) and observer bias, introducing uncertainties. Critically, field surveys alone cannot capture landscape/regional species distribution changes. Remote sensing thus becomes a potential alternative for biodiversity estimation (Turner et al. 2003), offering consistent, efficient large‐scale plant diversity assessment (Nagendra et al. 2013) and long‐term global monitoring (Turner 2014). It overcomes fieldwork limitations (Lopes et al. 2017) with global, multiresolution data (Tang and Shao 2015), and its wide coverage and low cost enable broad application in plant diversity monitoring (Boschetti et al. 2007; Dalponte et al. 2008). It falls into direct/indirect (Turner et al. 2003) and active/passive frameworks (Liu et al. 2023). Direct methods use spaceborne sensors to map species directly (Palmer et al. 2002); indirect methods use remote sensing‐derived environmental parameters as biodiversity surrogates (Oindo and Skidmore 2010). Active sensors have their own energy; passive ones capture reflected sunlight via spectral images (Gholizadeh et al. 2018).

A variety of sensors, including multispectral (Gillespie 2005; Hauser et al. 2021; Madonsela et al. 2017; Taddeo et al. 2021), hyperspectral (Imran et al. 2021; Peng, Fan, et al. 2019; Peng, Wang, et al. 2019; Vaglio Laurin et al. 2014; Zhao et al. 2021), and active sensors such as Light Detection and Ranging (LiDAR) (Vaglio Laurin et al. 2016; Yi et al. 2020), have been utilized to predict species diversity under different vegetation types, climatic zones, and scales. The spectral and spatial resolution of sensors is critical factors for remote sensing images, as they can significantly influence the applicability and robustness of these images in characterizing and predicting plant diversity (Ganivet and Bloomberg 2019). Hyperspectral images, due to their numerous narrow bands, have the potential to predict species diversity with high accuracy (Gholizadeh et al. 2019; Ghosh et al. 2014; Nagendra et al. 2013). Active sensors, on the other hand, offer the advantage of providing more detailed vegetation structure data, which can enhance the prediction accuracy of plant diversity (Vaglio Laurin et al. 2016). However, the relatively high acquisition cost of hyperspectral and LiDAR data has limited their large‐scale application in diversity prediction (Mutowo and Murwira 2012; Rossi et al. 2021).

The Landsat (Land Remote Sensing Satellite) project, initiated by the United States Geological Survey (USGS) in collaboration with NASA, has been a cornerstone in Earth observation. Since the launch of the first Landsat satellite in 1972, which marked the beginning of a new era in space remote sensing, the development of Earth observation satellite remote sensing has accelerated rapidly. The orbit design of the Landsat series satellites features a near‐polar circular orbit that is synchronized with the sun. This design ensures that mid‐latitude regions of the northern hemisphere can obtain images with a medium solar altitude angle (ranging from 25° to 30°). Moreover, these satellites pass over the same location at the same local time and in the same direction, ensuring the basic consistency of remote sensing observation conditions, which is highly beneficial for data comparison. On February 11, 2013, the Landsat 8 satellite was successfully launched, inheriting the same orbital parameters as the Landsat 7 satellite. Equipped with a new generation of sensors, Landsat 8 has been improved in various aspects, including band resolution, radiation resolution, and data acquisition capabilities. The sensors on Landsat are capable of capturing visible, infrared, and thermal infrared spectral data, enabling it to provide multiband images that are invaluable for the identification and analysis of different surface features. Landsat data has long been an ideal choice for biodiversity research. For instance, Hantson et al. utilized Landsat data to study the vegetation conditions in the Southern California desert region and uncovered the climatic factors contributing to the decrease in vegetation coverage (Hantson et al. 2021). Dong et al. investigated the spatio‐temporal evolution of vegetation cover in the Hotan Oasis and proposed the impacts of human activities and climate change on vegetation cover changes in desert oases (Dong et al. 2019).

The Sentinel‐2 satellite, launched as part of the EU Copernicus program and the European Space Ag ency program, offers unique advantages for monitoring vegetation. Thanks to its high spatial resolution (10 m per pixel) and high temporal resolution (acquiring images once every 5 days), Sentinel‐2 can continuously and regularly monitor small vegetated areas over a large spatial scale (Defourny et al. 2019; Drusch et al. 2012). Additionally, the freely available nature of Sentinel‐2 data allows for the processing of large‐area data and serves as a cost‐effective complement to field investigations (Fauvel et al. 2020). As a result, Sentinel‐2 has emerged as the optimal data source when considering factors such as acquisition cost, spatial resolution, and spectral resolution (Mallinis et al. 2020; Yang and Shu 2022). Its potential for estimating plant diversity has been demonstrated in a wide range of ecosystems (Chrysafis et al. 2020; Fauvel et al. 2020; Liu et al. 2019; Torresani et al. 2021; Wang et al. 2022). Moreover, Sentinel‐2 is the only remote sensing image currently available that features three red‐edge bands, giving it a distinct advantage over other satellite images (Mutowo et al. 2018).

There are four main areas of research in plant diversity using remote sensing data: habitat mapping, species mapping, spectral diversity (heterogeneity), and functional diversity (Wang and Gamon 2019). Among these, spectral diversity (heterogeneity) has currently become the focal area for assessing plant diversity (Gholizadeh et al. 2018; Kacic and Kuenzer 2022), and it is also the central focus of this study. The most widely accepted theory in this field is the Spectral Variation Hypothesis (SVH) (Palmer et al. 2002). According to SVH, the spectral diversity (heterogeneity) of remote sensing images reflects the spatial changes in the environment, and these spatial changes are closely related to species richness (Palmer et al. 2002). In essence, SVH establishes a link between spectral diversity (heterogeneity) and environmental heterogeneity, providing a proxy for species diversity (Rocchini et al. 2010; Wang and Gamon 2019). Generally speaking, a higher degree of spectral heterogeneity is associated with a greater level of plant diversity (Tagliabue et al. 2020). A plethora of studies have employed proximal, aerial, and satellite remote sensing techniques to explore the correlations between spectral diversity and plant taxonomic, phylogenetic, and/or trait diversity (Dahlin 2016; Lucas and Carter 2008; Schweiger et al. 2017; Torresani et al. 2019). These studies have been conducted in a diverse range of ecosystems, including tropical savannas (Madonsela et al. 2021; Mapfumo et al. 2016), temperate grasslands (Peng, Fan, et al. 2019; Peng, Wang, et al. 2019), tropical evergreen broad‐leaved forests (Schwartz et al. 2015; Wallis et al. 2017), subtropical evergreen broad‐leaved forests (Liu et al. 2019), temperate deciduous broad‐leaved forests (Hoffmann et al. 2022; Kamoske et al. 2022), tropical semievergreen broad‐leaved forests (Hernández‐Stefanoni et al. 2011), subtropical evergreen sclerophyllous forests (Mohammadi and Shataee 2010), temperate deciduous coniferous forests (Zhou et al. 2022), coniferous forests (Michele et al. 2018), shrubs (Taddeo et al. 2021), wetlands (Tan et al. 2022), and mountain vegetation (Torresani et al. 2021). Overall, these studies suggest that multiple factors, such as scale, sensors, predictors, sampling methods, statistical models, seasons, and ecosystem types, can influence the extent to which spectral diversity can be translated into plant diversity (Schmidtlein and Fassnacht 2017; Wang et al. 2018). Additionally, both the complexity of plant communities and the vegetation coverage have been identified as important factors affecting the relationship between spectral diversity and plant diversity (Peng, Fan, et al. 2019; Peng, Wang, et al. 2019). The normalized difference vegetation index (NDVI) has been proven to be an excellent indicator of vegetation coverage (Calera et al. 2010; Carlson and Ripley 1997). The dominant role of vegetation cover as a driver of spectral diversity signals warrants further investigation in future applications of spectral diversity (Hauser et al. 2021).

The selection of appropriate spectral indicators is of critical importance for accurately quantifying plant species diversity (Rossi et al. 2021). To date, a plethora of indicators of spectral diversity (heterogeneity) have been proposed for estimating species diversity. These include spectral indices and various statistical indicators based on spectral indices (such as mean, standard deviation, and coefficient of variation) (Chitale et al. 2019; Torresani et al. 2020), heterogeneity/diversity indices specifically adapted for remote sensing (such as the Spectral Shannon and Simpson Index, Rao's *Q*) (Khare et al. 2021; Rocchini et al. 2017), texture features (Fundisi et al. 2020), convex hull volume (CHV) (Cornwell et al. 2006), spectral angle mapper (SAM) (Kruse et al. 1993), convex hull area (CHA) (Gholizadeh et al. 2018), spectral information divergence (Chein 2000), and distance from the spectral centroid (Rocchini 2007). However, these indicators of spectral heterogeneity exhibit significant variations in their spatial predictions of species diversity, and the best‐performing indicators may not be consistent across different application scenarios (Wang et al. 2018). Moreover, since the spectral diversity index is often jointly determined by plant species richness, cover, and biomass (Hooper et al. 2005; Tilman et al. 1996), some recent studies have suggested that relying solely on the spectral diversity index to predict species diversity has limitations and may even result in a negative correlation between the two (Taddeo et al. 2021). For example, Taddeo et al. (2021) found that in a single‐dominant wetland, the species richness was low, but it had a high greenness value (such as the green normalized vegetation index [GNDVI] and enhanced vegetation index [EVI]). Lin et al. (2021) also reported that “coastal mountains” had higher NDVI values but lower plant species diversity. Therefore, the primary objective of this study is to explore the use of general spectral diversity indices as predictors of plant species richness across the gradients of vegetation complexity in three vegetation types (forest, grassland, and shrub). By using two satellite imageries, Sentinel and Landsat, and three models (simple linear regression, PLSR, and random forest), the most suitable spectral diversity indices will be identified. The results of this study will be used to evaluate the predictive ability of the spectral indices of satellite data under different environmental conditions, thereby providing suitable indicators for the monitoring of plant diversity on a global scale.

## 2 Materials and Methods

### 2.1 Study Areas

**FIGURE 1:**
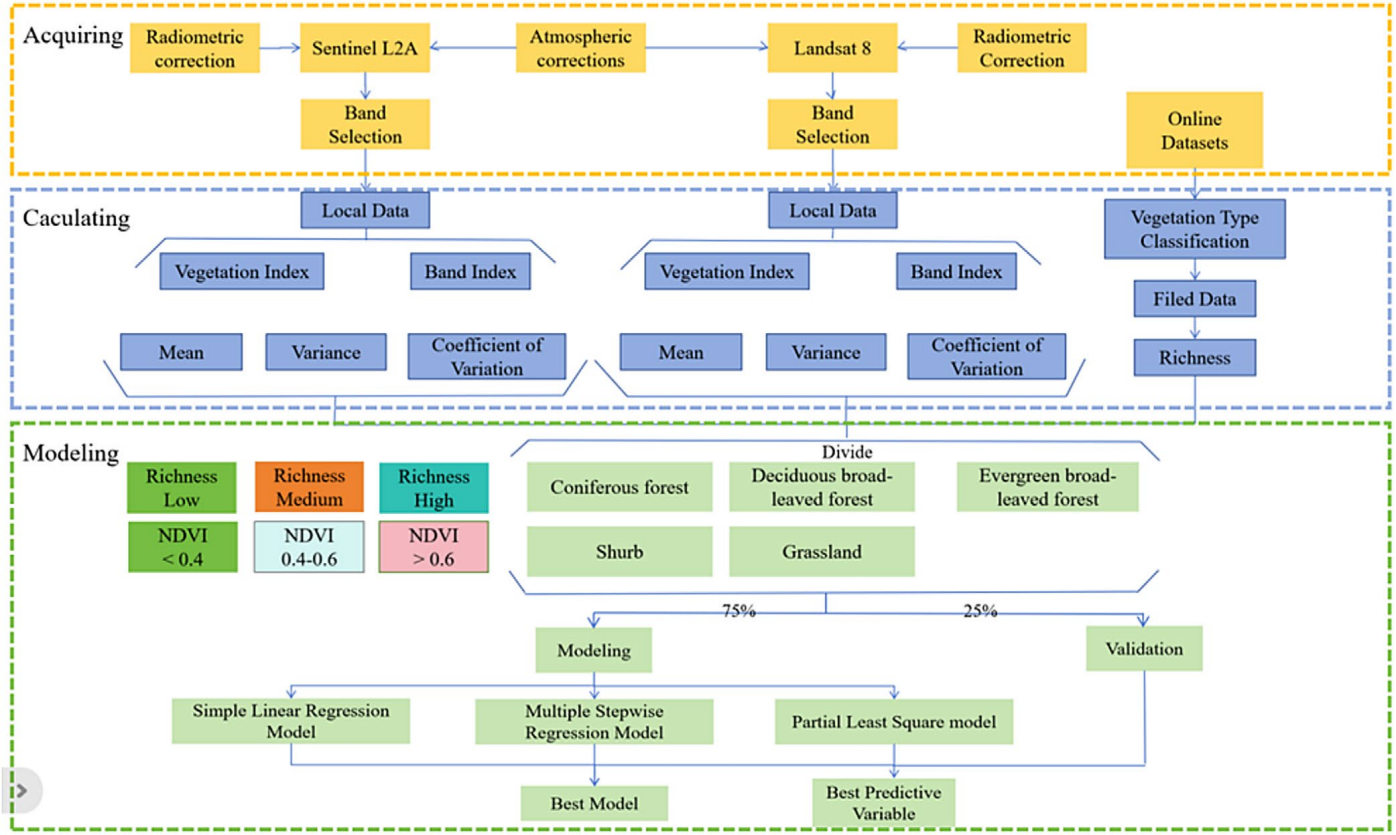
Flowchart illustrating the analysis procedure of this study.

**FIGURE 2:**
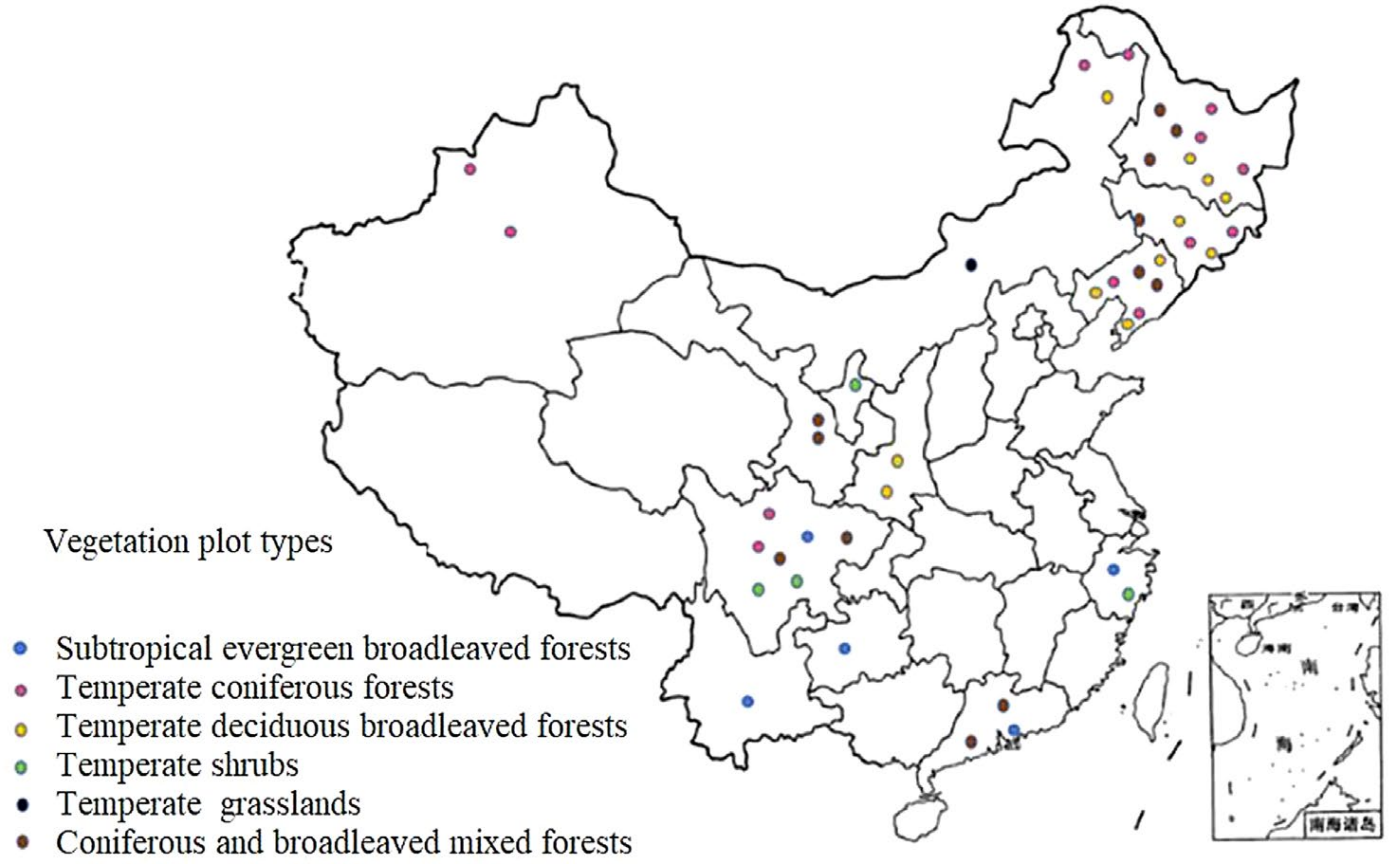
Distribution of vegetation plots across different ecosystems in China.

The flowchart illustrating the analysis procedure is shown in Figure 1. The sample plots utilized in this study are located in 14 provinces, namely Yunnan, Jilin, Guangdong, Gansu, Guangxi, Guizhou, Heilongjiang, Ningxia, Inner Mongolia, Sichuan, Shaanxi, Xinjiang, and Zhejiang (the repeated mention of Yunnan is likely an error and has been removed here). These plots encompass a diverse range of vegetation types, including larch forest, broad‐leaved forest, evergreen broad‐leaved forest, spruce forest, coniferous forest, tropical rainforest, shrubland, desert, xerophytic shrub area, grassland, and so on (Figure 2).

During the on‐site field investigation, the majority of the forest sample plots had dimensions of 30 m by 30 m, with a few measuring 20 m by 20 m. For each forest plot, typical tree species were carefully selected, and thorough field surveys along with data collection were carried out. The shrub sample plots had similar specifications. Most of the shrub plots were 30 m by 30 m in size, while a small number were 20 m by 20 m. Field investigations were conducted on these typical shrub plots. Regarding the grassland plots, they were all set at a size of 1 m by 1 m, and representative areas within the grasslands were chosen for the relevant investigations. The stratified sampling protocol finally consisted of 253 coniferous forest plots, 86 deciduous broad‐leaved forest plots, 19 evergreen broad‐leaved forest plots, 20 shrubland plots, and 60 grassland plots. The survey plots were affiliated with the Chinese Ecosystem Research Network (CERN; https://cern.ac.cn) and the scientific research project “National Protected Areas and Adjacent Areas” (Project No.: 2014FY110600), with relevant data and project information accessible via the National Ecosystem Science Data Center (NESDC; https://nesdc.org.cn). Although the plots exhibit an uneven spatial distribution across different ecosystems, this characteristic does not affect the accuracy of plant diversity estimation via satellite remote sensing. This is because, first, our study does not focus on the spatial associations among plots; second, all plots were strictly selected by investigators through field surveys to ensure that each plot belongs exclusively to a single vegetation type.

### 2.2 Sample Survey and Diversity Index Calculation

In this study, the indicator for calculating biodiversity is species richness (S). Species richness is defined as the total number of species existing within a community (Cui et al. 2014; Ganlin et al. 2006). The survey of species richness (S) was conducted between 2019 and 2020. The species richness values (S) for each study plot were also acquired from the CERN (https://cern.ac.cn) and the NESDC (2014FY110600) (https://nesdc.org.cn).

### 2.3 Optical Remote Sensing Image Data

Sentinel‐2 L2A images (atmospherically corrected, sourced from Copernicus Browser: https://browser.dataspace.copernicus.eu/) with < 10% cloud cover were acquired. The Sentinel‐2 constellation (Sentinel‐2A/2B) offers a 5‐day revisit period, 13 spectral bands (visible to short‐wave infrared), and spatial resolutions of 10/20/60 m; images were resampled to 10 m for subsequent processing. Landsat 8 OLI images (retrieved from EarthExplorer: https://earthexplorer.usgs.gov/) with < 10% cloud cover were also used. After preprocessing, correction, and enhancement, images from June to August (corresponding to the field quadrat measurement year) were selected, as this period aligns with the Northern Hemisphere vegetation growth season and improves plant diversity estimation accuracy via remote sensing (Arekhi et al. 2017). Examples of Landsat and Sentinel‐2 satellite remote sensing images for plots across different ecosystem types are provided in Figure S1.

### 2.4 Feature Extraction and Selection

In this particular study, existing multispectral images are considered. Common vegetation indices, along with their respective means, variances, and coefficients of variation, as well as the means, variances, and coefficients of variation of each band of different remote‐sensing images, are selected to predict plant richness. The prediction accuracies of Sentinel and Landsat satellites for different vegetation indices are explored (as presented in Table 1). The mean is a statistical measure that represents the average value of a dataset and serves to indicate the central tendency of the data. It is calculated by summing up all the values within the dataset and then dividing by the total number of data points. The mean can be applied to the pixel values of each band and is also used to compute the average value of each vegetation index.

**TABLE 1:** List of the calculation formula of spectral vegetation index.

Index	Symbol	Formula	References
Normalized difference vegetation index	NDVI	$\text{NDVI}=\frac{\mathrm{NIR}-R}{\mathrm{NIR}+R}$	Hoffmann et al. (2022)
Difference vegetation index	DVI	DVI=NIR−R	Richardson and Weigand (1977)
Enhanced vegetation index	EVI	$\mathrm{EVI}=\frac{2.5*\left(\mathrm{NIR}-R\right)}{\mathrm{NIR}+6R-7.5B+1}$	Huete et al. (1997)
Global environmental monitoring vegetation index	GEMVI	$\text{GEMVI}=\frac{\mathrm{NIR}-R}{\mathrm{NIR}+R+1}$	Rao et al. (2004)
Green normalized difference vegetation index	GNDVI	$\text{GNDVI}=\frac{\mathrm{NIR}-G}{\mathrm{NIR}+G}$	Daughtry et al. (1992)
Photochemical reflectance index	PRI	$\mathrm{PRI}=\frac{R_{\mathrm{ref}}-R_{531}}{R_{\mathrm{ref}}+R_{531}}$	Gamon et al. (1992)
Red edge chlorophyll index	RECI	$\text{RECI}=\frac{\mathrm{NIR}}{R}-1$	Rao et al. (2004)
Ratio vegetation index	RVI	$\mathrm{RVI}=\frac{R}{\mathrm{NIR}}$	Rozenstein et al. (2019)
Soil adjusted vegetation index	SAVI	$\text{SAVI}=\frac{\left(\mathrm{NIR}-R\right)}{\mathrm{NIR}+R+0.5}\left(1+0.5\right)$	Huete (1988)
Vertical growth vegetation index	VGCI	$\text{VGCI}=\frac{\mathrm{NIR}-R}{R}$	Thenkabail et al. (1994)
Normalized difference greenness index	NDGI	$\text{NDGI}=\frac{G-R}{G+R}$	Lyon et al. (1998)
Modified chlorophyll absorption in reflectance index	MCARI	$\text{MCARI}={\left(\left(\mathrm{NIR}-R\right)-0.2^{*}\left(\mathrm{NIR}-B\right)\right)}^{*}\left(\mathrm{NIR}/R\right)$	Daughtry et al. (1992)
Plant senescence reflectance index	PSRI	$\text{PSRI}=\frac{R-B}{\mathrm{NIR}}$	Merzlyak et al. (1999)
Infrared red edge chlorophyll index	IRECI	$\text{IRECI}=\frac{\mathrm{NIR}-R}{\mathrm{NIR}+G}*\frac{\mathrm{NIR}}{R}$	Frampton et al. (2013)
Ratio index	RI	$\mathrm{RI}=\frac{P\left(\mathrm{NIR}-G\right)}{\mathrm{NIR}+G}$	Huete (1988)
Mean	Mean	$\text{Mean}=\frac{\mathrm{Sum}\;\text{of}\;\mathrm{all}\;\text{values}}{n}$	Hunt and Rock (1989)
Variance	Variance	$V=\frac{\sum_{i=1}^{n}{\left(V_{i}-M\right)}^{2}}{n-1}$, vi = value, *M* = mean value	Pearson and Miller (1972)
Coefficient of variation	CV	$\mathrm{CV}=\frac{\text{Standard diviation}}{\text{Mean}}*100\%$	Rocchini et al. (2014)

Variance, on the other hand, is used to gauge the degree of dispersion of a dataset. In essence, it measures the deviation of data points from the mean. In the context of multispectral image analysis, variance can be employed to assess the extent of dispersion of pixel values across different bands and the variability of each vegetation index.

The coefficient of variation is defined as the ratio of the standard deviation to the mean. It is a valuable metric for measuring the relative degree of dispersion of data. One of its key advantages is that it can eliminate the impact of different units or varying means when comparing the variability of data. In this study, the coefficient of variation will be utilized to evaluate the dispersion degree of different vegetation indices and their corresponding multispectral band data. By calculating the coefficient of variation, we are able to compare the sensitivity of different vegetation indices and band data in predicting plant richness.

First and foremost, pixel values are extracted from the multispectral images captured by Sentinel and Landsat satellites, and subsequently, vegetation indices are calculated. For the pixel values of each band and each vegetation index, their means, variances, and coefficients of variation are determined. These statistical indicators are then used as predictors, with plant richness serving as the response variable, to develop regression models. The prediction accuracy of these models is evaluated, and the contributions of different vegetation indices and band data to the prediction of plant richness are compared. This study systematically assesses the prediction accuracies of Sentinel and Landsat satellites for different vegetation indicators, thereby providing a scientific foundation for the research on plant richness.

### 2.5 Statistical Analyses

We conducted in‐depth analyses by utilizing the simple linear regression model, the multiple stepwise regression model, and the PLSR (partial least squares regression) model.

Firstly, we carried out a fitting analysis of the simple linear regression model with a single factor. In this procedure, we initiated a Spearman correlation analysis on 75% of the measured data and the remote sensing data. The performance of the model was evaluated based on the significance *p*‐value and the determination coefficient (*r* ^2^) value. The optimal remote sensing indices were selected according to their statistically significant predictive ability (*p* < 0.05) and high coefficient of determination (*r* ^2^). The remaining 25% of the data was used for accuracy verification. It has been found that combining multiple heterogeneity indicators can improve the prediction accuracy of species diversity (Liu et al. 2023). As a result, we constructed a multivariate stepwise regression model and a PLSR model to predict plant diversity, using 75% of the data for model building and 25% of the data for accuracy validation.

Multiple linear regression is a technique used to study the linear relationship between one dependent and multiple independent variables. The multiple stepwise regression model, which is grounded in multiple linear regression, adds or removes independent variables from the regression equation through specific variable selection strategies (Jiange et al. 2011). Its aim is to identify the most explanatory combination of independent variables among a large number of possibilities and to build an optimal regression model. The stepwise method combines the characteristics of the forward and backward methods. At each step, it takes into account both the introduction of new variables and the elimination of existing variables in the equation that have become less significant. Initially, it introduces an independent variable that has the most significant impact on the dependent variable following the rules of the forward method. Through continuous iteration, the process stops when no more variables can be added or removed. This approach allows us to select the independent variables that have a substantial influence on the dependent variable from a large set of independent variables, thereby establishing a more accurate and reliable regression model. In both the linear regression model and the multiple linear regression model, the regression coefficient (*r*) was also calculated to evaluate the model's ability to estimate plant diversity.

A comprehensive correlation analysis was performed on the collected measured data and the corresponding remote sensing data. For variables with high correlation coefficients, they were removed from the variable pool to avoid the negative effects of multicollinearity on subsequent model construction. Then, for the remaining screened variables, the multiple stepwise regression analysis method was applied. This method, based on certain criteria (such as the Akaike Information Criterion, AIC), gradually incorporates independent variables with significant explanatory power into the regression model while excluding those that do not contribute significantly. In this way, a relatively optimized multiple linear regression model is obtained. During the implementation of the multiple stepwise regression, by continuously comparing the goodness of fit, significance levels, and other diagnostic indicators of different variable combinations, a regression model that can better explain the changes in the dependent variable is ultimately determined, providing a solid basis for further analysis and prediction. We used 70% of the total sample size for the multiple linear stepwise regression analysis to obtain the multiple linear regression model of the richness index, and 30% of the data was used for accuracy inversion.

PLSR is a linear nonparametric regression model (Verrelst et al. 2015) and a multiple regression algorithm developed to address the problems of multivariable collinearity and large data scale (Schweiger et al. 2017). The PLSR algorithm makes use of the data information within the independent variable and dependent variable systems to decompose and screen, extracting the comprehensive variable that has the strongest explanatory power for the dependent variable. It distinguishes between irrelevant and useful information in the system, eliminating the influence of multiple correlations among variables, and thus establishing an appropriate prediction model. PLSR can not only effectively solve the problems related to multilinear models but also handle issues that can be addressed by analysis methods such as principal component analysis, thereby providing more in‐depth and valuable information. Meanwhile, considering the relatively low prediction accuracy of the multiindex association method and the complex input parameters of the physical model, PLSR links the target variables with the predictor variables, which is easy to implement and can invert multiple traits with higher accuracy. It has been successfully applied in the research of traits in forest and grassland ecosystems (Schweiger et al. 2017; Zhao, Wang, et al. 2016), and is currently one of the high‐accuracy inversion modeling methods. The optimal inversion model for plant diversity was determined by comparing the accuracies of the simple linear, multiple stepwise regression, and PLSR models. By comprehensively analyzing the importance and significance of the inversion factors in each model, the crucial and robust remote sensing parameters for plant diversity inversion were also identified via Random Forest model. All the analyses were conducted using the packages of “vegan,” “lm,” “plsr,” “randomForest,” and “ggplot” in R software version 4.5.

## 3 Results

### 3.1 Accuracy of Simple Linear Regression Models

**FIGURE 3:**
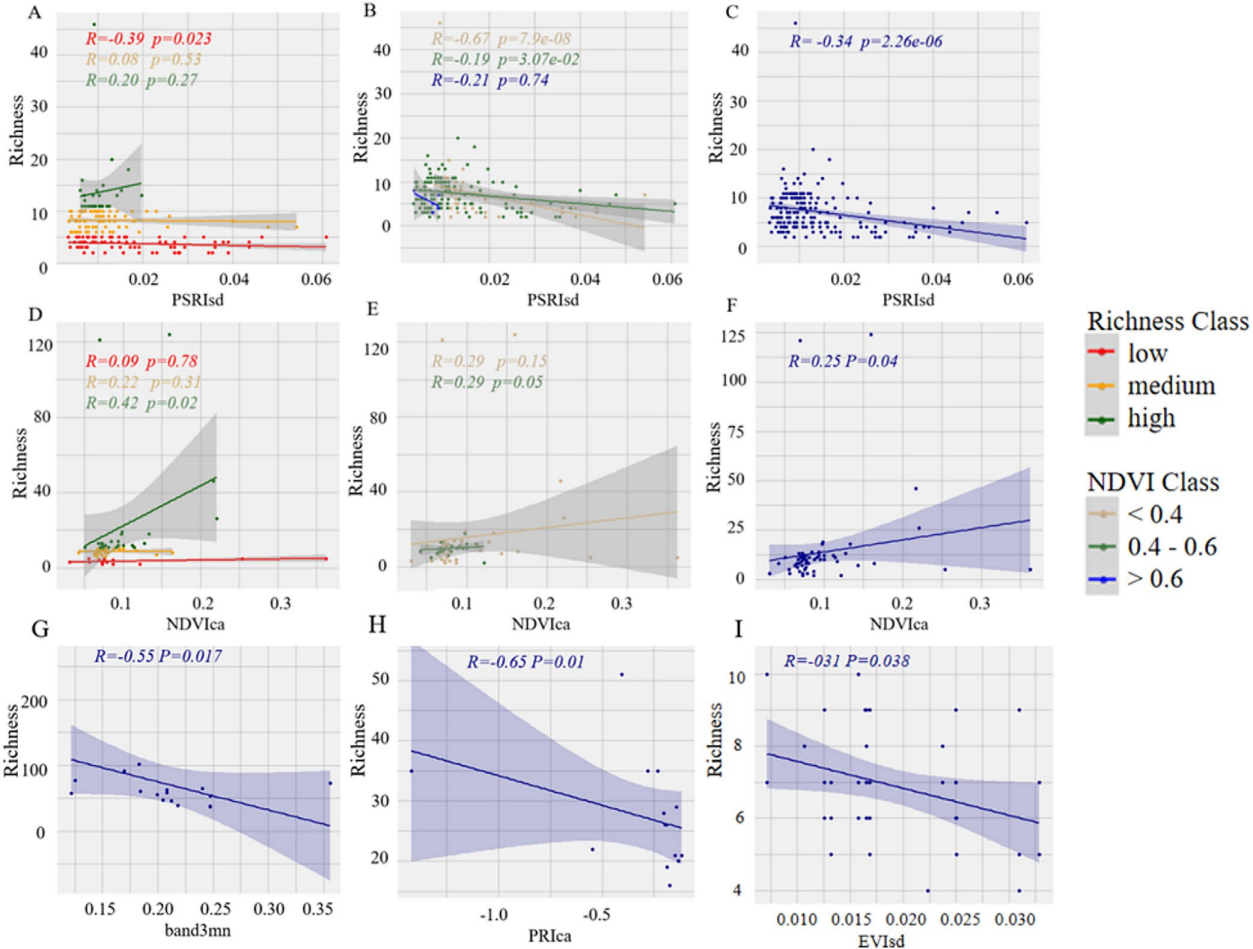
Simple linear regression models simulating inverse predictors of the richness index from Sentinel data for five vegetation types. (A: Coniferous forest, richness gradient; B: Coniferous forest, NDVI gradient; C: Entire coniferous forest; D: Deciduous broadleaved forest, richness gradient; E: Deciduous broadleaved forest, NDVI gradient; F: Entire deciduous broadleaved forest; G: Evergreen coniferous forest; H: Shrub vegetation; I: Grassland).

The findings indicate that the most suitable remote sensing index of Sentinel data for inverting the Richness index of northern coniferous forests is PSRIsd (as illustrated in Figure 3A–C). Additionally, PSRIsd demonstrates a very strong significance in inverting the Richness index at the low Richness gradient, where *R* = −0.39. When considering the NDVI gradients, PSRIsd shows a very strong significance in inverting the Richness index at both the low (*R* = −0.67) and medium (*R* = −0.19) NDVI gradients, yet it is ineffective at the high NDVI gradient (Figure 3B).

As for the deciduous broad‐leaved forest type, the NDVIca index exhibits the best inversion effect for the entire deciduous broad‐leaved forests (*r* = 0.25, Figure 3F). When analyzed according to the richness gradient, NDVIca has the highest inversion accuracy at the high richness gradient, with *R* = 0.42, but its inversion accuracy is relatively poor at the low and medium richness gradients (Figure 3D). In terms of the NDVI gradients, the inversion result of NDVIca for the medium NDVI gradient, with *R* = 0.29, is relatively significant (Figure 3E).

For the evergreen broad‐leaved forest type, the band3mn index shows the most significant inversion accuracy, with *R* = −0.55 (Figure 3G). Regarding the shrub type, the PRIca index has the highest inversion accuracy, with *R* = −0.65 (Figure 3H). And for the grassland type, the EVIsd index achieves the highest inversion accuracy, with *R* = −0.31 (Figure 3I).

Overall, the inversion accuracy was the highest in the region with the highest NDVI across all ecosystems. Notably, the pattern of inversion accuracy along the richness gradient was opposite between coniferous forests and deciduous broadleaved forests, indicating that inversion accuracy is strongly influenced by vegetation types.

**FIGURE 4:**
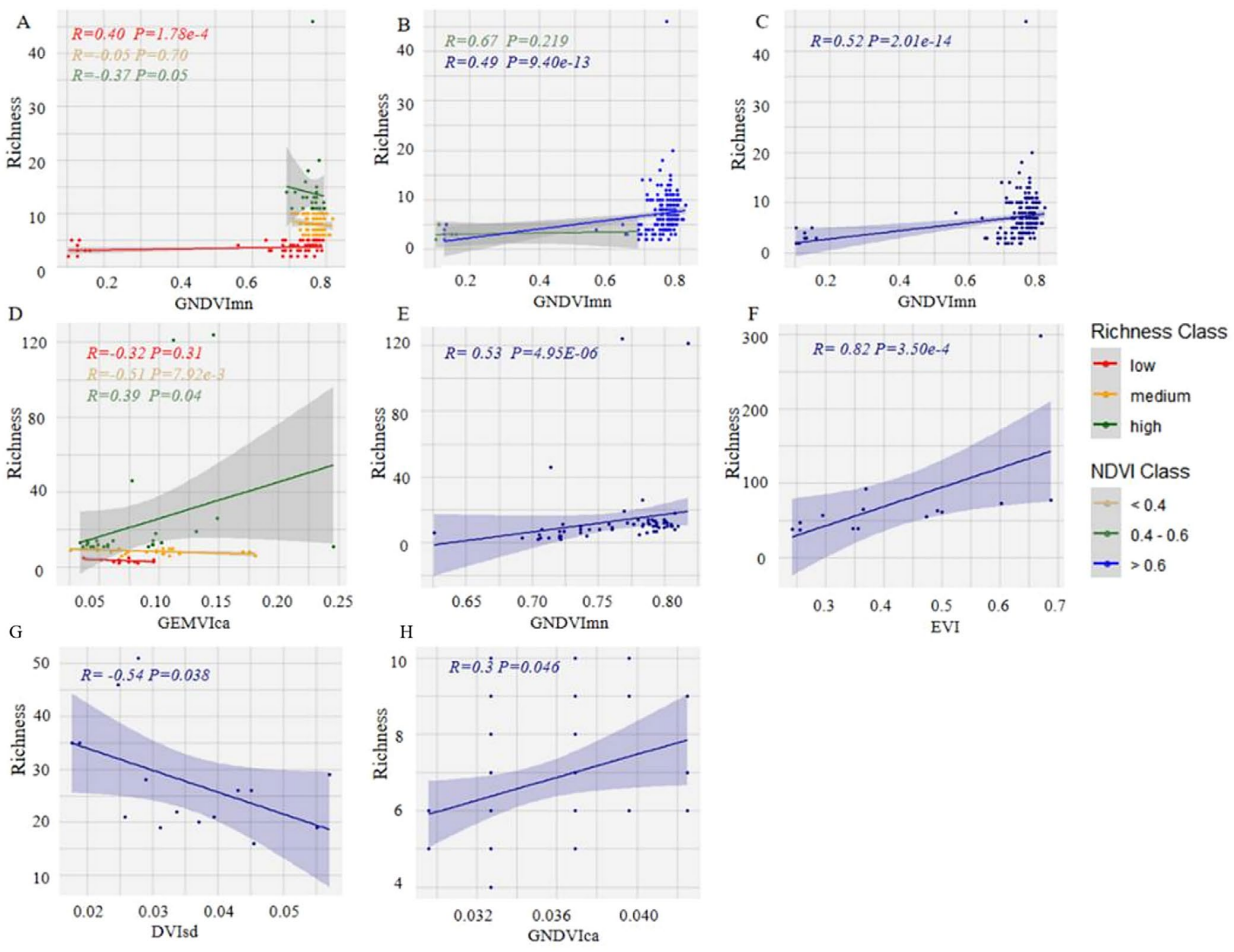
The simple linear regression model was applied to simulate the inverse predictors of the richness index based on Landsat data for five diverse vegetation types. (A corresponds to the Richness gradient in the coniferous forest; B represents the NDVI gradient in the coniferous forest; C refers to the coniferous forest in its entirety; D indicates the Richness gradient in the deciduous hardwood forest; E denotes the NDVI gradient within the deciduous hardwood forest; F–H is same as those in Figure 3).

The results suggest that the most suitable remote sensing index of Landsat data for inversely calculating the Richness index of northern coniferous forests is GNDVImn (as shown in Figure 4A–C). Additionally, GNDVImn shows a very strong significance in inversely calculating the Richness index at both the low Richness gradient (*R* = 0.40) and the high Richness gradient (*R* = −0.37), yet it fails to perform the inversion effectively at the medium Richness gradient (Figure 4A). At the NDVI gradient level, the GNDVImn index can effectively inverse the Richness index at the high NDVI gradient (*R* = 0.49) (Figure 4B).

GNDVImn also demonstrates a relatively high inversion accuracy for deciduous broad‐leaved forests (*R* = 0.53) (Figure 4E). When analyzed according to the Richness gradients, the GEMVIca index shows a very strong significance in inversely calculating the Richness index at both the medium Richness gradient (*R* = −0.51) and the high Richness gradient (*R* = 0.39) (Figure 4D). The optimal remote sensing parameter for inversely calculating the Richness index of evergreen broad‐leaved forests is EVI (*R* = 0.82) (Figure 4F). For the shrub type, the DVIsd index, with a correlation coefficient of *R* = −0.54, can effectively inverse its Richness index (Figure 4G). The inversion accuracy does not vary smoothly along the NDVI or species richness gradients, which reflects inconsistencies in the performance of spectral indices and across different ecosystem types derived from Sentinel‐2 data.

### 3.2 Accuracy of the Multiple Stepwise Regression Model

**FIGURE 5:**
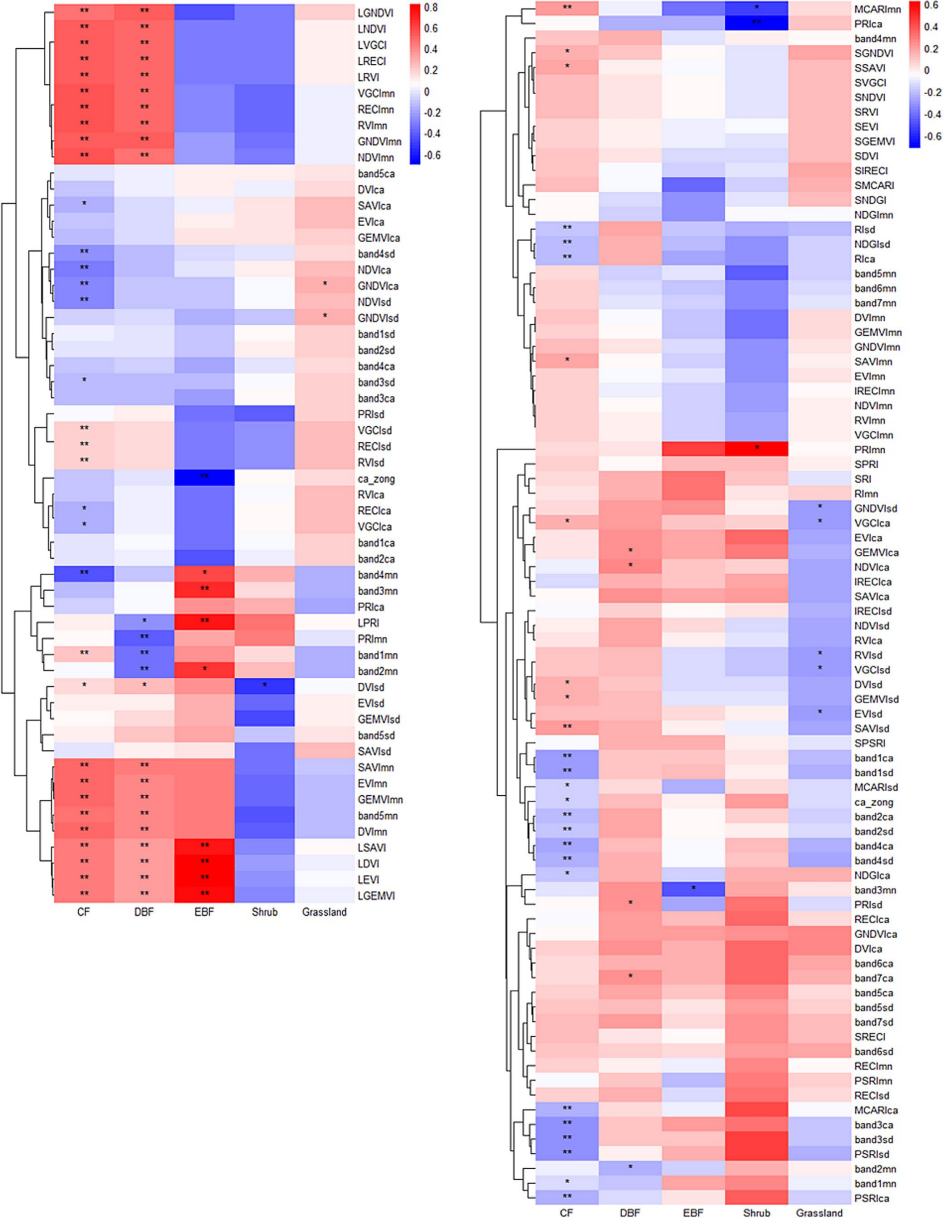
Illustrates the Spearman analysis results and their significance for each remote sensing index of the two types of remote sensing data. Specifically, the left panel shows the results for each remote sensing index of the Landsat data, while the right panel presents the results for each remote sensing index of the Sentinel data. In these visualizations, the color is used to represent the correlation, with darker colors indicating a stronger correlation. An asterisk (*) denotes a significance level of less than 0.05, and a double asterisk (**) signifies a significance level of less than 0.01.

The *R*‐squared values of the multiple stepwise regression model used for inverting plant diversity across various vegetation types are presented in Figure 5. When considering the inversion outcomes of Landsat data, the multiple stepwise regression model proves ineffective in inverting the diversity of coniferous forests, deciduous broad‐leaved forests, evergreen broad‐leaved forests, shrubs, and grasslands in their entirety, as indicated by the *R*‐squared values (*R* ^2^ < 0.2). Nevertheless, this model demonstrates the ability to account for the measured diversity values under the medium NDVI gradient condition of coniferous forests, with an *R*‐squared value of 0.52 (*R* ^2^ = 0.52).

### 3.3 Accuracy of Multiple Stepwise Regression Model

The R‐squared values of the multiple stepwise regression model for estimating plant diversity across different vegetation types are tabulated in Table 2. In terms of the inversion results derived from Landsat data, the multiple stepwise regression model fails to effectively estimate the diversity of coniferous forests, deciduous broad‐leaved forests, evergreen broad‐leaved forests, shrubs, and grasslands in their entirety, as evidenced by the *R* ^2^ values being less than 0.2 (*R* ^2^ < 0.2). However, it is capable of accounting for the measured diversity values within the coniferous forests under the medium NDVI gradient, with an *R* ^2^ value of 0.52 (*R* ^2^ = 0.52).

**TABLE 2:** The *R*‐squared values (*R* ^2^) of the multivariate stepwise regression models vary across different vegetation types and gradients.

Data type	Gratitude	Coniferous forest	Deciduous broad‐leaved forest	Evergreen broad‐leaved forest	Shrub	Grassland
Landsat	NDVI‐0.4	—	—	—	—	—
	NDVI‐0.4‐0.6	0.52	—	—	—	—
	NDVI‐0.6	0.15	—	—	—	—
	Richness‐low	0.08	0.28	—	—	—
	Richness‐medium	0.01	0.09	—	—	—
	Richness‐high	0.01	0.21	—	—	—
	All	0.17	0.04	0.09	0.13	0.11
Sentinel	NDVI‐0.4	0.45	0.21	—	—	—
	NDVI‐0.4‐0.6	0.29	0.24	—	—	—
	NDVI‐0.6	0.33	—	—	—	—
	Richness‐low	0.06	0.72	—	—	—
	Richness‐medium	0.06	0.21	—	—	—
	Richness‐high	0.01	0.12	—	—	—
	All	0.14	0.17	0.10	0.67	0.03

*Note:* In the provided figure, the symbol “—” indicates that no modeling was performed because there was insufficient data for the corresponding situation. This highlights the importance of having an adequate amount of data for successful model construction and also shows that certain scenarios may present challenges in creating reliable regression models due to data limitations or other factors related to the specific vegetation types and gradients under consideration.

When it comes to the inversion results based on Sentinel data (Table 2), the multiple stepwise regression model can satisfactorily account for the measured diversity values of shrubs, with an *R* ^2^ value of 0.67 (*R* ^2^ = 0.67). It is unable to effectively explain the measured diversity values of coniferous forests, deciduous broad‐leaved forests, evergreen coniferous forests, and grasslands as a whole, given that the *R* ^2^ values (*R* ^2^ < 0.2). Nevertheless, to some extent, it can explain the measured diversity values of coniferous forests under the low NDVI gradient, with an *R* ^2^ value of 0.45 (*R* ^2^ = 0.45). Additionally, it can effectively estimate the measured diversity values of deciduous broad‐leaved forests under the low Richness gradient, boasting an *R* ^2^ value of 0.72 (*R* ^2^ = 0.72).

### 3.4 Accuracy of PLSR Models

The *R*‐squared values of the PLSR model for estimating plant diversity across various vegetation types are presented in Table 3. In terms of the inversion outcomes based on Landsat data, the PLSR model is capable of effectively accounting for the measured diversity values of evergreen broad‐leaved forests, shrubs, and grasslands in their entirety. Conversely, it fails to adequately explain the measured diversity values of coniferous forests and deciduous broad‐leaved forests as a whole, with *R* ^2^ values less than 0.2 (*R* ^2^ < 0.2). However, it can offer a satisfactory explanation under the low Richness gradient, while its predictive performance remains moderate under other gradients (Table 3).

**TABLE 3:** The *R*‐squared values of the partial least square regression (PLSR) models vary significantly across different vegetation types and gradients.

Data type	Gratitude	Coniferous forest	Deciduous broad‐leaved forest	Evergreen broad‐leaved forest	Shrub	Grassland
Landsat	NDVI‐0.4	—	—	—	—	—
	NDVI‐0.4‐0.6	—	—	—	—	—
	NDVI‐0.6	0.26	—	—	—	—
	Richness‐low	0.14	0.63	—	—	—
	Richness‐medium	0.02	0.39	—	—	—
	Richness‐high	0.08	0.26	—	—	—
	All	0.14	0.11	0.99	0.98	0.54
Sentinel	NDVI‐0.4	0.86	0.87	—	—	—
	NDVI‐0.4‐0.6	0.48	0.83	—	—	—
	NDVI‐0.6	—	—	—	—	—
	Richness‐low	0.13	0.96	—	—	—
	Richness‐medium	0.01	0.59	—	—	—
	Richness‐high	0.07	0.70	—	—	—
	All	0.15	0.79	0.96	0.93	0.43

*Note:* In the given figure, the notation “—” indicates that modeling was not performed for the corresponding situation because of insufficient data. On the other hand, the symbol “×” implies that it was impossible to construct a valid PLSR model for that particular case.

When it comes to the inversion results of Sentinel data, the PLSR model can satisfactorily account for the measured diversity values of deciduous broad‐leaved forests, evergreen broad‐leaved forests, and shrubs as a whole. Its explanatory power for grasslands is somewhat limited, with an *R* ^2^ value of 0.43 (*R* ^2^ = 0.43), and its prediction performance for coniferous forests is rather poor, with an *R* ^2^ value of 0.15 (*R* ^2^ = 0.15). Additionally, when analyzed by gradients, the PLSR model demonstrates a good explanatory effect for deciduous broad‐leaved forests, with correlation coefficients (*R*) greater than 0.5 (*R* > 0.5). For coniferous forests, the inversion accuracy is relatively decent under the low and medium NDVI gradients (Table 3), yet the inversion effects across each gradient of the Richness classification are extremely poor, with *R* ^2^ values less than 0.2 (*R* ^2^ < 0.2).

The potential spectral indices identified via multiple stepwise regression and PLSR were also incorporated as key variables for predicting plant species richness in random forest models (Figures S2 and S3; Tables S1–S3), indicating that these indices exhibit nearly consistent performance in predicting plant species richness.

## 4 Discussion

This study aims to conduct a comparative analysis of the performance differences between Sentinel‐2 Level 2A data and Landsat data in inverting the measured values of plant diversity. By calculating and analyzing statistical metrics, including the mean, variance, and coefficient of variation of various vegetation indices and bands, we have thoroughly investigated their mechanisms of action and their significance within the inversion model. This research performs a comparative analysis based on the gradients of plant species richness and the normalized difference vegetation index (NDVI). Numerous vegetation indices have shown unique effectiveness in inverting different vegetation types. For example, the PSRIsd and GNDVImn indices have achieved remarkable results in the inversion of coniferous forests, which is consistent with previous research findings. Yao et al. (2017) indicated that the PSRI index has a highly significant predictive ability for the leaf area index (LAI) data of forest plots in western Fujian Province, providing strong evidence for the excellent performance of the PSRIsd index in inverting coniferous forests in this study. The GNDVImn index, due to its sensitive capture of the reflection differences between the near‐infrared and green light bands of coniferous forests, accurately reflects the vegetation physiology and structural characteristics of coniferous forests, further validating its effectiveness in coniferous forest inversion. As a classic index in the field of vegetation remote sensing, the NDVI has been widely used in monitoring vegetation growth status and coverage, as well as in climate response research. It plays an essential role in reflecting the dynamic changes of vegetation. The PRI index, by effectively representing the pigment content and photosynthetic efficiency of vegetation leaves, demonstrates good application potential in inverting vegetation types such as shrubs. The EVI, as an enhanced version of the NDVI, fully considers the reflectance of the near‐infrared, red, and blue bands. For evergreen broad‐leaved forests with thick cuticles and abundant chlorophyll, it can accurately interpret the measured richness values, especially performing well in low‐vegetation‐coverage areas like grasslands (Haimei et al. 2019; Wang and Wang 2024).

However, the results of the single‐factor linear regression inversion show that Sentinel data is only effective in inverting shrub types, while it has obvious limitations in inverting other vegetation types. Similarly, although Landsat data shows some performance in matching the measured data of shrub types, its inversion accuracy is poor when classified by gradient, and its inversion of other vegetation types is also unsatisfactory. This clearly demonstrates that the application of a single remote sensing factor is limited in complex vegetation communities or highly diverse environments. Although the remote sensing factors selected in this study have a statistically significant correlation with the plant diversity index (*p* < 0.05), the generally low correlation coefficient (*R* < 0.4) fully reveals the substantial uncertainty of the single‐factor model in inverting plant diversity. This is highly consistent with the research conclusion of Madonsela et al. (2017), further confirming the inherent flaws of the single‐factor model in handling the combined effects of multiple factors and nonlinear relationships, leading to a significant decrease in inversion accuracy and reliability.

Compared with the single‐factor model, multifactor modeling inversion has certain advantages in improving the estimation accuracy of plant diversity. Current research indicates that integrating multiple heterogeneity indices is conducive to improving the prediction accuracy of species diversity (Taddeo et al. 2019). The accuracy of a single‐factor prediction model is limited by a single index, resulting in relatively suboptimal final results. Multiple stepwise regression simplifies the model and avoids overfitting by gradually introducing or eliminating independent variables and automatically screening out those independent variables that have a significant impact on the dependent variable based on a predefined significance level (Jiange et al. 2011). Although it can mitigate the multicollinearity issue to a certain extent, when complex multicollinearity relationships exist among independent variables, multiple stepwise regression may not be able to handle them effectively, still leading to problems such as inaccurate parameter estimation. Even so, multifactor models using remote sensing data with different spectral and spatial resolutions have demonstrated varying levels of accuracy. Specifically, Landsat data generally performs poorly in inverting the plant diversity of coniferous forests, deciduous broad‐leaved forests, evergreen broad‐leaved forests, shrubs, and grasslands. This is mainly due to its relatively low spectral resolution, which makes it difficult to precisely capture the subtle spectral characteristic differences among various vegetation types. As a result, it cannot effectively extract the key information closely related to plant diversity. The changes in spectral reflectance caused by physiological characteristics such as the leaf structure and pigment content of different vegetation types often exceed the resolution capability of Landsat data, thereby negatively affecting the inversion accuracy. Nevertheless, under the medium NDVI gradient of coniferous forests, the multiple stepwise regression model can relatively well explain the measured diversity values. This may be because the vegetation growth status of coniferous forests is relatively stable under this gradient, allowing the NDVI value to effectively reflect parameters closely related to plant diversity, such as the leaf area index and biomass. The multiple stepwise regression model of Sentinel data shows a favorable explanatory effect on the measured diversity values of shrubs. This is largely because of the relatively simple vegetation structure and distinct spectral characteristics of shrubs. The high spectral and spatial resolution of Sentinel data enables it to precisely capture the vegetation information of shrubs. As Zhou et al. (2022) pointed out, factors such as the size, shape, and arrangement of shrub leaves lead to unique reflectance characteristics in different bands. Sentinel data can accurately identify these characteristics, thus successfully establishing an effective link with plant diversity. Under the low NDVI gradient, coniferous forests may be in the early growth stage or experiencing environmental stress, and the relationship between their spectral characteristics and diversity is more pronounced. The multiple stepwise regression model can capture this unique relationship and provide an explanation. For deciduous broad‐leaved forests under the low Richness gradient, the relatively low species richness simplifies the community structure, and the spectral characteristics of the dominant species become more prominent, which is conducive to Sentinel data extracting information related to diversity. However, overall, the ability of the multiple stepwise regression model to interpret plant diversity still has much room for improvement, which may be closely related to the model's inability to fully capture the complex characteristics of plant diversity.

PLSR can perform regression modeling when there is severe multicollinearity among independent variables. In the final model, all original independent variables are included, and it is easier to distinguish system information from noise (Zhao, Zeng, et al. 2016). During the modeling process, PLSR analysis combines the features of principal component analysis, canonical correlation analysis, and linear regression analysis methods. Consequently, in the analysis results, in addition to providing a more reasonable regression model, it can also simultaneously achieve some research content similar to principal component analysis and canonical correlation analysis (Schweiger et al. 2017), providing more abundant and in‐depth information. At the same time, given the relatively low prediction accuracy of the multi‐index combination method and the relatively complex input parameters of the physical model, PLSR correlates the target variable with the band spectral information. This approach is easy to implement and can invert multiple traits with relatively high accuracy. It has been successfully applied to trait research in forest and grassland ecosystems (Farifteh et al. 2007). In this study, only three common models were selected, while other statistical methods such as OLSR (Taddeo et al. 2019), GLM (Chitale et al. 2019), and SLR (Madonsela et al. 2017) were not considered and need to be supplemented in future research.

We further used the PLSR model to invert the measured diversity values of different vegetation types by integrating Landsat and Sentinel data (Supporting Information, Tables S4 and S5). The results show that the PLSR model performs relatively well in interpreting the measured diversity values of evergreen broad‐leaved forests, shrubs, and grasslands. This may be because these vegetation types have relatively obvious and stable spectral characteristics in remote sensing images, making it easier for the model to capture and interpret the relationship between them and the measured diversity values. However, for coniferous forests and deciduous broad‐leaved forests, especially in the inversion results of Landsat data, the overall interpretability of the model is relatively weak. This phenomenon may stem from the high complexity of the spectral characteristics of coniferous forests and deciduous broad‐leaved forests or the insufficiently strong correlation between their diversity and the spectral characteristics of remote sensing images. It is important to note that although the PLSR model has limitations in the overall interpretation of coniferous forests and deciduous broad‐leaved forests, its interpretability improves under specific gradient conditions (such as the low Richness gradient or the low and medium NDVI gradients). This could be because the diversity or spectral characteristics of vegetation types show higher consistency under these specific gradients, enabling the model to more effectively capture and interpret relevant characteristics. However, for other gradients, especially coniferous forests under the Richness subgradients, the model's prediction performance remains poor, further highlighting the complexity of the relationship between the spectral characteristics and diversity of coniferous forests.

Regarding the inversion results of Sentinel data, the PLSR model also demonstrates excellent performance in interpreting the measured diversity values of deciduous broad‐leaved forests, evergreen broad‐leaved forests, and shrubs (Supporting Information, Tables S4 and S5). This is likely closely related to the prominent and stable spectral characteristics of these vegetation types in Sentinel data (Xin et al. 2024). These spectral reflectance features are closely linked to the physiological and ecological traits of vegetation (such as chlorophyll content and leaf structure), allowing the PLSR model to relatively accurately capture and interpret the internal connections between them and the measured diversity values. However, when it comes to inverting coniferous forest types, the model's prediction performance is rather poor (*R* ^2^ = 0.15). This is mainly due to the unique spectral characteristics of coniferous forests. Their physiological and ecological characteristics, including leaf structure and chlorophyll content, differ significantly from those of other vegetation types. Moreover, the specific environmental conditions in which coniferous forests grow, such as high altitudes and cold regions, also have a significant impact on their spectral features, further increasing the difficulty of model inversion. This finding is consistent with the inversion results of Landsat data, fully validating the challenges of coniferous forests.

Based on the above results, we believe that the differences in spectral and spatial resolution of images from different remote sensing data providers are crucial factors contributing to the varying inversion effects. Although Landsat data has a relatively long time series and wide coverage area, its spectral resolution is somewhat limited, making it unable to precisely capture the subtle differences in the internal structure and species composition of vegetation. Spectral variability can serve as an indicator of plant species richness within a plant community. However, spectral variability derived from landscape‐scale satellite images often contains mixed background signals. This is because a 30‐m spatial resolution is too coarse to distinguish individual grass, shrub, or tree plants—nevertheless, each pixel within the analytical unit (i.e., a sample plot) is treated as a single‐species pixel in the current analysis. Even though Sentinel satellite data offers a finer spatial resolution (less than 10 m), its relatively low spectral resolution may mask the variability in physiological traits across different plant species. Consequently, despite Sentinel data possessing superior spatial resolution (compared to 30‐m imagery) and moderate spectral resolution, challenges such as data noise and spectral mixing may still hinder accurate inversion when analyzing specific vegetation types, particularly coniferous forests.

This study has explicitly identified the following limitations, which may contribute to the relatively low accuracy of plant diversity estimation via satellite remote sensing. First, there is insufficient exploration of predictive variables. Environmental variables (such as climate, precipitation, radiation, soil conditions, etc.), image texture features, and vegetation physiological indicators are all important characteristics for predicting plant species richness. Scaling up from plot‐level field observations to pixel‐level remote sensing data may also introduce potential uncertainties during the transition process, even though our study sites were strictly selected from the CERN, where environmental factors are generally uniformly distributed across a large spatial scale. However, this study has not fully considered these vegetation‐related factors. Second, the research has only analyzed and discussed five vegetation types and has not covered all possible ecosystems, which, to some extent, limits the generality of the research findings. During the remote sensing data collection phase, radiometric calibration errors may occur, and the constraints of spatial resolution can also affect the results. Regarding model selection and simulation, the inherent limitations of linear models will impose certain restrictions on the research. Even though the variance inflation factor (VIF) has been used to process spectral features, the processed spectral indices may still be redundant. Spectral diversity indices are influenced not only by the vegetation heterogeneity within the image but also by the soil background (Gholizadeh et al. 2018). Although Sentinel‐2 satellite data, due to its relatively coarse resolution, has a smoothing effect that reduces soil interference, the impact of the soil background in sparsely vegetated plots was not considered in our study. On the other hand, while remote‐sensing‐based monitoring of functional diversity has been effectively applied in forests, grassland species are smaller in size compared to forest ecosystems. Sentinel data calculates the variation among communities represented by pixels rather than the variation among species, resulting in certain differences in estimation accuracy (Mallinis et al. 2020). Additionally, this study focuses primarily on a single time window (June–August) and thus fails to capture the year‐round dynamic changes in biodiversity from a long‐term perspective. In future research, it will be essential to incorporate seasonal variations across spring, summer, autumn, and winter. Owing to the exclusive selection of plots with available field survey data, the number of plots is unevenly distributed; concurrently, the spatial distribution of ecosystems themselves is also heterogeneous. Nevertheless, this uneven distribution of sample plots does not affect the accuracy of our plant richness estimation via satellite remote sensing, as our study does not aim to examine the spatial patterns of ecosystems. To assess the performance of different models across various ecosystems, we employed both regression coefficients and statistical significance metrics—an approach that enhances the reliability of comparative analyses between the two sets of satellite data.

How to further improve the prediction accuracy and universality of remote sensing models for plant diversity is a crucial direction for future research. Based on the above limitations and discussion results, we propose the following prospects for future research: First, more accessible influencing factor indicators, such as derivative indicators, should be incorporated. Second, spectral indices need to be optimized, and more diverse spectral indices and spectral information processing methods should be tested. Third, the adoption of more diversified statistical models and deep learning algorithms is highly recommended to enhance model robustness. Fourth, abiotic factors such as soil background and biotic factors including vegetation phenological characteristics must be comprehensively incorporated into the analytical framework, as these variables significantly interfere with remote sensing signal interpretation. Fifth, the spatial scope of sampling should be expanded, and additional ground truth data that match the spatial resolution of remote sensing imagery should be collected to improve the representativeness of the dataset. When the spatial resolution of the image is increased to the scale of grassland species individuals (centimeter‐level), the estimation accuracy of functional diversity may be improved. Moreover, the use of hyperspectral data will also enhance the inversion accuracy of functional traits and, consequently, improve the inversion accuracy of functional diversity.

## 5 Conclusion

We conducted independent investigations on two distinct data sources, aiming to elucidate the relationships between spectral diversity and species richness for each data source, and to discuss the satellite remote‐sensing inversion of plant diversity across various vegetation types. In conclusion, among the models evaluated, the simple linear model demonstrated the lowest prediction accuracy, the multiple stepwise regression model showed a moderate performance, and the PLSR model achieved relatively superior results. Concerning the spectral indices, the EVI, DVI, PSRI, NDVI, PRI, GNDVI, and GMEVI indices, along with their derived coefficients, all emerged as powerful predictors of plant species richness. When contrasting the two satellite data sources, Sentinel‐2 data outperformed Landsat data in terms of prediction accuracy, indicating that disparities in the spectral and spatial resolution of the images are key determinants influencing the estimation of plant diversity.

Despite the PLSR model having a higher inversion accuracy compared to the single‐factor linear regression model and the multiple stepwise regression model, its capacity to interpret complex vegetation types, such as coniferous and deciduous broad‐leaved forests, remains restricted. Hence, future research should concentrate on the following areas: First, incorporate hyperspectral remote‐sensing data to obtain more comprehensive and detailed vegetation spectral information, particularly for complex vegetation types like coniferous and deciduous broad‐leaved forests. Second, continuously refine the model algorithm to overcome the limitations of existing models in dealing with complex plant diversity relationships. Third, integrate multiscale data and information to develop a more versatile and accurate plant diversity inversion model. By doing so, we can elevate the application of remote‐sensing technology in plant diversity monitoring, providing a robust scientific basis for the protection, management, and sustainable development of ecosystems.

## Author Contributions

**Keman Wang:** formal analysis (equal), writing – original draft (equal). **Yu Peng:** conceptualization (equal), funding acquisition (equal), project administration (equal), supervision (equal), writing – review and editing (equal). **Ying Ye:** formal analysis (equal), visualization (equal). **Yue Qi:** formal analysis (equal). **Lizheng He:** formal analysis (equal).

## Funding

This work was supported by the National Natural Science Foundation of China (32271555).

## Conflicts of Interest

The authors declare no conflicts of interest.

## Supporting information


**Data S1:** ece372899‐sup‐0001‐DataS1.pdf.

## Data Availability

All the required data are uploaded as Supporting Information.
