# Supplementary material for: Estimating Plant Species Richness With Sentinel and Landsat Data Across Ecosystems in China
Source: Ecol Evol. 2026 Mar 16;16(3):e72899. doi: 10.1002/ece3.72899 (PMC13093663; doi:10.1002/ece3.72899)
Supplement: Supplementary file 1 — Data S1: ece372899‐sup‐0001‐DataS1.pdf. [file ECE3-16-e72899-s001.pdf]

Supplementary materials

FIGS1. Satellite images of representative plots across different ecosystem types

Ecosystem types	Landsat	SN2
Subtropical evergreen broadleaved forest	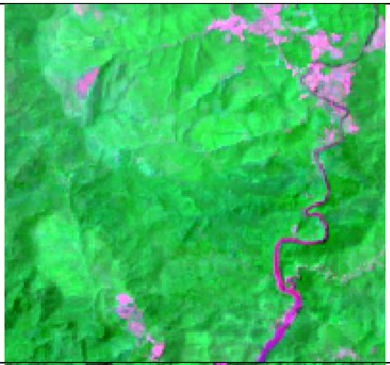	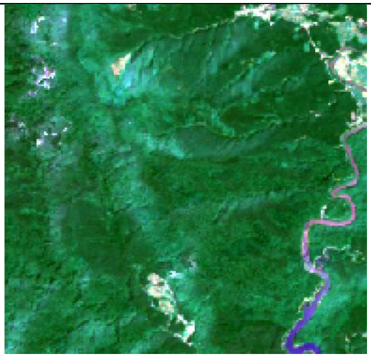
Temperate deciduous broadleaved forest	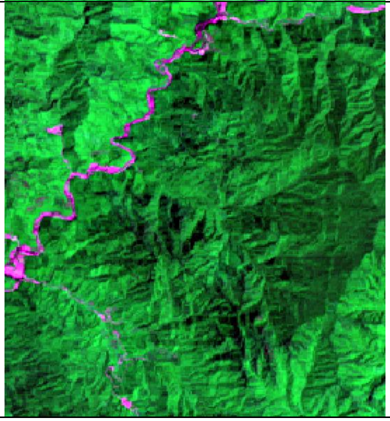	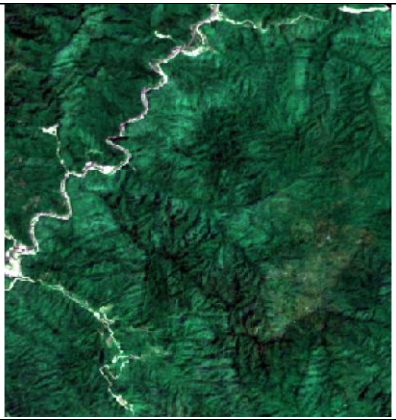

Temperate coniferous broadleaved- mixed forest	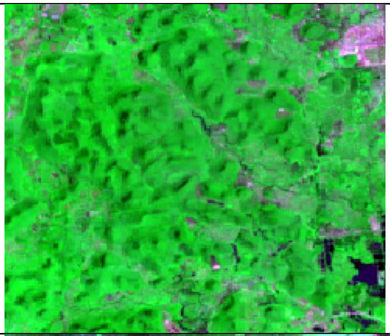	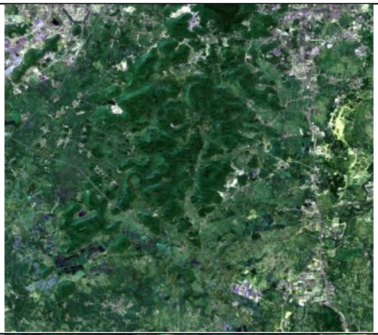
Temperate coniferous forest	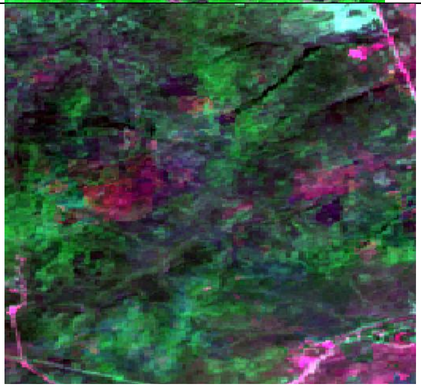	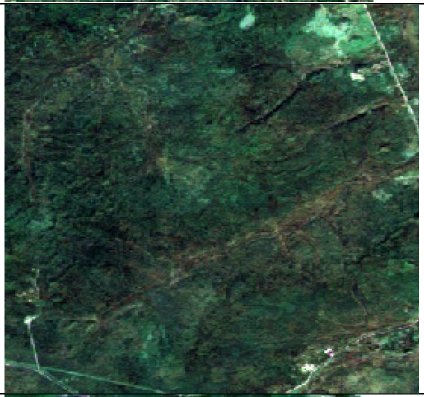
Shrub	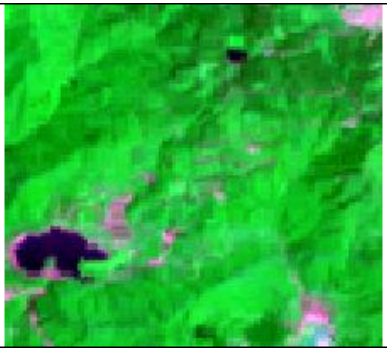	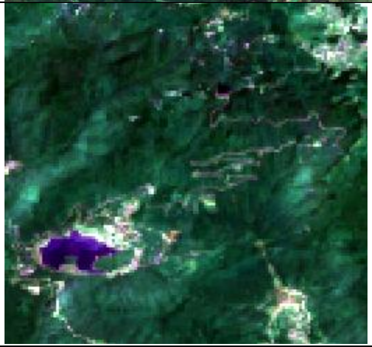

Grassland	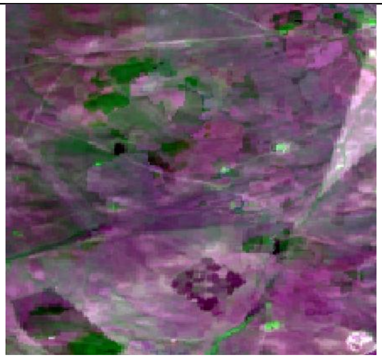	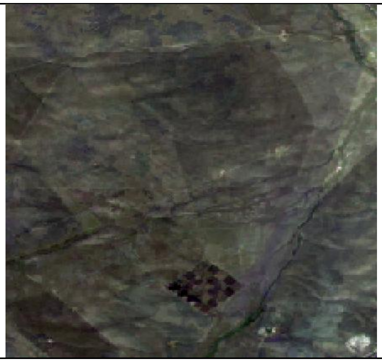

FIGS2. Random Forest Model in Six Different Vegetation Types Based on Sentinel-2 Data (the prefix "S" for some vegetation indices represents the index values from Sentinel-2 data)

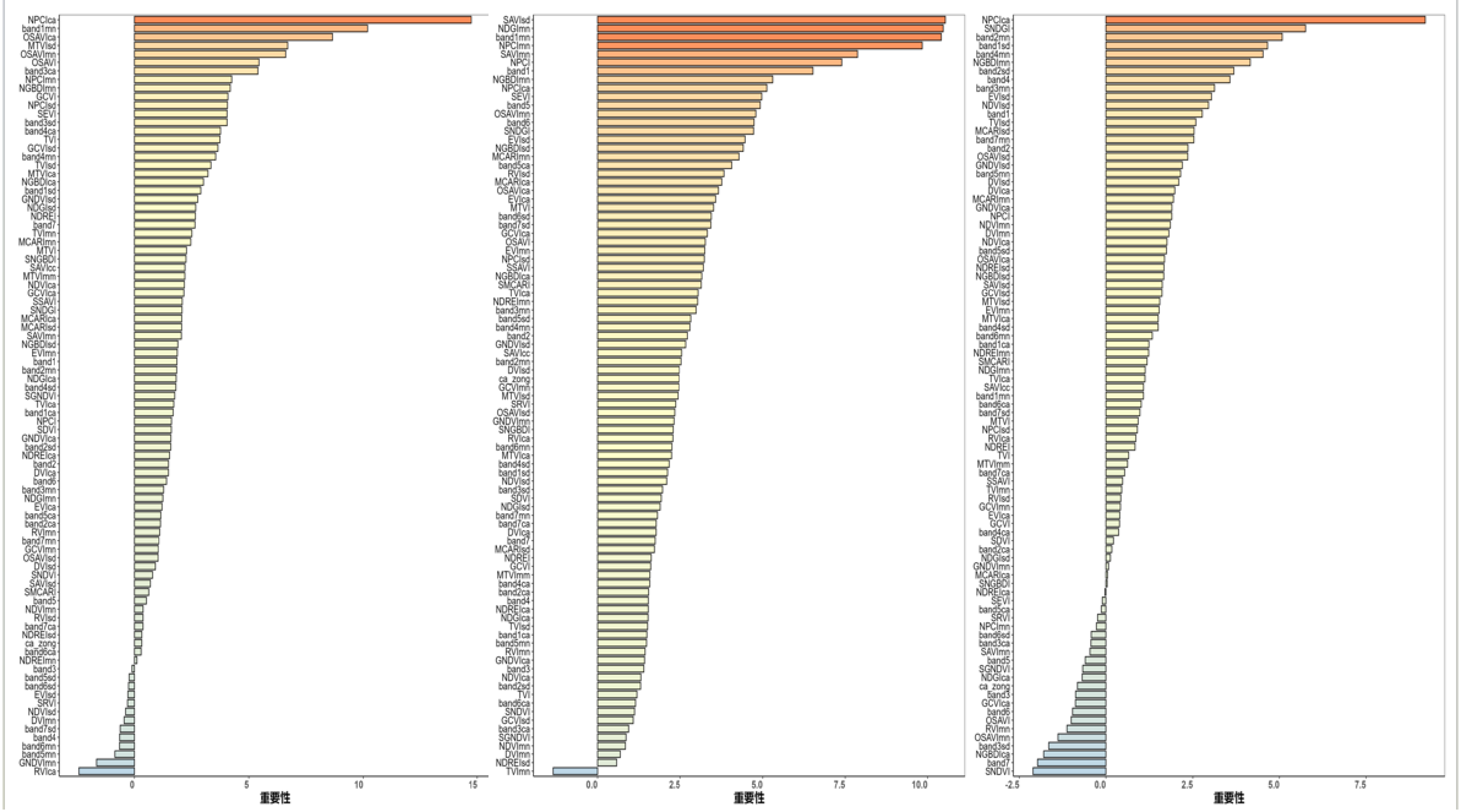

The performance of Random Forest models constructed for coniferous forest, deciduous broad-leaved forest, and coniferous-broad-leaved mixed forest in sequence.

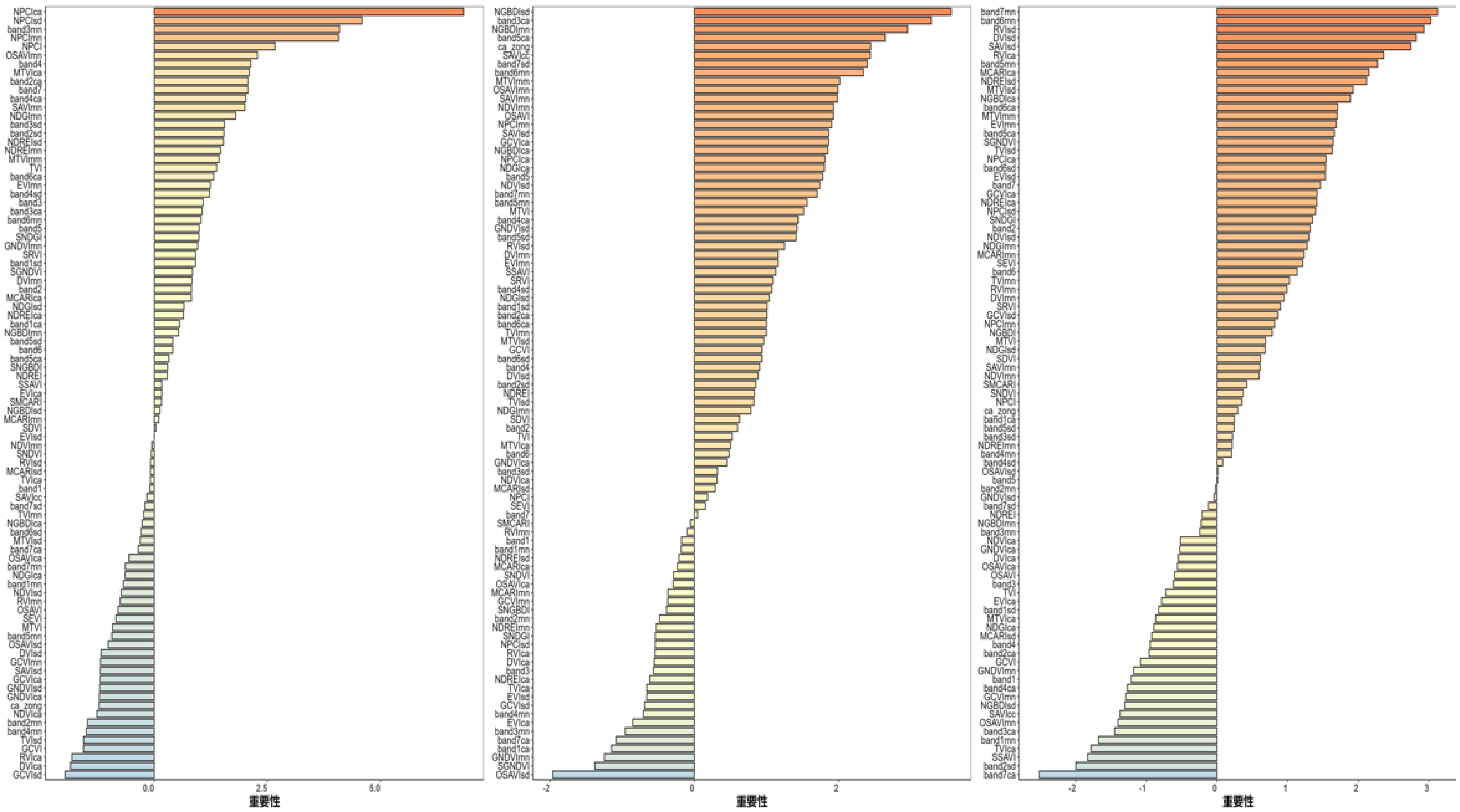

The performance of Random Forest models constructed for subtropical evergreen broad-leaved forest, grassland, and shrubland in sequence.

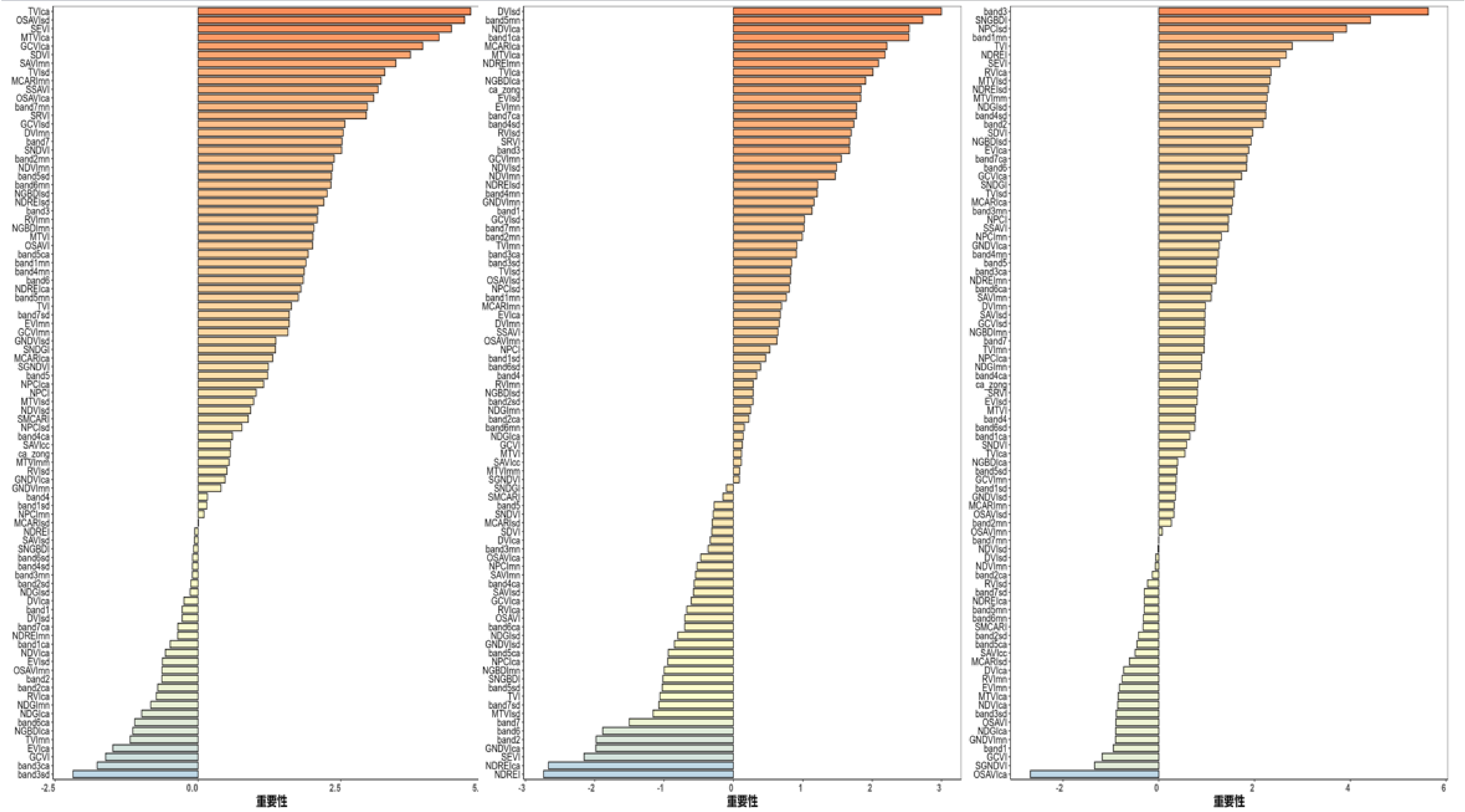

The performance of Random Forest models constructed for coniferous forests with low, medium, and high richness gradients in sequence.

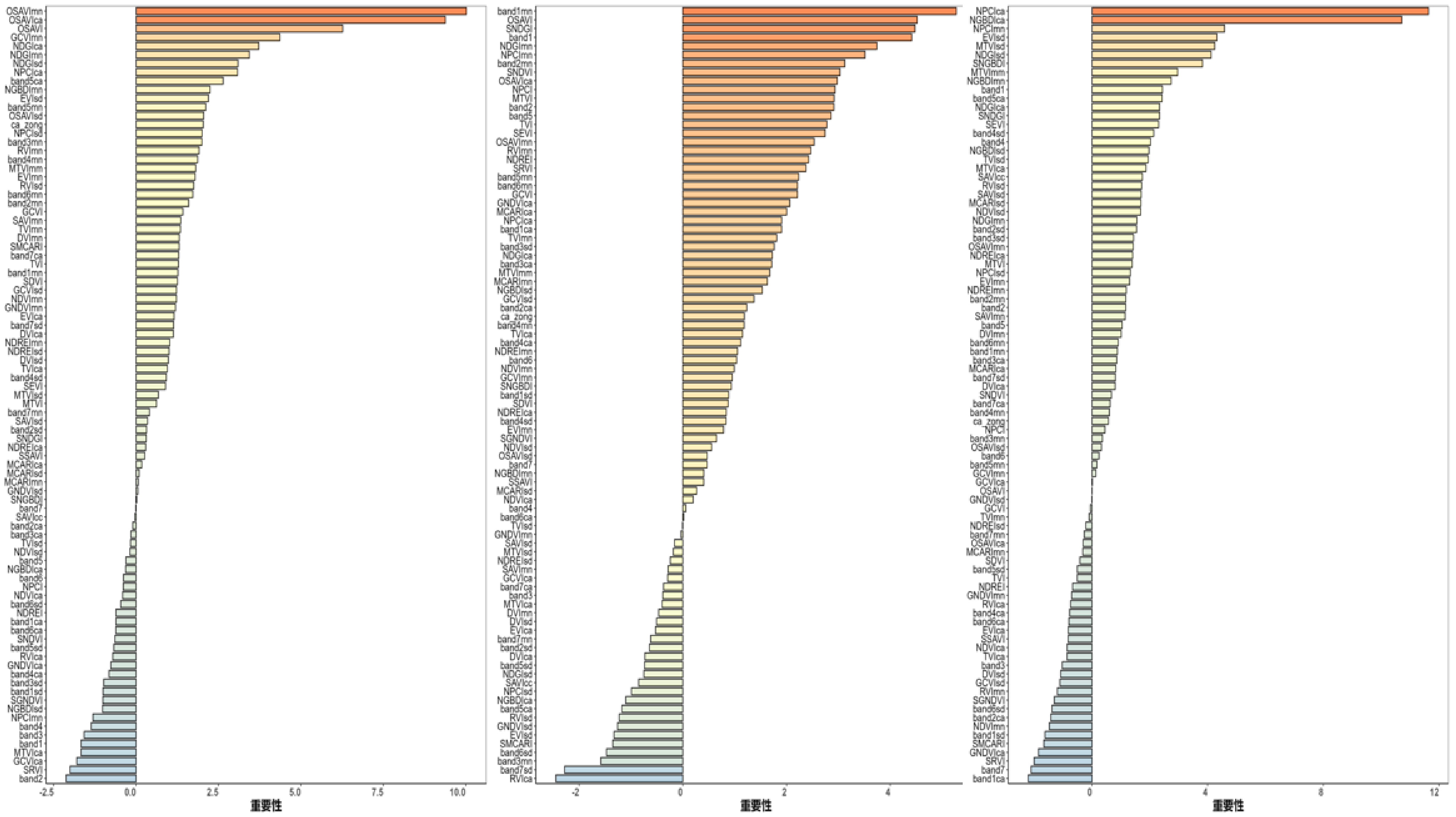

The performance of Random Forest models constructed for coniferous forests with low, medium, and high NDVI gradients in sequence.

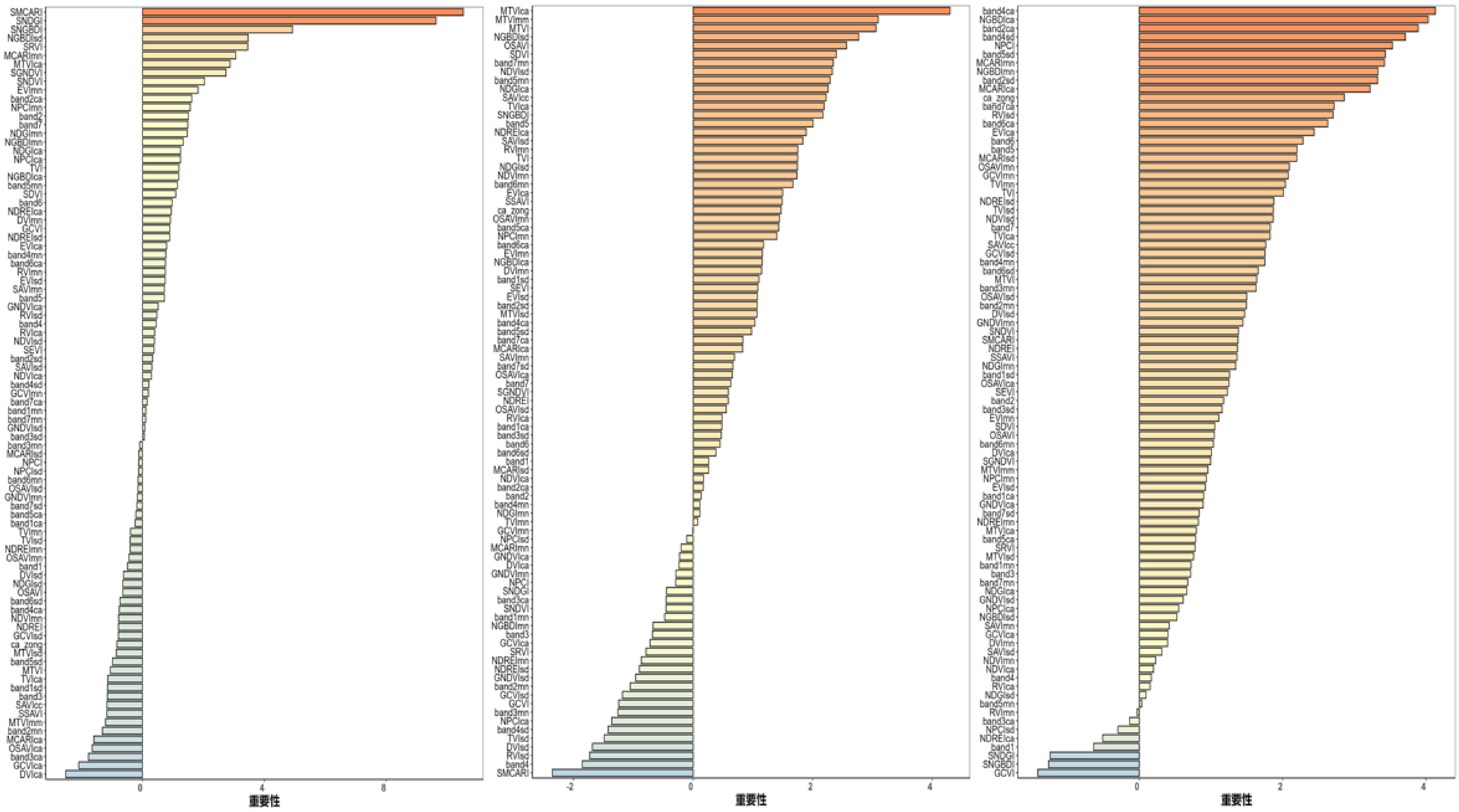

The performance of Random Forest models constructed for deciduous broad-leaved forests with low, medium, and high richness gradients in sequence.

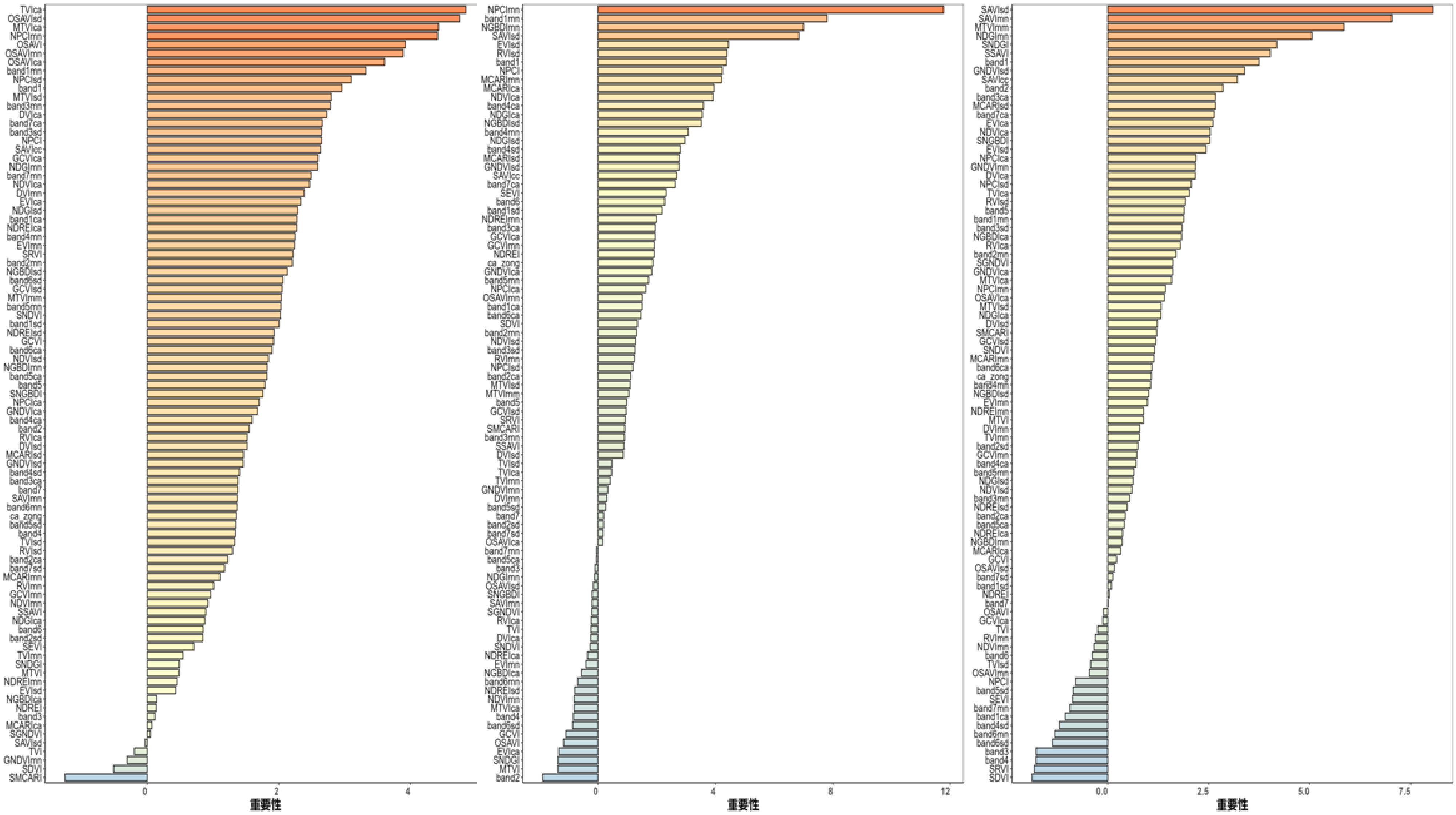

The performance of Random Forest models constructed for deciduous broad-leaved forests with low, medium, and high NDVI gradients in sequence.

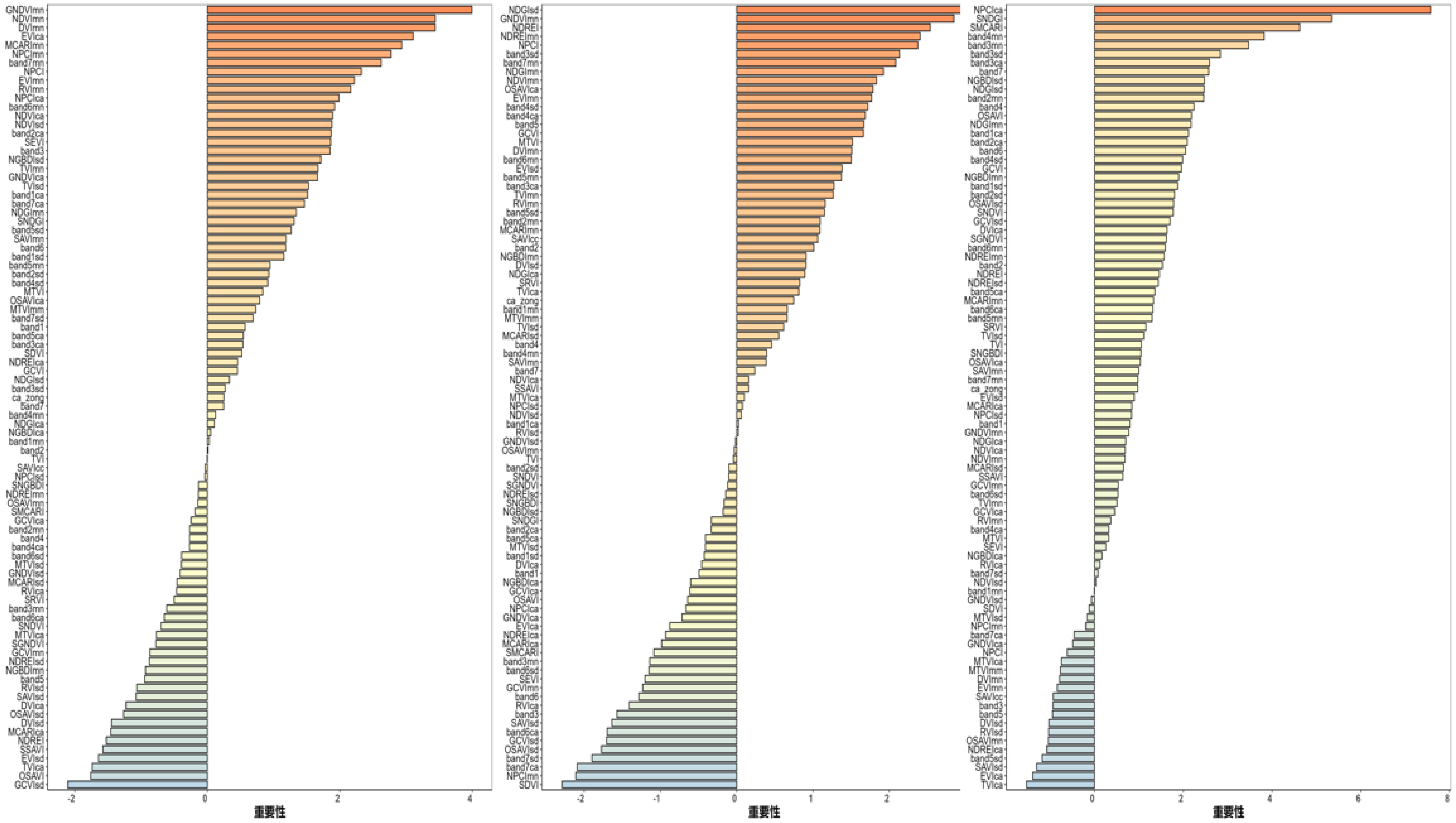

The performance of Random Forest models constructed for coniferous-broad-leaved mixed forests with low, medium, and high richness gradients in sequence.

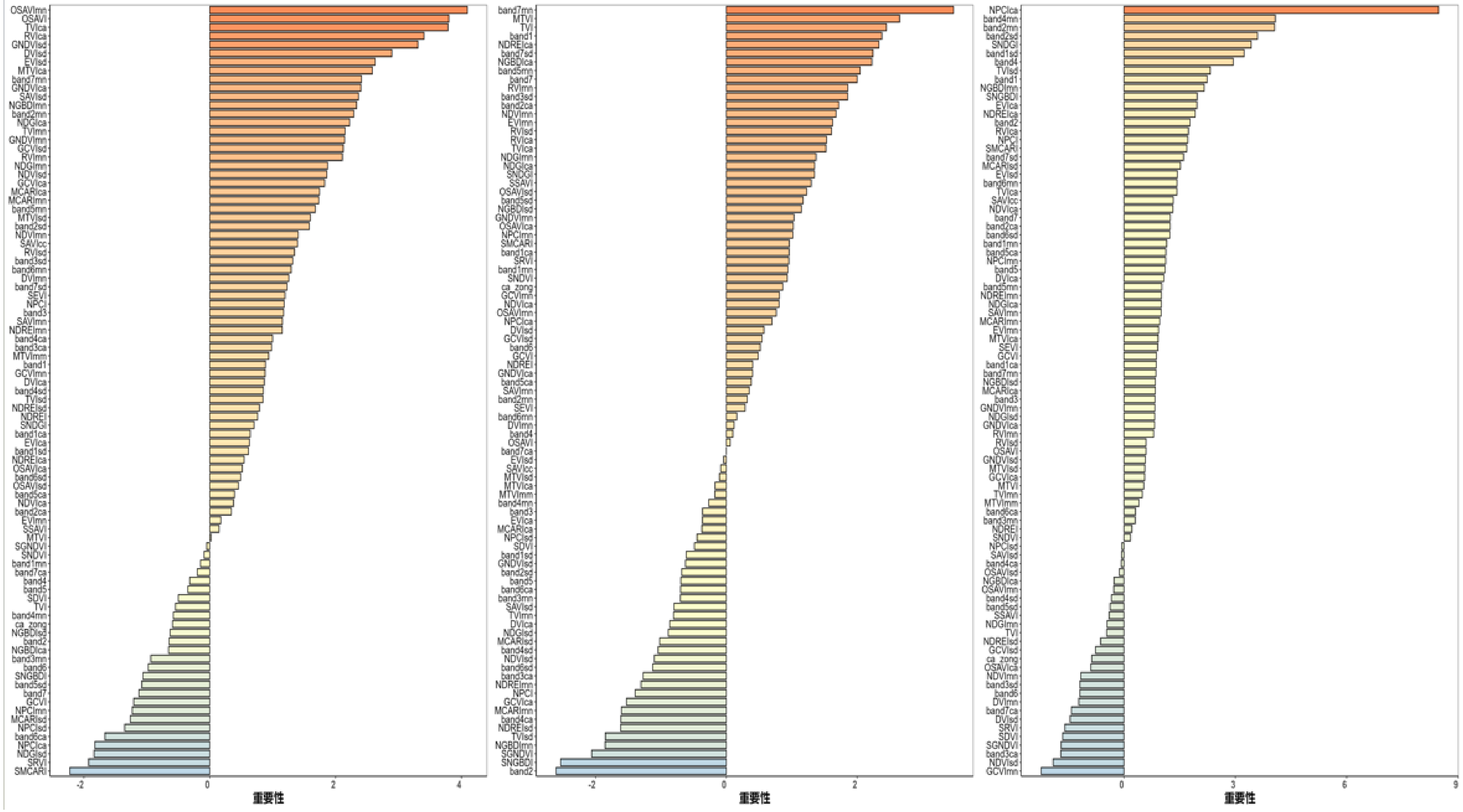

The performance of Random Forest models constructed for coniferous-broad-leaved mixed forests with low, medium, and high NDVI gradients in sequence.

FIG S3 Random Forest Models in Six Different Vegetation Types Based on Landsat Data (the prefix "L" for some vegetation indices represents the index values from Landsat data)

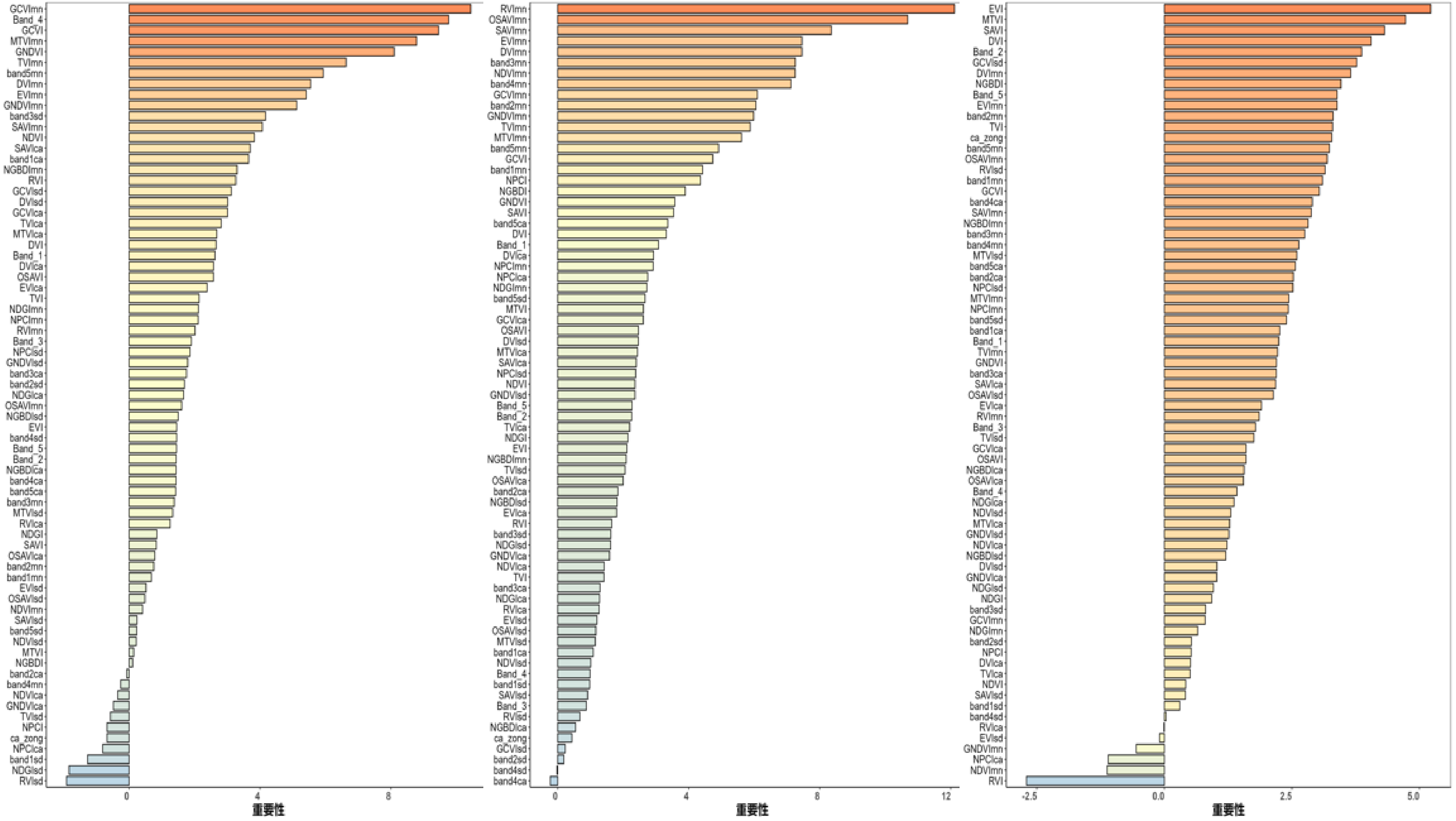

The performance of Random Forest models constructed for coniferous forest, deciduous broad-leaved forest, and coniferous-broad-leaved mixed forest in sequence.

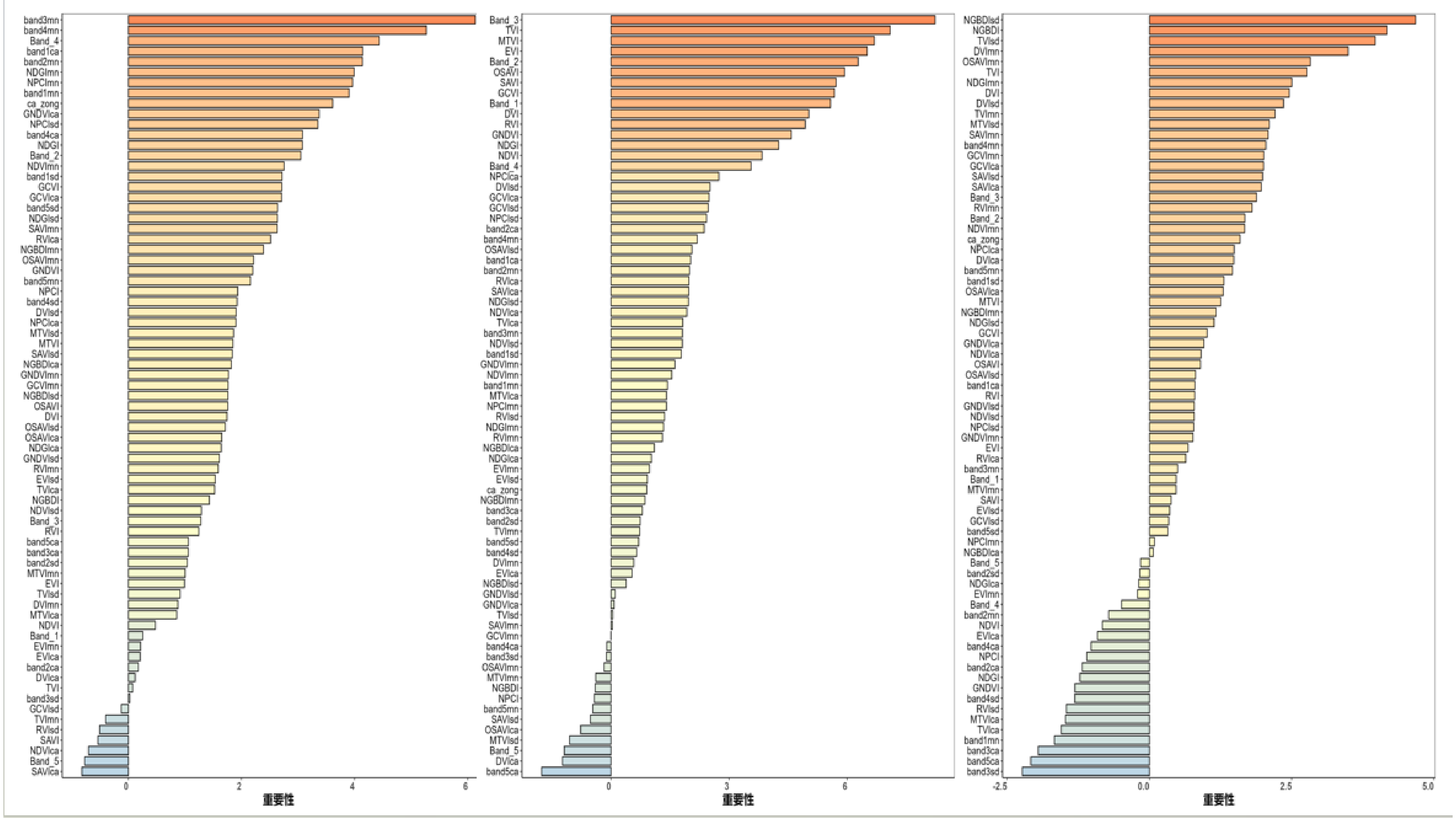

The performance of Random Forest models constructed for subtropical evergreen broad-leaved forest, grassland, and shrubland in sequence.

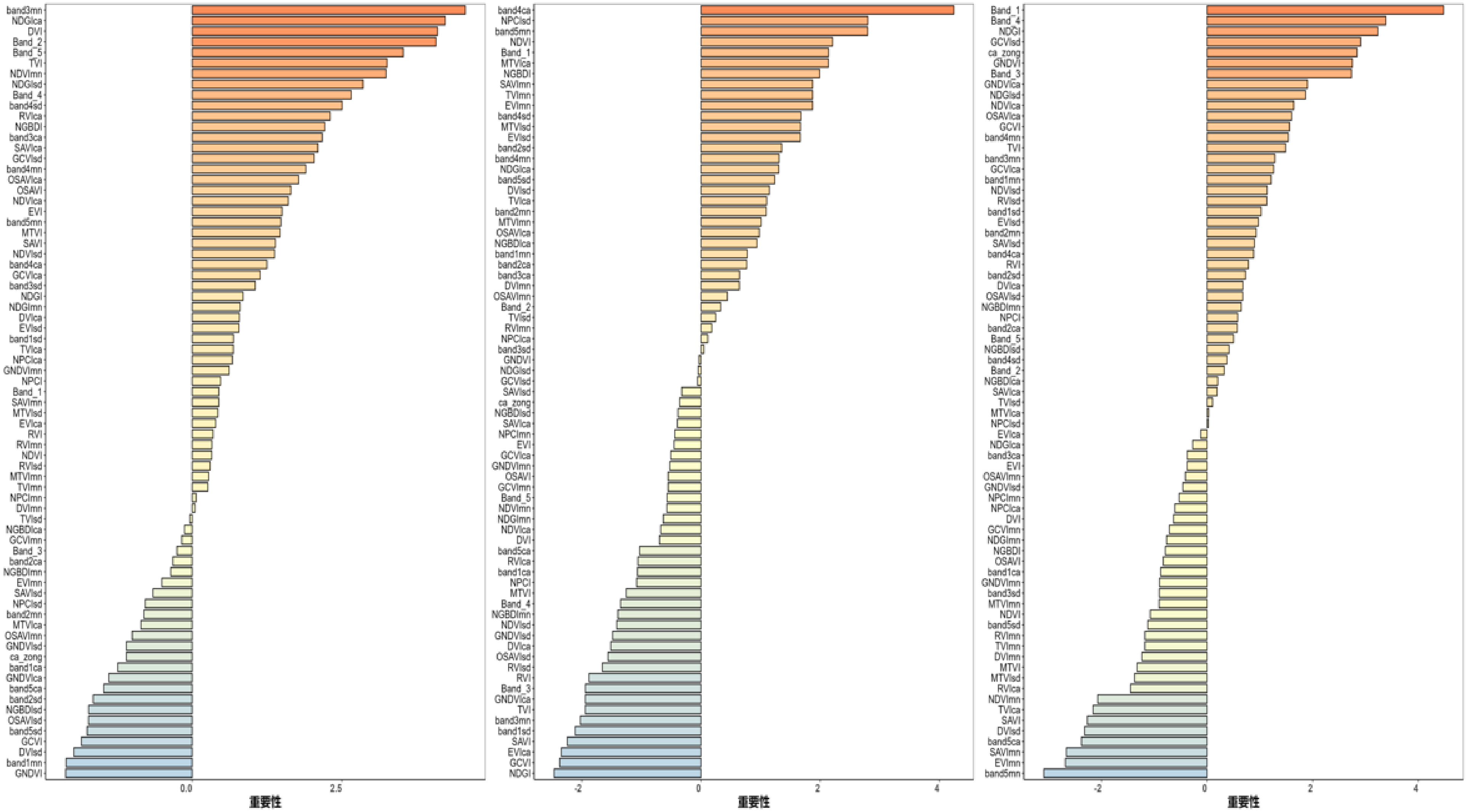

The performance of Random Forest models constructed for coniferous forests with low, medium, and high richness gradients in sequence.

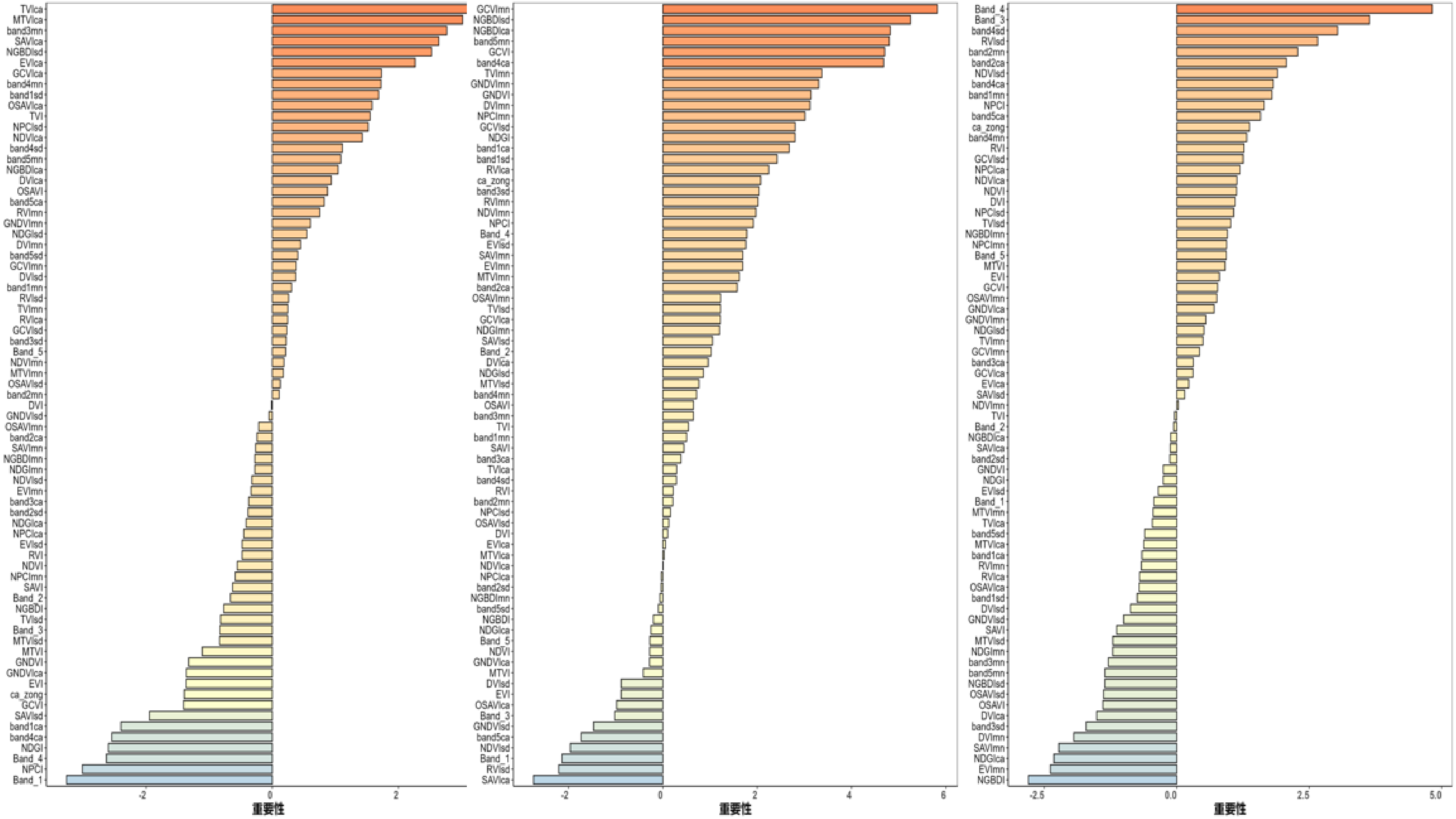

The performance of Random Forest models constructed for coniferous forests with low, medium, and high NDVI gradients in sequence.

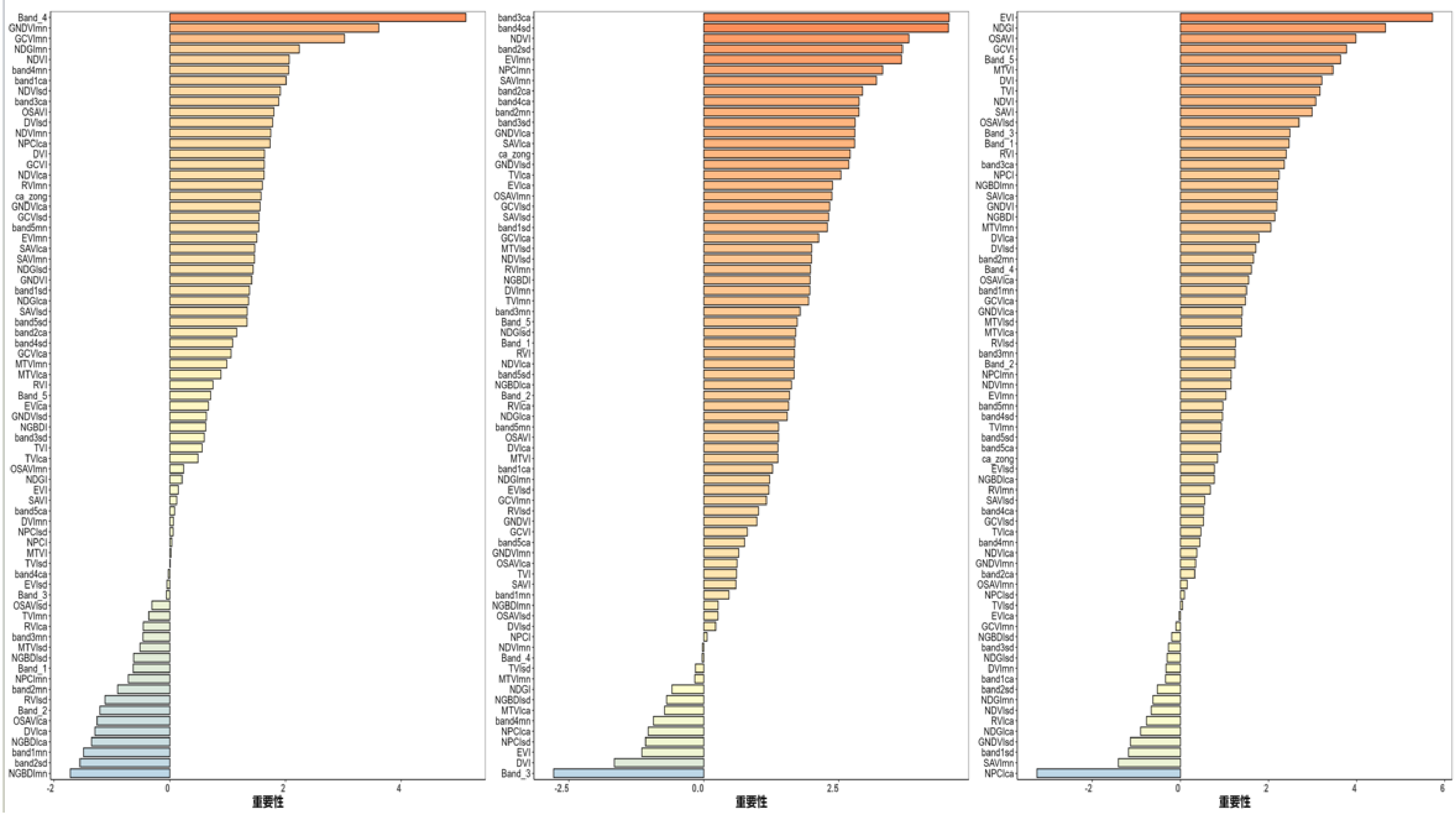

The performance of Random Forest models constructed for deciduous broad-leaved forests with low, medium, and high richness gradients in sequence.

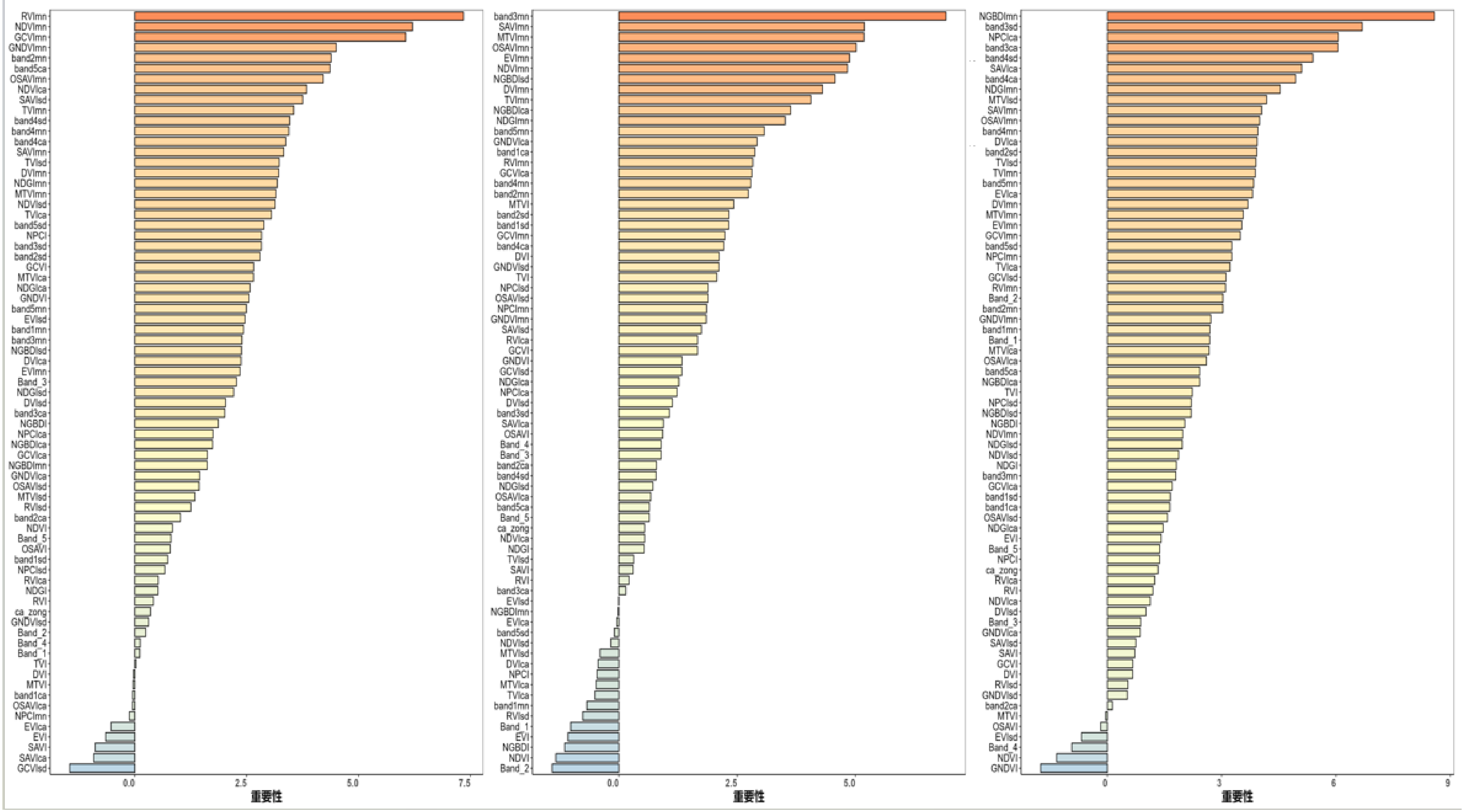

The performance of Random Forest models constructed for deciduous broad-leaved forests with low, medium, and high NDVI gradients in sequence.

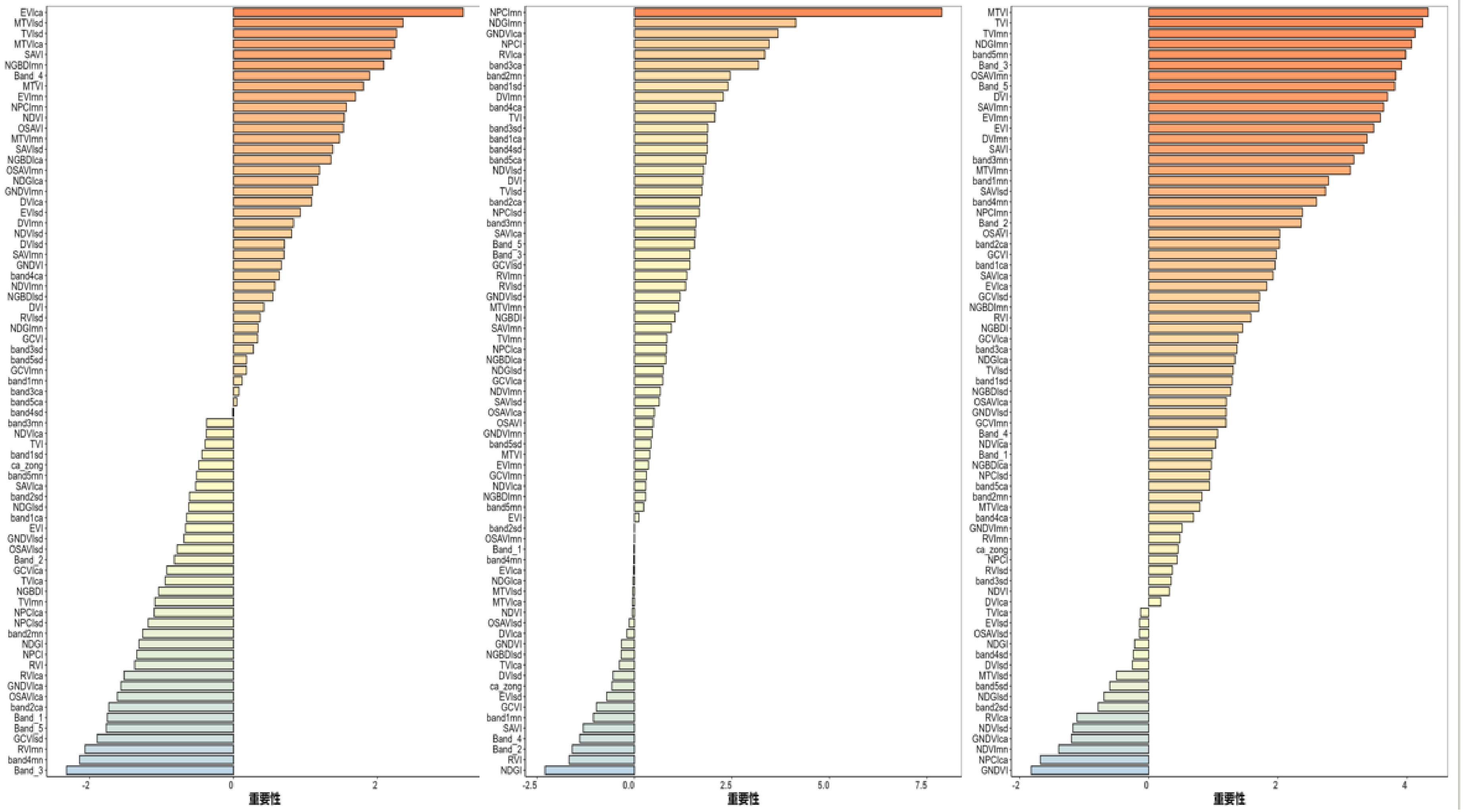

The performance of Random Forest models constructed for coniferous-broad-leaved mixed forests with low, medium, and high richness gradients in sequence.

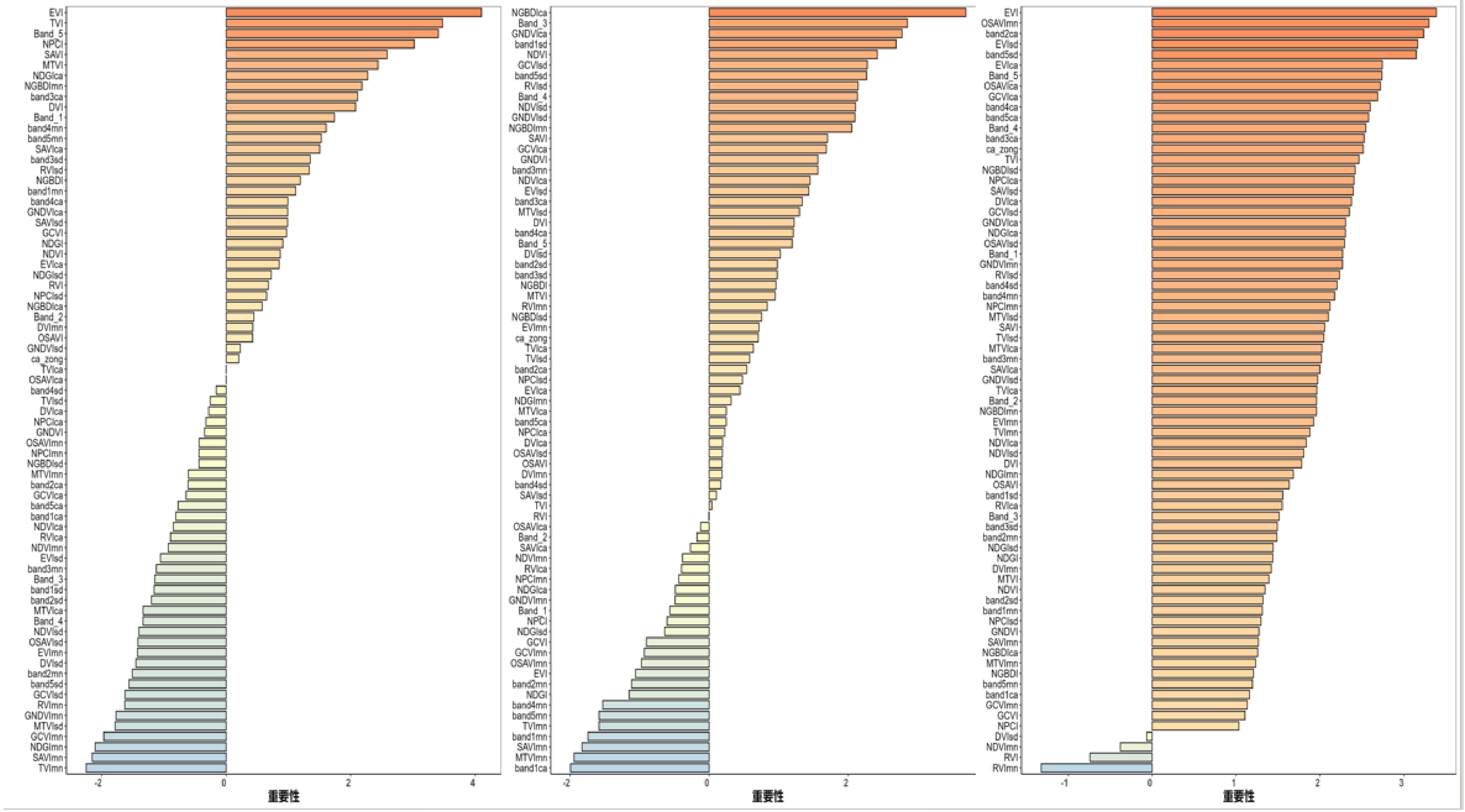

The performance of Random Forest models constructed for coniferous-broad-leaved mixed forests with low, medium, and high NDVI gradients in sequence.

Table S1 The importance value of each spectral index at each vegetation type and subgradient level in the random forest model constructed based on Sentinel-2 data (the last column indicates the number of repeats selected as the top five high importance indexes in all models)

**Sentinel 2**		**Coniferous forest**	**DBF**	**C&B mixed forest**	**EBF**	**grassland**	**shrubs**	frequency
**band1**	**gradients**	1.87	6.53	2.78	-0.10	-0.18	-1.22	2
band1	richness_L	-0.27	-0.49	0.57				
band1	richness_M	1.13	0.26	-0.49				
band1	richness_H	-0.95	-0.64	0.80				
band1	NDVI_L	-1.67	2.96	0.88				
band1	NDVI_M	4.41	4.38	2.38				
band1	NDVI_H	2.44	3.75	2.24				
**band1ca**	**gradients**	1.70	1.49	1.24	0.56	-1.15	0.25	
band1ca	richness_L	-0.49	-0.25	1.51				
band1ca	richness_M	2.53	0.48	0.02				
band1ca	richness_H	0.65	0.90	2.12				
band1ca	NDVI_L	-0.61	2.28	0.64				
band1ca	NDVI_M	1.90	1.52	0.96				
band1ca	NDVI_H	-2.20	-1.05	0.87				
**band1mn**	**gradients**	10.21	10.41	1.08	-0.68	-0.18	-1.68	5
band1mn	richness_L	1.89	0.11	0.03				
band1mn	richness_M	0.76	-0.48	0.66				
band1mn	richness_H	3.65	0.72	-0.01				
band1mn	NDVI_L	1.27	3.33	-0.15				
band1mn	NDVI_M	5.28	7.81	0.94				
band1mn	NDVI_H	0.88	1.88	1.15				
**band1sd**	**gradients**	2.91	2.11	4.66	0.91	1.01	-0.83	
band1sd	richness_L	0.16	-1.15	1.15				
band1sd	richness_M	0.46	1.10	-0.43				
band1sd	richness_H	0.36	1.26	1.87				
band1sd	NDVI_L	-1.00	2.01	0.61				
band1sd	NDVI_M	0.88	2.20	-0.61				

band1sd	NDVI_H	-1.62	0.09	3.24				
**band2**	**gradients**	1.50	2.72	2.36	0.82	0.60	1.32	
band2	richness_L	-0.63	1.50	0.01				
band2	richness_M	-1.98	0.13	1.01				
band2	richness_H	2.18	1.18	1.53				
band2	NDVI_L	-2.12	1.55	-0.65				
band2	NDVI_M	2.91	-1.87	-2.60				
band2	NDVI_H	1.17	2.86	1.78				
**band2ca**	**gradients**	1.15	1.54	0.17	2.07	1.00	-0.96	
band2ca	richness_L	-0.70	1.62	1.87				
band2ca	richness_M	0.22	0.17	-0.33				
band2ca	richness_H	-0.13	3.89	2.09				
band2ca	NDVI_L	-0.10	1.23	0.34				
band2ca	NDVI_M	1.23	1.11	1.72				
band2ca	NDVI_H	-1.43	0.44	1.25				
**band2mn**	**gradients**	1.86	2.53	5.08	-1.48	-0.48	-0.02	2
band2mn	richness_L	2.38	-1.32	-0.27				
band2mn	richness_M	0.99	-1.06	1.09				
band2mn	richness_H	0.26	1.50	2.46				
band2mn	NDVI_L	1.60	2.21	2.29				
band2mn	NDVI_M	3.12	1.32	0.32				
band2mn	NDVI_H	1.17	1.69	4.06				
**band2sd**	**gradients**	1.60	1.30	3.69	1.54	0.85	-2.00	
band2sd	richness_L	-0.12	0.33	0.93				
band2sd	richness_M	0.28	1.07	-0.10				
band2sd	richness_H	-0.43	3.33	1.80				
band2sd	NDVI_L	0.32	0.85	1.58				
band2sd	NDVI_M	-0.64	0.20	-0.68				
band2sd	NDVI_H	1.54	0.75	3.60				
**band3**	**gradients**	-0.11	1.40	-0.88	1.08	-0.57	-0.62	
band3	richness_L	2.10	-1.16	1.85				

band3	richness_M	1.68	-0.68	-1.57				
band3	richness_H	5.63	0.72	-0.94				
band3	NDVI_L	-1.57	0.11	1.17				
band3	NDVI_M	-0.39	-0.10	-0.36				
band3	NDVI_H	-1.03	-1.77	0.84				
**band3ca**	**gradients**	5.40	0.94	-0.44	1.06	3.29	-1.45	
band3ca	richness_L	-1.76	-1.78	0.54				
band3ca	richness_M	0.91	-0.45	1.28				
band3ca	richness_H	1.21	-0.13	2.59				
band3ca	NDVI_L	-0.16	1.38	0.98				
band3ca	NDVI_M	1.71	1.95	-1.27				
band3ca	NDVI_H	0.86	2.67	-1.70				
**band3mn**	**gradients**	1.29	2.99	3.13	4.09	-0.96	-0.25	2
band3mn	richness_L	-0.10	-0.09	-0.61				
band3mn	richness_M	-0.36	-1.26	-1.14				
band3mn	richness_H	1.52	1.63	3.47				
band3mn	NDVI_L	2.00	2.79	-0.93				
band3mn	NDVI_M	-1.58	0.91	-0.70				
band3mn	NDVI_H	0.36	0.54	0.30				
**band3sd**	**gradients**	4.05	1.96	-1.65	1.55	0.32	0.22	
band3sd	richness_L	-2.19	0.06	0.27				
band3sd	richness_M	0.84	0.47	2.13				
band3sd	richness_H	-0.89	1.16	2.83				
band3sd	NDVI_L	-0.98	2.65	1.32				
band3sd	NDVI_M	1.76	1.26	1.85				
band3sd	NDVI_H	1.44	1.84	-1.19				
**band4**	**gradients**	-0.65	1.53	3.58	2.13	0.91	-0.95	
band4	richness_L	0.17	0.45	-0.27				
band4	richness_M	0.34	-1.86	0.46				
band4	richness_H	0.76	0.17	2.24				
band4	NDVI_L	-1.36	1.34	-0.32				

band4	NDVI_M	0.06	-0.84	0.10				
band4	NDVI_H	2.02	-1.78	2.94				
**band4ca**	**gradients**	3.77	1.58	0.37	2.02	1.44	-1.27	
band4ca	richness_L	0.60	-0.76	-0.27				
band4ca	richness_M	-0.57	1.03	1.69				
band4ca	richness_H	0.87	4.13	0.32				
band4ca	NDVI_L	-0.82	1.59	1.00				
band4ca	NDVI_M	1.11	3.60	-1.61				
band4ca	NDVI_H	-0.77	0.70	-0.07				
**band4mn**	**gradients**	3.55	2.80	4.53	-1.50	-0.71	0.21	3
band4mn	richness_L	1.86	0.77	0.12				
band4mn	richness_M	1.21	0.11	0.39				
band4mn	richness_H	1.25	1.75	3.82				
band4mn	NDVI_L	1.87	2.25	-0.58				
band4mn	NDVI_M	1.18	3.07	-0.27				
band4mn	NDVI_H	0.60	1.06	4.07				
**band4sd**	**gradients**	1.82	2.16	1.50	1.22	1.08	0.08	
band4sd	richness_L	-0.10	0.21	0.91				
band4sd	richness_M	1.74	-1.42	1.72				
band4sd	richness_H	2.24	3.72	1.99				
band4sd	NDVI_L	0.92	1.40	0.85				
band4sd	NDVI_M	0.83	2.81	-1.04				
band4sd	NDVI_H	2.13	-1.20	-0.34				
**band5**	**gradients**	0.52	4.92	-0.60	0.99	1.78	0.01	
band5	richness_L	1.22	0.72	-0.95				
band5	richness_M	-0.28	2.01	1.67				
band5	richness_H	1.22	2.20	-0.95				
band5	NDVI_L	-0.31	1.79	-0.35				
band5	NDVI_M	2.86	0.98	-0.70				
band5	NDVI_H	1.04	1.89	1.11				
**band5ca**	**gradients**	1.17	4.06	-0.13	0.32	2.65	1.66	

band5ca	richness_L	1.93	-0.22	0.54			
band5ca	richness_M	-0.94	1.43	-0.41			
band5ca	richness_H	-0.46	0.79	1.36			
band5ca	NDVI_L	2.64	1.82	0.39			
band5ca	NDVI_M	-1.18	-0.05	0.38			
band5ca	NDVI_H	2.42	0.41	1.13			
**band5mn**	**gradients**	-0.85	1.48	2.15	-0.94	1.56	2.27
band5mn	richness_L	1.76	1.15	0.95			
band5mn	richness_M	2.73	2.29	1.37			
band5mn	richness_H	-0.30	0.03	1.30			
band5mn	NDVI_L	2.11	2.03	1.68			
band5mn	NDVI_M	2.23	1.73	2.05			
band5mn	NDVI_H	0.17	0.65	1.02			
**band5sd**	**gradients**	-0.23	2.82	1.74	0.41	1.41	0.24
band5sd	richness_L	2.34	-0.98	1.27			
band5sd	richness_M	-1.03	0.98	1.15			
band5sd	richness_H	0.38	3.43	-1.18			
band5sd	NDVI_L	-0.67	1.34	-1.08			
band5sd	NDVI_M	-0.74	0.27	1.17			
band5sd	NDVI_H	-0.51	-0.86	-0.37			
**band6**	**gradients**	1.41	4.74	-0.97	0.41	0.48	1.13
band6	richness_L	1.84	0.97	1.19			
band6	richness_M	-1.89	0.45	-1.28			
band6	richness_H	1.84	2.28	2.05			
band6	NDVI_L	-0.38	0.85	-0.98			
band6	NDVI_M	1.03	2.28	0.52			
band6	NDVI_H	0.24	-0.39	-1.19			
**band6ca**	**gradients**	0.29	1.15	1.02	1.31	1.00	1.71
band6ca	richness_L	-1.11	0.76	-0.65			
band6ca	richness_M	-0.70	1.18	-1.70			
band6ca	richness_H	1.11	2.63	1.31			

band6ca	NDVI_L	-0.62	1.90	-1.67			
band6ca	NDVI_M	0.02	1.47	-0.70			
band6ca	NDVI_H	-0.80	1.09	0.31			
**band6mn**	**gradients**	-0.65	2.24	1.33	1.03	2.35	3.03
band6mn	richness_L	2.33	-0.15	1.92			
band6mn	richness_M	0.15	1.67	1.50			
band6mn	richness_H	-0.33	1.03	1.59			
band6mn	NDVI_L	1.72	1.37	1.29			
band6mn	NDVI_M	2.21	-0.69	0.16			
band6mn	NDVI_H	0.91	-1.31	1.43			
**band6sd**	**gradients**	-0.26	3.45	-0.43	-0.30	0.93	1.54
band6sd	richness_L	-0.09	-0.74	-0.39			
band6sd	richness_M	0.39	0.38	-1.15			
band6sd	richness_H	0.75	1.66	0.53			
band6sd	NDVI_L	-0.47	2.06	0.49			
band6sd	NDVI_M	-1.47	-0.86	-1.13			
band6sd	NDVI_H	-1.39	-1.38	1.24			
**band7**	**gradients**	2.66	1.74	-1.97	2.07	0.05	1.46
band7	richness_L	2.52	1.49	0.25			
band7	richness_M	-1.50	0.63	0.24			
band7	richness_H	0.95	1.83	2.57			
band7	NDVI_L	-0.01	1.38	-1.13			
band7	NDVI_M	0.47	0.20	2.00			
band7	NDVI_H	-2.11	0.01	1.25			
**band7ca**	**gradients**	0.37	1.77	0.54	-0.36	-1.08	-2.52
band7ca	richness_L	-0.35	0.15	1.47			
band7ca	richness_M	1.77	0.83	-2.09			
band7ca	richness_H	1.84	2.72	-0.46			
band7ca	NDVI_L	1.29	2.66	-0.20			
band7ca	NDVI_M	-0.37	2.63	0.00			
band7ca	NDVI_H	0.63	2.64	-1.42			

**band7mn**	**gradients**	1.07	1.81	2.53	-0.64	1.70	3.12	2
band7mn	richness_L	2.97	0.10	2.62				
band7mn	richness_M	1.02	2.35	2.09				
band7mn	richness_H	0.00	0.68	0.98				
band7mn	NDVI_L	0.41	2.49	2.41				
band7mn	NDVI_M	-0.62	-0.05	3.47				
band7mn	NDVI_H	-0.27	-0.94	0.86				
**band7sd**	**gradients**	-0.62	3.43	0.98	-0.21	2.40	-0.12	
band7sd	richness_L	1.60	-0.18	0.69				
band7sd	richness_M	-1.08	0.67	-1.90				
band7sd	richness_H	-0.30	0.84	0.08				
band7sd	NDVI_L	1.14	1.18	1.23				
band7sd	NDVI_M	-2.28	0.18	2.24				
band7sd	NDVI_H	0.81	0.12	1.60				
**ca_zong**	**gradients**	0.32	2.47	-0.83	-1.22	2.45	0.29	
ca_zong	richness_L	0.56	-0.84	0.25				
ca_zong	richness_M	1.84	1.47	0.75				
ca_zong	richness_H	0.82	2.86	0.97				
ca_zong	NDVI_L	2.04	1.35	-0.59				
ca_zong	NDVI_M	1.18	1.87	0.86				
ca_zong	NDVI_H	0.57	1.08	-0.87				
**DVIca**	**gradients**	1.50	1.77	1.98	-1.85	-0.56	-0.55	
DVIca	richness_L	-0.25	-2.52	-1.23				
DVIca	richness_M	-0.33	-0.24	-0.46				
DVIca	richness_H	-0.73	1.01	1.63				
DVIca	NDVI_L	1.13	2.73	0.87				
DVIca	NDVI_M	-0.73	-0.25	-0.86				
DVIca	NDVI_H	0.80	2.17	1.07				
**DVImn**	**gradients**	-0.45	0.69	1.81	0.83	1.16	0.95	
DVImn	richness_L	2.54	0.92	3.44				
DVImn	richness_M	0.66	1.15	1.51				

DVImn	richness_H	0.98	0.40	-0.79				
DVImn	NDVI_L	1.31	2.39	1.26				
DVImn	NDVI_M	-0.46	0.30	0.12				
DVImn	NDVI_H	1.01	0.79	-1.22				
**DVIsd**	**gradients**	0.91	2.47	2.10	-1.18	0.88	2.82	2
DVIsd	richness_L	-0.28	-0.62	-1.45				
DVIsd	richness_M	3.00	-1.69	0.91				
DVIsd	richness_H	-0.06	1.47	-1.03				
DVIsd	NDVI_L	0.98	1.52	2.89				
DVIsd	NDVI_M	-0.51	0.87	0.57				
DVIsd	NDVI_H	-1.09	1.23	-1.45				
**EVIca**	**gradients**	1.22	3.58	0.40	0.17	-0.85	-0.79	
EVIca	richness_L	-1.49	0.80	3.11				
EVIca	richness_M	0.68	1.50	-0.88				
EVIca	richness_H	1.88	2.44	-1.39				
EVIca	NDVI_L	1.15	2.33	0.63				
EVIca	NDVI_M	-0.53	-1.34	-0.37				
EVIca	NDVI_H	-0.82	2.60	1.97				
**EVImn**	**gradients**	1.88	3.25	1.53	1.24	1.16	1.69	
EVImn	richness_L	1.60	1.82	2.22				
EVImn	richness_M	1.78	1.16	1.77				
EVImn	richness_H	-0.82	1.11	-0.84				
EVImn	NDVI_L	1.79	2.24	0.18				
EVImn	NDVI_M	0.79	-0.41	1.63				
EVImn	NDVI_H	1.30	0.99	0.93				
**EVIsd**	**gradients**	-0.29	4.47	3.05	0.00	-0.66	1.53	2
EVIsd	richness_L	-0.62	0.75	-1.65				
EVIsd	richness_M	1.84	1.08	1.38				
EVIsd	richness_H	0.80	0.92	0.89				
EVIsd	NDVI_L	2.19	0.43	2.63				
EVIsd	NDVI_M	-1.33	4.45	-0.04				

EVIsd	NDVI_H	4.32	2.44	1.44			
**GCVI**	**gradients**	4.09	1.61	0.39	-1.57	0.94	-1.08
GCVI	richness_L	-1.62	0.90	0.46			
GCVI	richness_M	0.13	-1.25	1.66			
GCVI	richness_H	-1.18	-1.42	1.96			
GCVI	NDVI_L	1.42	1.92	-1.21			
GCVI	NDVI_M	2.21	-1.09	0.49			
GCVI	NDVI_H	-0.06	0.22	0.88			
**GCVIca**	**gradients**	2.17	3.33	-0.88	-1.20	1.86	1.42
GCVIca	richness_L	3.93	-2.09	-0.24			
GCVIca	richness_M	-0.61	-0.72	-0.62			
GCVIca	richness_H	1.73	0.40	0.45			
GCVIca	NDVI_L	-1.80	2.60	1.83			
GCVIca	NDVI_M	-0.29	1.95	-1.53			
GCVIca	NDVI_H	0.02	-0.12	0.56			
**GCVImn**	**gradients**	1.05	2.46	0.42	-1.19	-0.37	-1.29
GCVImn	richness_L	1.58	0.19	-0.87			
GCVImn	richness_M	1.56	-0.01	-1.23			
GCVImn	richness_H	0.36	2.08	0.54			
GCVImn	NDVI_L	4.36	0.96	0.88			
GCVImn	NDVI_M	0.95	1.92	0.81			
GCVImn	NDVI_H	0.13	0.72	-2.23			
**GCVIsd**	**gradients**	3.65	1.08	1.61	-1.97	-0.69	0.86
GCVIsd	richness_L	2.57	-0.80	-2.11			
GCVIsd	richness_M	1.03	-1.18	-1.71			
GCVIsd	richness_H	0.96	1.75	1.70			
GCVIsd	NDVI_L	1.23	2.05	2.12			
GCVIsd	NDVI_M	1.37	0.97	0.55			
GCVIsd	NDVI_H	-1.11	1.19	-0.76			
**GNDVIca**	**gradients**	1.61	1.42	1.90	-1.22	0.45	-0.53
GNDVIca	richness_L	0.48	0.51	1.66			

GNDVIca	richness_M	-1.98	-0.23	-0.72				
GNDVIca	richness_H	1.27	0.89	-0.49				
GNDVIca	NDVI_L	-0.77	1.68	2.40				
GNDVIca	NDVI_M	2.06	1.83	0.40				
GNDVIca	NDVI_H	-1.85	1.61	0.82				
**GNDVImn**	**gradients**	-1.65	2.31	0.08	0.96	-1.25	-1.18	2
GNDVImn	richness_L	0.40	-0.17	3.99				
GNDVImn	richness_M	1.16	-0.29	2.85				
GNDVImn	richness_H	-0.90	1.45	0.77				
GNDVImn	NDVI_L	1.19	-0.31	2.14				
GNDVImn	NDVI_M	-0.03	0.34	1.04				
GNDVImn	NDVI_H	-0.71	2.18	0.83				
**GNDVIsd**	**gradients**	2.77	2.67	2.20	-1.21	1.42	-0.04	
GNDVIsd	richness_L	1.36	0.08	-0.41				
GNDVIsd	richness_M	-0.85	-0.96	-0.02				
GNDVIsd	richness_H	0.35	0.61	-0.07				
GNDVIsd	NDVI_L	0.05	1.46	3.31				
GNDVIsd	NDVI_M	-1.26	2.77	-0.62				
GNDVIsd	NDVI_H	-0.01	3.39	0.57				
**MCARIca**	**gradients**	2.08	3.76	0.05	0.82	-0.24	2.15	
MCARIca	richness_L	1.31	-1.60	-1.46				
MCARIca	richness_M	2.21	0.83	-0.99				
MCARIca	richness_H	1.55	3.22	0.84				
MCARIca	NDVI_L	0.18	0.07	1.75				
MCARIca	NDVI_M	2.00	3.95	-0.37				
MCARIca	NDVI_H	0.82	0.32	0.84				
**MCARImn**	**gradients**	2.47	4.28	1.94	0.10	-0.37	1.23	
MCARImn	richness_L	3.21	3.06	2.94				
MCARImn	richness_M	0.69	-0.20	1.09				
MCARImn	richness_H	0.32	3.42	1.33				
MCARImn	NDVI_L	0.07	1.11	1.73				

MCARImn	NDVI_M	1.63	4.22	-1.60				
MCARImn	NDVI_H	-0.32	1.15	0.96				
**MCARIsd**	**gradients**	2.07	1.72	2.54	-0.08	0.29	-0.93	
MCARIsd	richness_L	0.01	-0.11	-0.46				
MCARIsd	richness_M	-0.31	0.26	0.55				
MCARIsd	richness_H	-0.61	2.20	0.65				
MCARIsd	NDVI_L	0.09	1.46	-1.26				
MCARIsd	NDVI_M	0.27	2.77	-1.02				
MCARIsd	NDVI_H	1.69	2.67	1.52				
**MTVI**	**gradients**	2.29	3.52	0.93	-0.92	1.52	0.69	2
MTVI	richness_L	2.01	-1.05	0.84				
MTVI	richness_M	0.12	3.06	1.52				
MTVI	richness_H	0.77	1.64	0.32				
MTVI	NDVI_L	0.62	0.48	0.02				
MTVI	NDVI_M	2.92	-1.36	2.65				
MTVI	NDVI_H	1.39	0.88	0.53				
**MTVIca**	**gradients**	3.22	2.23	1.50	2.10	0.50	-0.87	3
MTVIca	richness_L	4.22	2.87	-0.77				
MTVIca	richness_M	2.19	4.30	0.10				
MTVIca	richness_H	-0.85	0.79	-0.74				
MTVIca	NDVI_L	-1.67	4.43	2.59				
MTVIca	NDVI_M	-0.40	-0.81	-0.17				
MTVIca	NDVI_H	1.87	1.58	0.92				
**MTVImm**	**gradients**	2.21	1.58	0.62	1.43	2.02	1.70	2
MTVImm	richness_L	0.54	-1.21	0.73				
MTVImm	richness_M	0.09	3.10	0.66				
MTVImm	richness_H	2.27	0.96	-0.77				
MTVImm	NDVI_L	1.81	2.04	0.94				
MTVImm	NDVI_M	1.68	1.06	-0.18				
MTVImm	NDVI_H	2.96	5.85	0.41				
**MTVIsd**	**gradients**	6.71	2.43	1.55	-0.31	0.96	1.93	2

MTVIsd	richness_L	0.97	-0.86	-0.39				
MTVIsd	richness_M	-1.16	1.07	-0.41				
MTVIsd	richness_H	2.33	0.76	-0.17				
MTVIsd	NDVI_L	0.68	2.80	1.60				
MTVIsd	NDVI_M	-0.18	1.10	-0.11				
MTVIsd	NDVI_H	4.25	1.32	0.56				
**NDGIca**	**gradients**	1.83	1.53	-0.69	-0.65	1.80	-0.89	
NDGIca	richness_L	-0.98	1.25	0.10				
NDGIca	richness_M	0.14	2.26	0.89				
NDGIca	richness_H	-0.90	0.66	0.70				
NDGIca	NDVI_L	3.72	0.88	2.22				
NDGIca	NDVI_M	1.72	3.56	1.35				
NDGIca	NDVI_H	2.34	1.32	1.01				
**NDGImn**	**gradients**	1.26	10.47	1.13	1.80	0.78	1.28	3
NDGImn	richness_L	-0.83	1.47	1.34				
NDGImn	richness_M	0.25	0.11	1.92				
NDGImn	richness_H	0.89	1.35	2.17				
NDGImn	NDVI_L	3.44	2.60	1.87				
NDGImn	NDVI_M	3.74	-0.12	1.37				
NDGImn	NDVI_H	1.55	5.06	-0.46				
**NDGIsd**	**gradients**	2.69	1.89	0.13	0.66	1.04	0.68	
NDGIsd	richness_L	-0.14	-0.65	0.33				
NDGIsd	richness_M	-0.80	1.74	3.01				
NDGIsd	richness_H	2.24	0.09	2.47				
NDGIsd	NDVI_L	3.09	2.29	-1.84				
NDGIsd	NDVI_M	-0.75	2.96	-0.89				
NDGIsd	NDVI_H	4.12	0.63	0.83				
**NDREI**	**gradients**	2.67	1.62	0.84	0.29	0.83	-0.21	
NDREI	richness_L	-0.06	-0.79	-1.53				
NDREI	richness_M	-2.74	0.59	2.54				
NDREI	richness_H	2.66	1.37	1.46				

NDREI	NDVI_L	-0.61	0.13	0.76			
NDREI	NDVI_M	2.43	1.91	0.40			
NDREI	NDVI_H	-0.67	0.04	0.21			
**NDREIca**	**gradients**	1.55	1.53	-0.02	0.64	-0.62	1.41
NDREIca	richness_L	1.80	0.95	0.46			
NDREIca	richness_M	-2.67	1.89	-0.93			
NDREIca	richness_H	-0.30	-0.51	-1.08			
NDREIca	NDVI_L	0.29	2.28	0.55			
NDREIca	NDVI_M	0.84	-0.36	2.33			
NDREIca	NDVI_H	1.41	0.37	1.92			
**NDREImn**	**gradients**	0.11	3.03	1.23	1.47	-0.53	0.21
NDREImn	richness_L	-0.36	-0.41	-0.14			
NDREImn	richness_M	2.09	-0.87	2.41			
NDREImn	richness_H	1.20	0.82	1.57			
NDREImn	NDVI_L	1.02	0.45	1.15			
NDREImn	NDVI_M	1.05	2.00	-1.30			
NDREImn	NDVI_H	1.18	0.88	1.01			
**NDREIsd**	**gradients**	0.32	0.58	1.67	1.53	-0.21	2.12
NDREIsd	richness_L	2.20	0.90	-0.88			
NDREIsd	richness_M	1.22	-0.90	-0.14			
NDREIsd	richness_H	2.29	1.88	1.44			
NDREIsd	NDVI_L	1.00	1.93	0.79			
NDREIsd	NDVI_M	-0.24	-0.79	-1.62			
NDREIsd	NDVI_H	-0.22	0.48	-0.63			
**NDVIca**	**gradients**	2.19	1.31	1.76	-1.27	0.31	-0.52
NDVIca	richness_L	-0.57	0.30	1.89			
NDVIca	richness_M	2.53	0.18	0.16			
NDVIca	richness_H	-0.86	0.20	0.69			
NDVIca	NDVI_L	-0.41	2.47	0.38			
NDVIca	NDVI_M	0.20	3.92	0.81			
NDVIca	NDVI_H	-0.86	2.54	1.32			

**NDVImn**	**gradients**	0.38	0.84	1.85	-0.05	1.93	0.60	
NDVImn	richness_L	2.36	-0.79	3.44				
NDVImn	richness_M	1.47	1.74	1.83				
NDVImn	richness_H	-0.07	0.23	0.69				
NDVImn	NDVI_L	1.23	0.92	1.41				
NDVImn	NDVI_M	0.99	-0.81	1.68				
NDVImn	NDVI_H	-1.47	-0.34	-1.16				
**NDVIsd**	**gradients**	-0.39	2.09	2.96	-0.74	1.75	1.30	
NDVIsd	richness_L	0.92	0.39	1.88				
NDVIsd	richness_M	1.49	2.33	0.06				
NDVIsd	richness_H	-0.01	1.87	0.03				
NDVIsd	NDVI_L	-0.19	1.84	1.86				
NDVIsd	NDVI_M	0.56	1.28	-1.10				
NDVIsd	NDVI_H	1.68	0.60	-1.91				
**NGBDIca**	**gradients**	3.02	3.16	-1.80	-0.27	1.85	1.89	2
NGBDIca	richness_L	-1.14	1.19	0.05				
NGBDIca	richness_M	1.91	1.16	-0.60				
NGBDIca	richness_H	0.39	4.03	0.17				
NGBDIca	NDVI_L	-0.32	0.14	-0.65				
NGBDIca	NDVI_M	-1.10	-0.56	2.22				
NGBDIca	NDVI_H	10.71	1.83	-0.27				
**NGBDImn**	**gradients**	4.19	5.30	4.16	0.54	2.96	-0.23	2
NGBDImn	richness_L	2.03	1.34	-0.94				
NGBDImn	richness_M	-1.00	-0.67	0.91				
NGBDImn	richness_H	0.96	3.33	1.90				
NGBDImn	NDVI_L	2.24	1.82	2.33				
NGBDImn	NDVI_M	0.41	7.01	-1.85				
NGBDImn	NDVI_H	2.74	0.36	2.16				
**NGBDIsd**	**gradients**	1.91	4.41	1.66	0.13	3.56	-1.31	3
NGBDIsd	richness_L	2.26	3.47	1.71				
NGBDIsd	richness_M	0.28	2.77	-0.18				

NGBDIsd	richness_H	1.93	0.53	2.48				
NGBDIsd	NDVI_L	-1.02	2.13	-0.63				
NGBDIsd	NDVI_M	1.53	3.53	1.15				
NGBDIsd	NDVI_H	1.97	1.01	0.85				
**NPCI**	**gradients**	1.63	7.40	1.90	2.67	0.18	0.35	3
NPCI	richness_L	1.02	-0.12	2.32				
NPCI	richness_M	0.53	-0.29	2.37				
NPCI	richness_H	1.46	3.53	-0.62				
NPCI	NDVI_L	-0.39	2.65	1.18				
NPCI	NDVI_M	2.94	4.24	-1.39				
NPCI	NDVI_H	0.45	-0.79	1.72				
**NPCIca**	**gradients**	14.74	5.12	9.21	6.84	1.81	1.54	6
NPCIca	richness_L	1.15	1.25	1.99				
NPCIca	richness_M	-0.95	-1.36	-0.67				
NPCIca	richness_H	0.90	0.55	7.58				
NPCIca	NDVI_L	3.08	1.70	-1.83				
NPCIca	NDVI_M	1.90	1.63	0.70				
NPCIca	NDVI_H	11.64	2.19	8.47				
**NPCImn**	**gradients**	4.25	9.84	-0.27	4.07	1.91	0.82	5
NPCImn	richness_L	0.11	1.57	2.77				
NPCImn	richness_M	-0.53	1.40	-2.11				
NPCImn	richness_H	1.31	0.94	-0.20				
NPCImn	NDVI_L	-1.31	4.42	-1.23				
NPCImn	NDVI_M	3.51	11.78	1.02				
NPCImn	NDVI_H	4.58	1.43	1.12				
**NPCIsd**	**gradients**	4.07	3.24	0.91	4.59	-0.54	1.39	2
NPCIsd	richness_L	0.77	-0.13	-0.04				
NPCIsd	richness_M	0.81	-0.11	0.07				
NPCIsd	richness_H	3.92	-0.30	0.84				
NPCIsd	NDVI_L	2.00	3.10	-1.35				
NPCIsd	NDVI_M	-0.99	1.19	-0.45				

NPCIsd	NDVI_H	1.33	2.07	-0.06				
**OSAVI**	**gradients**	5.44	3.27	-1.01	-0.80	1.93	-0.60	5
OSAVI	richness_L	2.01	-0.65	-1.76				
OSAVI	richness_M	-0.70	2.57	-0.64				
OSAVI	richness_H	-0.90	1.05	2.18				
OSAVI	NDVI_L	6.27	3.93	3.80				
OSAVI	NDVI_M	4.52	-1.16	0.06				
OSAVI	NDVI_H	0.01	-0.11	0.59				
**OSAVIca**	**gradients**	8.66	3.66	1.69	-0.57	-0.29	-0.56	2
OSAVIca	richness_L	3.08	-1.65	0.79				
OSAVIca	richness_M	-0.47	0.66	1.79				
OSAVIca	richness_H	-2.68	1.25	1.03				
OSAVIca	NDVI_L	9.38	3.61	0.52				
OSAVIca	NDVI_M	2.98	0.17	1.02				
OSAVIca	NDVI_H	-0.31	1.41	-0.89				
**OSAVImn**	**gradients**	6.62	4.79	-1.39	2.28	1.99	-1.40	3
OSAVImn	richness_L	-0.63	-0.44	-0.14				
OSAVImn	richness_M	0.63	1.44	-0.03				
OSAVImn	richness_H	0.08	2.09	-1.05				
OSAVImn	NDVI_L	10.02	3.90	4.09				
OSAVImn	NDVI_M	2.53	1.52	0.76				
OSAVImn	NDVI_H	1.43	-0.46	-0.27				
**OSAVIsd**	**gradients**	1.04	2.33	2.35	-1.02	-1.96	0.02	2
OSAVIsd	richness_L	4.67	-0.15	-1.26				
OSAVIsd	richness_M	0.82	0.55	-1.77				
OSAVIsd	richness_H	0.31	1.50	1.78				
OSAVIsd	NDVI_L	2.05	4.75	0.45				
OSAVIsd	NDVI_M	0.47	-0.16	1.23				
OSAVIsd	NDVI_H	0.32	0.17	-0.13				
**RVIca**	**gradients**	-2.42	2.27	0.86	-1.82	-0.55	2.36	
RVIca	richness_L	-0.73	0.40	-0.46				

RVIca	richness_M	-0.67	0.49	-1.41				
RVIca	richness_H	2.34	0.15	0.12				
RVIca	NDVI_L	-0.71	1.52	3.41				
RVIca	NDVI_M	-2.45	-0.23	1.53				
RVIca	NDVI_H	-0.74	1.80	1.73				
**RVImn**	**gradients**	1.12	1.44	-1.13	-0.75	-0.10	0.99	
RVImn	richness_L	2.08	0.75	2.16				
RVImn	richness_M	0.29	1.75	1.16				
RVImn	richness_H	-0.77	-0.03	0.37				
RVImn	NDVI_L	1.91	1.01	2.11				
RVImn	NDVI_M	2.47	1.24	1.86				
RVImn	NDVI_H	-1.20	-0.30	0.80				
**RVIsd**	**gradients**	0.37	3.83	0.43	-0.08	1.25	2.93	
RVIsd	richness_L	0.51	0.47	-1.06				
RVIsd	richness_M	1.70	-1.73	0.02				
RVIsd	richness_H	-0.23	2.70	-1.04				
RVIsd	NDVI_L	1.75	1.29	1.35				
RVIsd	NDVI_M	-1.23	4.40	1.61				
RVIsd	NDVI_H	1.72	1.93	0.59				
**SAVIcc**	**gradients**	2.22	2.55	1.08	-0.16	2.44	-1.37	
SAVIcc	richness_L	0.57	-1.17	-0.03				
SAVIcc	richness_M	0.11	2.22	1.06				
SAVIcc	richness_H	-0.49	1.77	-0.93				
SAVIcc	NDVI_L	-0.04	2.64	1.40				
SAVIcc	NDVI_M	-0.85	2.68	-0.09				
SAVIcc	NDVI_H	1.74	3.21	1.33				
**SAVImn**	**gradients**	2.06	7.87	-0.47	2.00	1.98	0.61	2
SAVImn	richness_L	3.46	0.72	1.19				
SAVImn	richness_M	-0.55	0.69	0.39				
SAVImn	richness_H	1.09	0.42	1.00				
SAVImn	NDVI_L	1.35	1.37	1.16				

SAVImn	NDVI_M	-0.28	-0.20	0.35				
SAVImn	NDVI_H	1.15	7.04	1.01				
**SAVIsd**	**gradients**	0.69	10.54	1.62	-1.20	1.87	2.74	4
SAVIsd	richness_L	-0.06	0.31	-1.08				
SAVIsd	richness_M	-0.58	1.84	-1.64				
SAVIsd	richness_H	0.97	0.31	-1.31				
SAVIsd	NDVI_L	0.35	-0.03	2.36				
SAVIsd	NDVI_M	-0.16	6.85	-0.80				
SAVIsd	NDVI_H	1.70	8.05	-0.06				
**SDVI**	**gradients**	1.62	1.93	0.22	0.03	0.63	0.61	
SDVI	richness_L	3.72	1.09	0.52				
SDVI	richness_M	-0.31	2.40	-2.29				
SDVI	richness_H	1.96	1.06	-0.12				
SDVI	NDVI_L	1.26	-0.51	-0.50				
SDVI	NDVI_M	0.87	1.34	-0.49				
SDVI	NDVI_H	-0.42	-1.87	-1.65				
**SEVI**	**gradients**	4.05	4.97	-0.10	-0.85	0.15	1.21	
SEVI	richness_L	4.44	0.38	1.86				
SEVI	richness_M	-2.15	1.08	-1.20				
SEVI	richness_H	2.53	1.23	0.25				
SEVI	NDVI_L	0.89	0.70	1.20				
SEVI	NDVI_M	2.74	2.33	0.29				
SEVI	NDVI_H	2.30	-0.88	0.91				
**SGNDVI**	**gradients**	1.77	0.86	-0.68	0.85	-1.38	1.64	
SGNDVI	richness_L	1.23	2.74	-0.78				
SGNDVI	richness_M	0.08	0.59	-0.12				
SGNDVI	richness_H	-1.34	1.00	1.62				
SGNDVI	NDVI_L	-1.01	0.05	-0.05				
SGNDVI	NDVI_M	0.65	-0.21	-2.06				
SGNDVI	NDVI_H	-1.30	1.61	-1.69				
**SMCARI**	**gradients**	0.62	3.14	1.19	0.16	-0.06	0.42	2

SMCARI	richness_L	0.88	10.54	-0.19				
SMCARI	richness_M	-0.15	-2.36	-1.09				
SMCARI	richness_H	-0.33	1.38	4.63				
SMCARI	NDVI_L	1.31	-1.25	-2.22				
SMCARI	NDVI_M	-1.35	0.92	0.96				
SMCARI	NDVI_H	-1.67	1.22	1.69				
**SNDGI**	**gradients**	2.09	4.72	5.76	0.99	-0.54	1.35	6
SNDGI	richness_L	1.36	9.64	1.31				
SNDGI	richness_M	-0.10	-0.45	-0.33				
SNDGI	richness_H	1.58	-1.25	5.34				
SNDGI	NDVI_L	0.31	0.48	0.71				
SNDGI	NDVI_M	4.48	-1.36	1.35				
SNDGI	NDVI_H	2.34	4.19	3.42				
**SNDVI**	**gradients**	0.79	1.12	-2.11	-0.07	-0.29	0.37	
SNDVI	richness_L	2.52	2.03	-0.70				
SNDVI	richness_M	-0.29	-0.45	-0.11				
SNDVI	richness_H	0.58	1.38	1.77				
SNDVI	NDVI_L	-0.64	2.03	-0.09				
SNDVI	NDVI_M	3.03	-0.27	0.93				
SNDVI	NDVI_H	0.67	1.16	0.18				
**SNGBDI**	**gradients**	2.26	2.28	0.04	0.29	-0.39		2
SNGBDI	richness_L	-0.08	4.93	-0.13				
SNGBDI	richness_M	-1.02	2.17	-0.17				
SNGBDI	richness_H	4.42	-1.27	1.05				
SNGBDI	NDVI_L	0.02	1.76	-1.06				
SNGBDI	NDVI_M	0.93	-0.20	-2.53				
SNGBDI	NDVI_H	3.82	2.53	1.97				
**SRVI**	**gradients**	-0.30	2.36	-0.24	0.92	1.09	0.90	
SRVI	richness_L	2.95	3.46	-0.50				
SRVI	richness_M	1.68	-0.79	0.83				
SRVI	richness_H	0.81	0.78	1.16				

SRVI	NDVI_L	-2.01	2.21	-1.92				
SRVI	NDVI_M	2.37	0.94	0.96				
SRVI	NDVI_H	-2.00	-1.82	-1.59				
**SSAVI**	**gradients**	2.10	3.21	0.48	0.17	1.13	-1.83	
SSAVI	richness_L	3.15	-1.17	-1.58				
SSAVI	richness_M	0.64	1.49	0.16				
SSAVI	richness_H	1.45	1.36	0.64				
SSAVI	NDVI_L	0.26	0.89	0.15				
SSAVI	NDVI_M	0.41	0.89	1.30				
SSAVI	NDVI_H	-0.82	4.03	-0.40				
**TVI**	**gradients**	3.74	1.19	0.66	1.38	0.53	-0.73	2
TVI	richness_L	1.64	1.20	0.00				
TVI	richness_M	-1.06	1.75	-0.05				
TVI	richness_H	2.79	2.01	1.05				
TVI	NDVI_L	1.29	-0.20	-0.54				
TVI	NDVI_M	2.78	-0.24	2.45				
TVI	NDVI_H	-0.51	-0.25	-0.47				
**TVIca**	**gradients**	1.73	3.05	1.12	-0.09	-0.66	-1.78	3
TVIca	richness_L	4.77	-1.14	-1.74				
TVIca	richness_M	2.01	2.19	0.82				
TVIca	richness_H	0.55	1.83	-1.53				
TVIca	NDVI_L	0.95	4.85	3.79				
TVIca	NDVI_M	1.15	0.46	1.52				
TVIca	NDVI_H	-0.86	2.02	1.43				
**TVImn**	**gradients**	2.52	-1.36	0.46	-0.24	1.00	1.03	
TVImn	richness_L	-1.19	-0.39	1.67				
TVImn	richness_M	0.92	0.08	1.26				
TVImn	richness_H	0.95	2.04	0.51				
TVImn	NDVI_L	1.35	0.55	2.15				
TVImn	NDVI_M	1.81	0.42	-0.81				
TVImn	NDVI_H	-0.10	0.79	0.49				

**TVIsd**	**gradients**	3.35	1.51	2.59	-1.57	0.83	1.63
TVIsd	richness_L	3.27	-0.41	1.53			
TVIsd	richness_M	0.83	-1.49	0.61			
TVIsd	richness_H	1.58	1.87	1.11			
TVIsd	NDVI_L	-0.17	1.32	0.85			
TVIsd	NDVI_M	-0.01	0.47	-1.85			
TVIsd	NDVI_H	1.95	-0.43	2.32			

Table S2 The importance values of each spectral index in the random forest model based on Landsat data at different vegetation types and gradient levels (the last column shows the number of times that the top five important indexes are selected repeatedly in all models)

Landsat		**Coniferous forest**	**DBF**	**C&B mixed forest**	**EBF**	**grassland**	**shrubs**	frequency
**Band_1**	**gradients**	2.62	3.07	2.24	0.25	5.58	0.47	2
Band_1	richness_L	0.45	-0.63	-1.75				
Band_1	richness_M	2.14	1.69	-0.01				
Band_1	richness_H	4.48	2.47	0.99				
Band_1	NDVI_L	-3.26	0.11	1.74				
Band_1	NDVI_M	-2.14	-1.02	-0.57				
Band_1	NDVI_H	-0.43	2.69	2.27				
**Band_2**	**gradients**	1.43	2.26	3.87	3.05	6.28	1.68	3
Band_2	richness_L	4.07	-1.21	-0.82				
Band_2	richness_M	0.33	1.59	-1.59				
Band_2	richness_H	0.32	1.24	2.36				
Band_2	NDVI_L	-0.66	0.24	0.44				
Band_2	NDVI_M	1.03	-1.41	-0.17				
Band_2	NDVI_H	-0.06	3.04	1.96				
**Band_3**	**gradients**	1.90	0.88	1.79	1.28	8.23	1.88	3
Band_3	richness_L	-0.25	-0.06	-2.32				
Band_3	richness_M	-1.94	-2.78	1.43				
Band_3	richness_H	2.73	2.49	3.92				
Band_3	NDVI_L	-0.83	2.27	-1.15				
Band_3	NDVI_M	-1.02	0.89	2.85				
Band_3	NDVI_H	3.64	0.88	1.51				
**Band_4**	**gradients**	9.77	0.99	1.42	4.44	3.56	-0.49	5
Band_4	richness_L	2.66	5.12	1.89				
Band_4	richness_M	-1.35	-0.03	-1.40				
Band_4	richness_H	3.38	1.61	1.07				
Band_4	NDVI_L	-2.63	0.12	-1.34				
Band_4	NDVI_M	1.78	0.90	2.13				
Band_4	NDVI_H	4.82	-0.93	2.55				

**Band_5**	**gradients**	1.45	2.27	3.39	-0.77	-1.19	-0.16	3
Band_5	richness_L	3.52	0.71	-1.77				
Band_5	richness_M	-0.57	1.74	1.54				
Band_5	richness_H	0.50	3.64	3.81				
Band_5	NDVI_L	0.21	0.81	3.41				
Band_5	NDVI_M	-0.28	0.64	1.19				
Band_5	NDVI_H	0.94	1.37	2.74				
**band1ca**	**gradients**	3.65	1.09	2.26	4.14	2.02	0.80	
band1ca	richness_L	-1.24	2.01	-0.65				
band1ca	richness_M	-1.06	1.28	1.87				
band1ca	richness_H	-0.88	-0.34	1.96				
band1ca	NDVI_L	-2.40	-0.05	-0.81				
band1ca	NDVI_M	2.68	2.87	-1.99				
band1ca	NDVI_H	-0.66	1.64	1.16				
**band1mn**	**gradients**	0.68	4.42	3.10	3.90	1.43	-1.67	
band1mn	richness_L	-2.10	-1.49	0.12				
band1mn	richness_M	0.78	0.47	-1.05				
band1mn	richness_H	1.21	1.50	2.78				
band1mn	NDVI_L	0.31	2.43	1.12				
band1mn	NDVI_M	0.50	-0.68	-1.74				
band1mn	NDVI_H	1.80	2.69	1.31				
**band1sd**	**gradients**	-1.27	0.97	0.30	2.72	1.79	1.31	
band1sd	richness_L	0.69	1.38	-0.44				
band1sd	richness_M	-2.11	2.29	2.40				
band1sd	richness_H	1.02	-1.18	1.30				
band1sd	NDVI_L	1.69	0.73	-1.16				
band1sd	NDVI_M	2.42	2.32	2.69				
band1sd	NDVI_H	-0.75	1.65	1.56				
**band2ca**	**gradients**	-0.08	1.84	2.53	0.18	2.37	-1.18	
band2ca	richness_L	-0.32	1.15	-1.73				
band2ca	richness_M	0.77	2.94	1.67				

band2ca	richness_H	0.57	0.33	2.02				
band2ca	NDVI_L	-0.24	1.02	-0.61				
band2ca	NDVI_M	1.57	0.80	0.54				
band2ca	NDVI_H	2.07	0.13	3.24				
**band2mn**	**gradients**	0.75	6.05	3.31	4.14	1.99	-0.72	3
band2mn	richness_L	-0.81	-0.90	-1.26				
band2mn	richness_M	1.10	2.88	2.46				
band2mn	richness_H	0.93	1.66	0.83				
band2mn	NDVI_L	0.11	4.39	-1.51				
band2mn	NDVI_M	0.21	2.74	-1.12				
band2mn	NDVI_H	2.28	3.03	1.49				
**band2sd**	**gradients**	1.69	0.18	0.53	1.04	0.74	-0.17	
band2sd	richness_L	-1.65	-1.55	-0.61				
band2sd	richness_M	1.36	3.68	0.00				
band2sd	richness_H	0.73	-0.52	-0.78				
band2sd	NDVI_L	-0.38	2.80	-1.20				
band2sd	NDVI_M	-0.04	2.33	0.98				
band2sd	NDVI_H	-0.13	3.92	1.32				
**band3ca**	**gradients**	1.75	1.30	2.20	1.06	0.79	-1.95	2
band3ca	richness_L	2.17	1.88	0.08				
band3ca	richness_M	0.65	4.55	3.18				
band3ca	richness_H	-0.38	2.36	1.36				
band3ca	NDVI_L	-0.37	2.01	2.11				
band3ca	NDVI_M	0.38	0.15	1.34				
band3ca	NDVI_H	0.31	6.05	2.53				
**band3mn**	**gradients**	1.38	7.25	2.76	6.13	1.81	0.50	4
band3mn	richness_L	4.56	-0.46	-0.38				
band3mn	richness_M	-2.02	1.79	1.58				
band3mn	richness_H	1.28	1.24	3.18				
band3mn	NDVI_L	2.77	2.39	-1.12				
band3mn	NDVI_M	0.65	6.92	1.56				

band3mn	NDVI_H	-1.29	1.79	2.02				
**band3sd**	**gradients**	4.17	1.62	0.81	0.02	-0.12	-2.23	
band3sd	richness_L	1.05	0.59	0.28				
band3sd	richness_M	0.05	2.80	1.88				
band3sd	richness_H	-0.91	-0.27	0.35				
band3sd	NDVI_L	0.22	2.83	1.35				
band3sd	NDVI_M	2.04	1.07	0.98				
band3sd	NDVI_H	-1.71	6.69	1.49				
**band4ca**	**gradients**	1.43	-0.23	2.90	3.08	-0.11	-1.02	
band4ca	richness_L	1.25	-0.03	0.64				
band4ca	richness_M	4.24	2.88	2.08				
band4ca	richness_H	0.88	0.53	0.70				
band4ca	NDVI_L	-2.55	3.37	0.99				
band4ca	NDVI_M	4.68	2.22	1.21				
band4ca	NDVI_H	1.82	4.94	2.60				
**band4mn**	**gradients**	-0.26	7.12	2.64	5.27	2.19	2.05	
band4mn	richness_L	1.90	2.05	-2.14				
band4mn	richness_M	1.31	-0.93	-0.01				
band4mn	richness_H	1.54	0.44	2.60				
band4mn	NDVI_L	1.72	3.44	1.61				
band4mn	NDVI_M	0.72	2.79	-1.53				
band4mn	NDVI_H	1.32	3.95	2.18				
**band4sd**	**gradients**	1.45	-0.02	0.04	1.92	0.65	-1.31	3
band4sd	richness_L	2.50	1.09	-0.01				
band4sd	richness_M	1.68	4.54	1.87				
band4sd	richness_H	0.38	0.96	-0.24				
band4sd	NDVI_L	1.11	3.46	-0.16				
band4sd	NDVI_M	0.29	0.79	0.17				
band4sd	NDVI_H	3.04	5.40	2.20				
**band5ca**	**gradients**	1.42	3.36	2.57	1.06	-1.76	-2.08	
band5ca	richness_L	-1.48	0.08	0.05				

band5ca	richness_M	-1.03	0.76	1.83				
band5ca	richness_H	-2.38	0.92	0.94				
band5ca	NDVI_L	0.82	4.36	-0.77				
band5ca	NDVI_M	-1.74	0.64	0.25				
band5ca	NDVI_H	1.58	2.42	2.58				
**band5mn**	**gradients**	5.93	4.91	3.24	2.16	-0.47	1.46	3
band5mn	richness_L	1.48	1.54	-0.52				
band5mn	richness_M	2.80	1.39	0.24				
band5mn	richness_H	-3.09	0.97	3.98				
band5mn	NDVI_L	1.09	2.49	1.53				
band5mn	NDVI_M	4.80	3.08	-1.58				
band5mn	NDVI_H	-1.36	3.84	1.20				
**band5sd**	**gradients**	0.23	2.66	2.39	2.64	0.70	0.33	
band5sd	richness_L	-1.75	1.34	0.18				
band5sd	richness_M	1.24	1.68	0.43				
band5sd	richness_H	-1.12	0.93	-0.60				
band5sd	NDVI_L	0.41	2.88	-1.56				
band5sd	NDVI_M	-0.10	-0.10	2.26				
band5sd	NDVI_H	-0.60	3.27	3.15				
**ca_zong**	**gradients**	-0.68	0.44	3.28	3.61	0.91	1.59	
ca_zong	richness_L	-1.10	1.58	-0.48				
ca_zong	richness_M	-0.36	2.72	-0.57				
ca_zong	richness_H	2.84	0.84	0.45				
ca_zong	NDVI_L	-1.40	0.35	0.20				
ca_zong	NDVI_M	2.08	0.55	0.70				
ca_zong	NDVI_H	1.37	1.33	2.52				
**DVI**	**gradients**	2.66	3.31	4.05	1.74	5.03	2.46	2
DVI	richness_L	4.10	1.64	0.42				
DVI	richness_M	-0.70	-1.66	1.76				
DVI	richness_H	-0.64	3.21	3.70				
DVI	NDVI_L	-0.01	-0.03	2.08				

DVI	NDVI_M	0.10	2.12	1.22				
DVI	NDVI_H	1.10	0.67	1.78				
**DVIca**	**gradients**	2.58	2.92	0.51	0.12	-1.23	1.48	
DVIca	richness_L	0.79	-1.29	1.09				
DVIca	richness_M	-1.51	1.38	-0.20				
DVIca	richness_H	0.68	1.79	0.19				
DVIca	NDVI_L	0.94	2.37	-0.28				
DVIca	NDVI_M	0.96	-0.44	0.19				
DVIca	NDVI_H	-1.51	3.93	2.38				
**DVImn**	**gradients**	5.55	7.46	3.65	0.88	0.58	3.49	2
DVImn	richness_L	0.05	0.06	0.83				
DVImn	richness_M	0.64	1.97	2.28				
DVImn	richness_H	-1.24	-0.33	3.38				
DVImn	NDVI_L	0.45	3.21	0.43				
DVImn	NDVI_M	3.12	4.30	0.18				
DVImn	NDVI_H	-1.95	3.69	1.42				
**DVIsd**	**gradients**	3.01	2.46	1.03	1.91	2.52	2.36	
DVIsd	richness_L	-1.97	1.78	0.71				
DVIsd	richness_M	1.15	0.22	-0.55				
DVIsd	richness_H	-2.32	1.71	-0.25				
DVIsd	NDVI_L	0.37	2.03	-1.45				
DVIsd	NDVI_M	-0.88	1.13	1.02				
DVIsd	NDVI_H	-0.87	1.01	-0.06				
**EVI**	**gradients**	1.45	2.11	5.22	0.99	6.51	0.68	5
EVI	richness_L	1.50	0.15	-0.67				
EVI	richness_M	-0.45	-1.14	0.12				
EVI	richness_H	-0.38	5.73	3.49				
EVI	NDVI_L	-1.37	-0.65	4.10				
EVI	NDVI_M	-0.88	-1.08	-1.06				
EVI	NDVI_H	0.80	1.41	3.39				
**EVIca**	**gradients**	2.39	1.80	1.91	0.21	0.53	-0.91	

EVIca	richness_L	0.39	0.67	3.19				
EVIca	richness_M	-2.34	2.39	-0.02				
EVIca	richness_H	-0.12	-0.03	1.83				
EVIca	NDVI_L	2.26	-0.53	0.85				
EVIca	NDVI_M	0.06	-0.05	0.44				
EVIca	NDVI_H	0.23	3.81	2.74				
**EVImn**	**gradients**	5.41	7.46	3.39	0.22	0.98	-0.21	3
EVImn	richness_L	-0.51	1.50	1.69				
EVImn	richness_M	1.87	3.67	0.36				
EVImn	richness_H	-2.68	1.04	3.59				
EVImn	NDVI_L	-0.34	2.35	-1.43				
EVImn	NDVI_M	1.69	4.88	0.72				
EVImn	NDVI_H	-2.39	3.53	1.92				
**EVIsd**	**gradients**	0.51	1.20	-0.09	1.54	0.92	0.36	
EVIsd	richness_L	0.78	-0.05	0.93				
EVIsd	richness_M	1.67	1.20	-0.71				
EVIsd	richness_H	0.97	0.77	-0.14				
EVIsd	NDVI_L	-0.48	2.47	-1.06				
EVIsd	NDVI_M	1.76	-0.01	1.43				
EVIsd	NDVI_H	-0.35	-0.68	3.16				
**GCVI**	**gradients**	9.45	4.73	3.04	2.71	5.67	1.02	3
GCVI	richness_L	-1.85	1.63	0.33				
GCVI	richness_M	-2.37	0.81	-0.98				
GCVI	richness_H	1.56	3.77	1.98				
GCVI	NDVI_L	-1.41	2.66	0.97				
GCVI	NDVI_M	4.70	1.66	-0.90				
GCVI	NDVI_H	0.77	0.67	1.11				
**GCVIca**	**gradients**	3.01	2.61	1.60	2.71	2.49	2.01	
GCVIca	richness_L	1.13	1.06	-0.93				
GCVIca	richness_M	-0.51	2.13	0.73				
GCVIca	richness_H	1.26	1.48	1.38				

GCVIca	NDVI_L	1.73	1.62	-0.65				
GCVIca	NDVI_M	1.22	2.82	1.68				
GCVIca	NDVI_H	0.31	1.70	2.69				
**GCVImn**	**gradients**	10.44	6.10	0.80	1.76	0.00	2.01	4
GCVImn	richness_L	-0.17	3.02	0.18				
GCVImn	richness_M	-0.55	1.16	0.31				
GCVImn	richness_H	-0.72	-0.10	1.20				
GCVImn	NDVI_L	0.38	6.06	-1.97				
GCVImn	NDVI_M	5.82	2.24	-0.93				
GCVImn	NDVI_H	0.43	3.49	1.13				
**GCVIsd**	**gradients**	3.12	0.23	3.77	-0.13	2.47	0.34	
GCVIsd	richness_L	2.03	1.54	-1.90				
GCVIsd	richness_M	-0.06	2.34	1.42				
GCVIsd	richness_H	2.91	0.53	1.72				
GCVIsd	NDVI_L	0.23	-1.45	-1.63				
GCVIsd	NDVI_M	2.81	1.33	2.27				
GCVIsd	NDVI_H	1.25	3.11	2.35				
**GNDVI**	**gradients**	8.10	3.58	2.20	2.20	4.58	-1.31	
GNDVI	richness_L	-2.11	1.42	0.67				
GNDVI	richness_M	-0.03	0.98	-0.33				
GNDVI	richness_H	2.75	2.19	-1.82				
GNDVI	NDVI_L	-1.33	2.55	-0.35				
GNDVI	NDVI_M	3.14	1.33	1.56				
GNDVI	NDVI_H	-0.26	-1.75	1.27				
**GNDVIca**	**gradients**	-0.48	1.58	1.03	3.37	0.08	0.96	2
GNDVIca	richness_L	-1.39	1.56	-1.56				
GNDVIca	richness_M	-1.94	2.80	3.68				
GNDVIca	richness_H	1.90	1.41	-1.20				
GNDVIca	NDVI_L	-1.36	1.45	0.99				
GNDVIca	NDVI_M	-0.28	2.92	2.77				
GNDVIca	NDVI_H	0.71	0.86	2.31				

**GNDVImn**	**gradients**	5.12	5.98	-0.55	1.77	1.62	0.77	2
GNDVImn	richness_L	0.62	3.61	1.10				
GNDVImn	richness_M	-0.53	0.65	0.46				
GNDVImn	richness_H	-0.90	0.34	0.52				
GNDVImn	NDVI_L	0.60	4.50	-1.77				
GNDVImn	NDVI_M	3.30	1.85	-0.49				
GNDVImn	NDVI_H	0.55	2.72	2.27				
**GNDVIsd**	**gradients**	1.79	2.36	1.26	1.61	0.11	0.79	
GNDVIsd	richness_L	-1.10	0.63	-0.69				
GNDVIsd	richness_M	-1.48	2.69	1.17				
GNDVIsd	richness_H	-0.46	-1.14	1.20				
GNDVIsd	NDVI_L	-0.05	0.31	0.23				
GNDVIsd	NDVI_M	-1.47	2.12	2.10				
GNDVIsd	NDVI_H	-1.00	0.52	1.97				
**MTVI**	**gradients**	0.14	2.62	4.73	1.85	6.70	1.25	3
MTVI	richness_L	1.47	0.02	1.81				
MTVI	richness_M	-1.25	1.38	0.39				
MTVI	richness_H	-1.33	3.46	4.33				
MTVI	NDVI_L	-1.11	-0.04	2.44				
MTVI	NDVI_M	-0.42	2.43	0.95				
MTVI	NDVI_H	0.91	-0.04	1.39				
**MTVIca**	**gradients**	2.68	2.43	1.28	0.85	1.41	-1.47	2
MTVIca	richness_L	-0.85	0.88	2.24				
MTVIca	richness_M	2.14	-0.73	-0.05				
MTVIca	richness_H	0.03	1.39	0.79				
MTVIca	NDVI_L	3.02	2.65	-1.34				
MTVIca	NDVI_M	0.03	-0.48	0.25				
MTVIca	NDVI_H	-0.62	2.67	2.02				
**MTVImn**	**gradients**	8.78	5.61	2.44	1.00	-0.39	0.47	2
MTVImn	richness_L	0.28	0.99	1.47				
MTVImn	richness_M	1.01	-0.16	1.14				

MTVImn	richness_H	-0.91	2.05	3.12				
MTVImn	NDVI_L	0.18	3.15	-0.61				
MTVImn	NDVI_M	1.62	5.18	-1.94				
MTVImn	NDVI_H	-0.45	3.57	1.23				
**MTVIsd**	**gradients**	1.33	1.15	2.60	1.86	-1.06	2.11	
MTVIsd	richness_L	0.43	-0.51	2.36				
MTVIsd	richness_M	1.67	2.00	-0.04				
MTVIsd	richness_H	-1.37	1.40	-0.50				
MTVIsd	NDVI_L	-0.84	1.34	-1.79				
MTVIsd	NDVI_M	0.77	-0.40	1.30				
MTVIsd	NDVI_H	-1.21	4.18	2.10				
**NDGI**	**gradients**	0.85	2.15	0.93	3.08	4.26	-1.22	2
NDGI	richness_L	0.85	0.21	-1.31				
NDGI	richness_M	-2.46	-0.59	-2.29				
NDGI	richness_H	3.24	4.65	-0.22				
NDGI	NDVI_L	-2.60	0.52	0.91				
NDGI	NDVI_M	2.80	0.53	-1.15				
NDGI	NDVI_H	-0.26	1.81	1.44				
**NDGIca**	**gradients**	1.66	1.28	1.37	1.64	1.02	-0.19	
NDGIca	richness_L	4.22	1.36	1.17				
NDGIca	richness_M	1.31	1.55	-0.03				
NDGIca	richness_H	-0.27	-0.90	1.34				
NDGIca	NDVI_L	-0.41	2.58	2.28				
NDGIca	NDVI_M	-0.25	1.27	-0.49				
NDGIca	NDVI_H	-2.32	1.47	2.30				
**NDGImn**	**gradients**	2.12	2.72	0.65	3.99	1.33	2.50	3
NDGImn	richness_L	0.80	2.24	0.34				
NDGImn	richness_M	-0.63	1.22	4.14				
NDGImn	richness_H	-0.77	-0.62	4.07				
NDGImn	NDVI_L	-0.28	3.18	-2.11				
NDGImn	NDVI_M	1.20	3.52	0.32				

NDGImn	NDVI_H	-1.21	4.54	1.68				
**NDGIsd**	**gradients**	-1.84	1.61	0.96	2.63	1.97	1.14	
NDGIsd	richness_L	2.85	1.44	-0.62				
NDGIsd	richness_M	-0.05	1.71	0.74				
NDGIsd	richness_H	1.87	-0.30	-0.69				
NDGIsd	NDVI_L	0.55	2.21	0.72				
NDGIsd	NDVI_M	0.86	0.72	-0.64				
NDGIsd	NDVI_H	0.51	1.97	1.44				
**NDVI**	**gradients**	3.83	2.36	0.42	0.48	3.84	-0.83	4
NDVI	richness_L	0.33	2.06	1.54				
NDVI	richness_M	2.21	3.80	-0.05				
NDVI	richness_H	-1.08	3.07	0.32				
NDVI	NDVI_L	-0.56	0.84	0.87				
NDVI	NDVI_M	-0.28	-1.33	2.42				
NDVI	NDVI_H	1.13	-1.33	1.35				
**NDVIca**	**gradients**	-0.34	1.42	1.23	-0.71	1.93	0.91	
NDVIca	richness_L	1.60	1.63	-0.38				
NDVIca	richness_M	-0.68	1.68	0.29				
NDVIca	richness_H	1.64	0.37	1.04				
NDVIca	NDVI_L	1.43	3.84	-0.85				
NDVIca	NDVI_M	0.01	0.55	1.45				
NDVIca	NDVI_H	1.14	1.13	1.84				
**NDVImn**	**gradients**	0.41	7.25	-1.12	2.75	1.54	1.68	
NDVImn	richness_L	3.24	1.74	0.58				
NDVImn	richness_M	-0.57	-0.03	0.67				
NDVImn	richness_H	-2.07	1.15	-1.39				
NDVImn	NDVI_L	0.19	6.20	-0.93				
NDVImn	NDVI_M	1.97	4.84	-0.38				
NDVImn	NDVI_H	0.03	1.98	-0.38				
**NDVIsd**	**gradients**	0.22	1.01	1.30	1.29	1.81	0.79	
NDVIsd	richness_L	1.38	1.91	0.81				

NDVIsd	richness_M	-1.41	2.00	1.78				
NDVIsd	richness_H	1.14	-0.66	-1.17				
NDVIsd	NDVI_L	-0.32	3.13	-1.40				
NDVIsd	NDVI_M	-1.96	-0.17	2.10				
NDVIsd	NDVI_H	1.90	1.87	1.81				
**NGBDI**	**gradients**	0.11	3.89	3.46	1.43	-0.40	4.18	
NGBDI	richness_L	2.22	0.62	-1.04				
NGBDI	richness_M	1.99	1.97	1.04				
NGBDI	richness_H	-0.79	2.15	1.46				
NGBDI	NDVI_L	-0.77	1.87	1.19				
NGBDI	NDVI_M	-0.20	-1.14	0.96				
NGBDI	NDVI_H	-2.80	2.04	1.21				
**NGBDIca**	**gradients**	1.43	0.54	1.56	1.82	1.10	0.07	2
NGBDIca	richness_L	-0.13	-1.35	1.36				
NGBDIca	richness_M	0.94	1.63	0.81				
NGBDIca	richness_H	0.20	0.77	0.97				
NGBDIca	NDVI_L	1.04	1.74	0.58				
NGBDIca	NDVI_M	4.82	3.63	3.69				
NGBDIca	NDVI_H	-0.12	2.42	1.26				
**NGBDImn**	**gradients**	3.30	2.09	2.82	2.39	0.86	1.17	
NGBDImn	richness_L	-0.36	-1.72	2.09				
NGBDImn	richness_M	-1.39	0.26	0.28				
NGBDImn	richness_H	0.64	2.21	1.70				
NGBDImn	NDVI_L	-0.28	1.62	2.18				
NGBDImn	NDVI_M	-0.06	-0.02	2.05				
NGBDImn	NDVI_H	0.96	8.58	1.96				
**NGBDIsd**	**gradients**	1.50	1.81	1.20	1.76	0.38	4.68	3
NGBDIsd	richness_L	-1.73	-0.63	0.55				
NGBDIsd	richness_M	-0.39	-0.69	-0.34				
NGBDIsd	richness_H	0.42	-0.19	1.27				
NGBDIsd	NDVI_L	2.53	2.38	-0.44				

NGBDIsd	NDVI_M	5.25	4.57	0.75				
NGBDIsd	NDVI_H	-1.36	2.20	2.42				
**NPCI**	**gradients**	-0.68	4.36	0.53	1.93	-0.43	-1.10	2
NPCI	richness_L	0.48	0.04	-1.35				
NPCI	richness_M	-1.08	0.06	3.45				
NPCI	richness_H	0.58	2.25	0.44				
NPCI	NDVI_L	-3.01	2.84	3.02				
NPCI	NDVI_M	1.92	-0.46	-0.61				
NPCI	NDVI_H	1.65	1.37	1.03				
**NPCIca**	**gradients**	-0.81	2.75	-1.10	1.90	2.74	1.49	
NPCIca	richness_L	0.67	1.73	-1.10				
NPCIca	richness_M	0.11	-1.03	0.82				
NPCIca	richness_H	-0.61	-3.25	-1.68				
NPCIca	NDVI_L	-0.45	1.75	-0.32				
NPCIca	NDVI_M	-0.03	1.23	0.23				
NPCIca	NDVI_H	1.19	6.06	2.41				
**NPCImn**	**gradients**	2.11	2.91	2.43	3.96	1.41	0.09	
NPCImn	richness_L	0.07	-0.71	1.57				
NPCImn	richness_M	-0.44	3.32	7.89				
NPCImn	richness_H	-0.53	1.15	2.38				
NPCImn	NDVI_L	-0.59	-0.12	-0.44				
NPCImn	NDVI_M	3.01	1.85	-0.44				
NPCImn	NDVI_H	0.94	3.27	2.12				
**NPCIsd**	**gradients**	1.86	2.38	2.52	3.35	2.42	0.78	
NPCIsd	richness_L	-0.78	0.06	-1.19				
NPCIsd	richness_M	2.80	-1.08	1.66				
NPCIsd	richness_H	0.02	0.09	0.94				
NPCIsd	NDVI_L	1.52	0.67	0.65				
NPCIsd	NDVI_M	0.16	1.89	0.48				
NPCIsd	NDVI_H	1.07	2.21	1.30				
**OSAVI**	**gradients**	2.58	2.46	1.60	1.76	5.93	0.90	

OSAVI	richness_L	1.65	1.80	1.53				
OSAVI	richness_M	-0.55	1.39	0.48				
OSAVI	richness_H	-0.83	3.98	2.03				
OSAVI	NDVI_L	0.88	0.79	0.42				
OSAVI	NDVI_M	0.65	0.93	0.19				
OSAVI	NDVI_H	-1.39	-0.17	1.64				
**OSAVIca**	**gradients**	0.78	2.00	1.55	1.65	-0.77	1.30	
OSAVIca	richness_L	1.78	-1.26	-1.62				
OSAVIca	richness_M	0.98	0.62	0.51				
OSAVIca	richness_H	1.60	1.55	1.20				
OSAVIca	NDVI_L	1.58	-0.05	0.00				
OSAVIca	NDVI_M	-0.98	0.67	-0.12				
OSAVIca	NDVI_H	-0.71	2.60	2.72				
**OSAVImn**	**gradients**	1.61	10.68	3.19	2.21	-0.18	2.83	4
OSAVImn	richness_L	-1.00	0.24	1.20				
OSAVImn	richness_M	0.44	2.38	0.00				
OSAVImn	richness_H	-0.41	0.16	3.83				
OSAVImn	NDVI_L	-0.22	4.21	-0.43				
OSAVImn	NDVI_M	1.23	5.02	-0.97				
OSAVImn	NDVI_H	0.76	4.00	3.30				
**OSAVIsd**	**gradients**	0.48	1.16	2.13	1.72	2.06	0.81	
OSAVIsd	richness_L	-1.73	-0.31	-0.78				
OSAVIsd	richness_M	-1.56	0.26	-0.13				
OSAVIsd	richness_H	0.68	2.69	-0.15				
OSAVIsd	NDVI_L	0.13	1.44	-1.42				
OSAVIsd	NDVI_M	0.13	1.88	0.19				
OSAVIsd	NDVI_H	-1.38	1.58	2.29				
**RVI**	**gradients**	3.26	1.65	-2.70	1.24	4.94	0.80	
RVI	richness_L	0.35	0.75	-1.37				
RVI	richness_M	-1.88	1.68	-1.68				
RVI	richness_H	0.78	2.40	1.58				

RVI	NDVI_L	-0.48	0.42	0.68				
RVI	NDVI_M	0.22	0.22	-0.01				
RVI	NDVI_H	1.27	1.20	-0.74				
**RVIca**	**gradients**	1.25	1.26	-0.01	2.51	1.98	0.64	
RVIca	richness_L	2.31	-0.46	-1.52				
RVIca	richness_M	-1.06	1.57	3.34				
RVIca	richness_H	-1.45	-0.77	-1.11				
RVIca	NDVI_L	0.24	0.52	-0.90				
RVIca	NDVI_M	2.24	1.66	-0.40				
RVIca	NDVI_H	-0.70	1.24	1.55				
**RVImn**	**gradients**	2.01	12.11	1.86	1.59	1.30	1.80	2
RVImn	richness_L	0.33	1.60	-2.07				
RVImn	richness_M	0.18	1.98	1.34				
RVImn	richness_H	-1.18	0.68	0.48				
RVImn	NDVI_L	0.75	7.35	-1.63				
RVImn	NDVI_M	2.02	2.83	0.83				
RVImn	NDVI_H	-0.67	3.10	-1.32				
**RVIsd**	**gradients**	-1.91	0.68	3.15	-0.51	1.36	-1.46	
RVIsd	richness_L	0.30	-1.12	0.37				
RVIsd	richness_M	-1.66	1.02	1.31				
RVIsd	richness_H	1.13	1.25	0.37				
RVIsd	NDVI_L	0.26	1.26	1.34				
RVIsd	NDVI_M	-2.21	-0.76	2.14				
RVIsd	NDVI_H	2.66	0.54	2.24				
**SAVI**	**gradients**	0.83	3.54	4.32	-0.54	5.72	0.38	3
SAVI	richness_L	1.39	0.12	2.20				
SAVI	richness_M	-2.24	0.59	-1.31				
SAVI	richness_H	-2.27	2.99	3.34				
SAVI	NDVI_L	-0.63	-0.89	2.59				
SAVI	NDVI_M	0.45	0.30	1.70				
SAVI	NDVI_H	-1.13	0.72	2.06				

**SAVIca**	**gradients**	3.70	2.40	2.18	-0.82	1.97	1.97	
SAVIca	richness_L	2.10	1.47	-0.53				
SAVIca	richness_M	-0.40	2.79	1.56				
SAVIca	richness_H	0.19	2.21	1.93				
SAVIca	NDVI_L	2.64	-0.92	1.50				
SAVIca	NDVI_M	-2.74	0.94	-0.27				
SAVIca	NDVI_H	-0.12	5.10	2.00				
**SAVImn**	**gradients**	4.07	8.35	2.88	2.63	0.04	2.08	2
SAVImn	richness_L	0.45	1.46	0.70				
SAVImn	richness_M	1.87	3.20	0.95				
SAVImn	richness_H	-2.67	-1.41	3.64				
SAVImn	NDVI_L	-0.27	3.33	-2.16				
SAVImn	NDVI_M	1.69	5.19	-1.83				
SAVImn	NDVI_H	-2.23	4.05	1.27				
**SAVIsd**	**gradients**	0.24	0.91	0.41	1.84	-0.53	1.99	
SAVIsd	richness_L	-0.65	1.34	1.38				
SAVIsd	richness_M	-0.32	2.32	0.64				
SAVIsd	richness_H	0.90	0.55	2.74				
SAVIsd	NDVI_L	-1.95	3.75	0.99				
SAVIsd	NDVI_M	1.05	1.74	0.10				
SAVIsd	NDVI_H	0.15	0.75	2.40				
**TVI**	**gradients**	2.14	1.41	3.30	0.07	7.10	2.77	3
TVI	richness_L	3.25	0.56	-0.40				
TVI	richness_M	-1.94	0.61	2.06				
TVI	richness_H	1.48	3.17	4.25				
TVI	NDVI_L	1.55	0.03	3.48				
TVI	NDVI_M	0.54	2.07	0.04				
TVI	NDVI_H	-0.04	2.23	2.47				
**TVIca**	**gradients**	2.82	2.20	0.51	1.53	1.82	-1.55	
TVIca	richness_L	0.69	0.49	-0.95				
TVIca	richness_M	1.10	2.54	-0.39				

TVIca	richness_H	-2.16	0.47	-0.13				
TVIca	NDVI_L	3.14	3.05	0.00				
TVIca	NDVI_M	0.29	-0.51	0.64				
TVIca	NDVI_H	-0.46	3.22	1.96				
**TVImn**	**gradients**	6.63	5.87	2.22	-0.40	0.73	2.21	
TVImn	richness_L	0.26	-0.36	-1.09				
TVImn	richness_M	1.87	1.94	0.83				
TVImn	richness_H	-1.18	0.93	4.13				
TVImn	NDVI_L	0.25	3.55	-2.25				
TVImn	NDVI_M	3.37	4.06	-1.58				
TVImn	NDVI_H	0.50	3.88	1.88				
**TVIsd**	**gradients**	-0.57	2.05	1.75	0.91	0.04	3.97	2
TVIsd	richness_L	-0.04	0.01	2.27				
TVIsd	richness_M	0.25	-0.15	1.73				
TVIsd	richness_H	0.10	0.05	1.31				
TVIsd	NDVI_L	-0.82	3.22	-0.26				
TVIsd	NDVI_M	1.22	0.32	0.58				
TVIsd	NDVI_H	1.02	3.90	2.05				

Table S3 Significance values of each variable in random forest models constructed based on different seasons in coniferous forest

**Sentinel-2 Variable**	Spring	Summer	Autumn	Winter	**Landsat Variable**	Spring	Summer	Autumn	Winter
band1	2.29	-2.79	3.41	0.38	band1	1.78	-0.77	1.75	-1.3
band1ca	1.84	1.53	0.37	4	band1ca	0.91	1.52	-2.61	-1.46
band1mn	0.19	0.89	0.57	-1.3	band1mn	2.75	-0.35	3.46	1.71
band1sd	-1.59	-0.89	2.68	2.49	band1sd	2.44	1.83	1.75	0.89
band2	2.1	-0.14	1.63	0.87	band2	1.99	0.77	0.13	1.39
band2ca	-0.1	1.15	1.36	3.87	band2ca	1.35	3.11	-0.47	0.33
band2mn	2.69	1.45	3.17	0.5	band2mn	6.83	-1.89	0.68	1.9
band2sd	-0.68	0.5	0.7	1.7	band2sd	3.29	3.12	-1.72	2.51
band3	-0.55	-1.09	3.25	1.12	band3	3.66	1.04	-0.26	3.44
band3ca	1.48	2.28	-0.06	4.64	band3ca	0.01	0.26	-1.4	2.28
band3mn	3.48	-1.05	-0.77	1.72	band3mn	0.99	-1.37	-0.75	0.94
band3sd	-0.11	1.43	-1.51	2.53	band3sd	5.09	0.17	-0.14	0.41
band4	-0.11	-2.15	1.77	-0.03	band4	-0.4	3.69	-0.14	-0.66
band4ca	-0.57	0.4	-2.65	1.9	band4ca	1.35	2.31	-2.44	2.35
band4mn	3.72	1.75	0.26	1.95	band4mn	1.25	3.02	-1.29	0.16
band4sd	-0.03	-0.4	-0.61	1.96	band4sd	3.93	1.45	1.07	1.56
band5	0.06	1.41	1.26	1.88	band5	3.28	1.58	3.98	1.32
band5ca	0.4	-0.47	1.16	2.56	band5ca	0.07	-0.85	3.07	1.59
band5mn	-0.47	-1.26	2.68	-0.22	band5mn	4.05	4.08	6.1	-0.71
band5sd	1.63	-0.45	2.92	0.25	band5sd	2.13	-0.95	1.74	2.47
band6	-0.39	1.19	0.8	-1.12	ca_zong	0.73	1.86	-0.48	-0.31
band6ca	0.34	0.12	0.88	-0.47	DVI	3.55	1.04	1.58	2.75
band6mn	0.32	0.84	2.57	2.78	DVIca	0.84	4.81	1.55	-0.06
band6sd	-1.48	0.13	4.06	-1.41	DVImn	2.6	1.21	4.06	1.27
band7	0.86	0.37	0.19	1.81	DVIsd	1	1.94	1	-0.73
band7ca	-0.75	-2.25	-1.79	1.64	EVI	0.51	3.84	0.55	-0.25
band7mn	2.32	0.75	2.49	-0.56	EVIca	-1.08	1.41	2.15	5.34
band7sd	-0.39	1.29	-0.54	0.8	EVImn	0.27	5.47	0.98	-1.04
ca_zong	-1.03	-0.89	0.93	0.42	EVIsd	-1.19	-0.61	1.29	6.72
DVI	-1.13	0.69	-0.1	-0.11	GCVI	2.02	1.95	2.39	-1.35
DVIca	-0.32	1.08	-0.9	1.26	GCVIca	-1.65	2.09	1.01	1.32

DVImn	-2.49	0.34	1.74	1.53	GCVImn	1.11	-1.49	1.3	0.49
DVIsd	-0.61	0.78	2.13	0.87	GCVIsd	0.12	2.73	-1.87	1.19
EVI	0.21	0.51	1.32	-0.76	GNDVI	2.12	-1.22	-0.49	-0.99
EVIca	-0.86	0.7	1.91	0.17	GNDVIca	1.09	0.75	1.18	2.32
EVImn	0.16	5.53	1.58	0.63	GNDVImn	1.61	1.25	1.61	0.38
EVIsd	-0.84	3.31	1.58	2.33	GNDVIsd	-1.14	4.73	-1.17	0.01
GCVI	1.75	2.49	1.88	-0.4	MTVI	2.17	2.35	0.36	4.54
GCVIca	-0.48	2.33	1.76	0.37	MTVIca	1.23	1.44	2.12	0.26
GCVImn	2.75	-0.35	4.16	0.69	MTVImn	1.27	1.46	4.45	0.16
GCVIsd	1.48	2.42	1.78	1.21	MTVIsd	-1.53	1.98	-0.97	2.09
GNDVI	2.94	-0.73	2.25	-1.29	NDGI	3.45	1.61	1.09	3.35
GNDVIca	2.45	0.74	0.99	-0.84	NDGIca	1.54	0.71	2.21	-0.59
GNDVImn	2.95	1.47	3.36	-0.16	NDGImn	2.01	3.48	0.61	-0.78
GNDVIsd	-0.55	-0.69	2.29	0.5	NDGIsd	1.42	-0.53	0.34	1.97
MCARI	0.38	0.1	0.08	0.82	NDVI	-1.2	2.2	6.46	-1.59
MCARIca	0.4	6.65	0.31	-1.25	NDVIca	2.11	0.74	0.22	0.55
MCARImn	-0.76	1.97	-1.13	2.23	NDVImn	1.51	0.32	4.63	3.27
MCARIsd	0.43	2.49	-0.57	1.91	NDVIsd	1.68	1.85	1.54	1.27
MTVI	0.16	2.82	0.52	1.99	NGBDI	-0.8	-0.25	1.89	0.12
MTVIca	1.73	-1.54	-0.06	-0.12	NGBDIca	0.36	1.07	-0.07	-0.34
MTVImn	-0.57	4.05	2.49	-0.71	NGBDImn	1.69	0.17	-1.12	0.44
MTVIsd	-0.26	-1.47	-1.03	1.55	NGBDIsd	1.86	1.91	1.06	-2.86
NDGI	1.08	1.31	-1.58	-0.71	NPCI	6.65	2.79	7.75	0.24
NDGIca	1.79	-1.06	0.08	1	NPCIca	-0.16	2.8	1.36	1.27
NDGImn	-0.6	0.33	-0.19	3.6	NPCImn	1.95	-0.26	3.65	-1.01
NDGIsd	1.42	-0.6	1.38	1.22	NPCIsd	1.04	1.51	2.36	-0.71
NDREI	0.04	0.23	1.13	0.06	OSAVI	-0.1	0.13	1.09	0.16
NDREIca	-0.31	0.13	-0.96	1.65	OSAVIca	-1.37	0.67	0.38	1.51
NDREImn	2.96	1.29	3.5	-0.45	OSAVImn	1.68	3.9	4.65	0.39
NDREIsd	0.04	-0.13	0.38	2.83	OSAVIsd	0.72	-2.06	0.29	7.8
NDVI	2.34	2.67	-1.82	-1.17	RVI	-0.5	3.98	3.39	-0.29
NDVIca	0.54	3.16	-0.88	0.34	RVIca	0.01	0.14	-0.49	-0.1
NDVImn	3.76	0.34	2.54	0.07	RVImn	0.67	0.37	1.58	-1.05
NDVIsd	0.19	3.84	-0.06	2.57	RVIsd	0.68	1.41	0.82	1.54
NGBDI	2.11	0.6	2.31	0.4	SAVI	0	-0.07	1.95	0.18
NGBDIca	1.91	-0.49	0.76	3.62	SAVIca	-1.28	-0.54	1.18	0.45

NGBDImn	3.11	3.32	4.76	0.77	SAVImn	1.06	4.12	2.79	-0.27
NGBDIsd	0.89	3.51	3.67	-0.29	SAVIsd	-0.58	-2.92	-0.38	1.8
NPCI	1.57	6.1	0.34	0.11	TVI	0.92	1.74	-0.08	2.83
NPCIca	1.23	0.26	-0.36	1.3	TVIca	-0.37	-0.96	1.14	0.32
NPCImn	0.56	1.88	10.21	3.37	TVImn	2.28	4.94	3.65	0.81
NPCIsd	-0.19	2.82	3.13	2.22	TVIsd	0.51	0	2.21	0.89
OSAVI	3.36	-1.69	2.19	-0.97					
OSAVIca	-0.39	-1.92	-1.15	3.31					
OSAVImn	2.06	1.57	0.88	1.32					
OSAVIsd	1.64	0	-1.12	-0.68					
RVI	3.71	-1.49	0.66	0.97					
RVIca	1.42	0.17	2.92	-0.76					
RVImn	2.89	3.04	2.28	0.54					
RVIsd	1.07	1.99	1.56	-0.66					
SAVI	1.49	0.88	0.16	1.4					
SAVIca	-0.48	1	0.4	2.98					
SAVImn	1.37	1.81	2.13	1.43					
SAVIsd	1.04	0.02	1.23	2.35					
TVI	0.93	1.42	-1.16	-2.37					
TVIca	-0.33	-1.08	0.33	2.18					
TVImn	-1.19	0.58	2	2.11					
TVIsd	0.6	2.66	-0.36	1.43					

Table S4 The VIP values of each variable of the different PLSR models constructed based on the Sentinel-2 data

Variable	Coniferous forestVIP	Coniferous forestRLVIP	Coniferous forestRMVIP	Coniferous forestRHVIP	Coniferous forestNDVILVIP	Coniferous forestNDVIMVIP	Coniferous forestNDVIHVIP	DBFVIP	DBFRLVIP	DBFRMVIP	DBFRHVIP	DBFNDVILVIP	DBFNDVIMVIP	DBFNDVIHVIP	C&B mixed forestVIP	C&B mixed forestRLVIP	RMVIP	RHVIP	NDVILVIP	NDVIMVIP	NDVIHVIP	EBFVIP	grasslandVIP	shrubsVIP
band1	0.91	0.09	0.33	0.96	0.45	0.83	1.26	1.06	0.43	1.16	0.98	1.28	1.97	0.56	1.05	1.22	0.12	1	0.67	1.33	1.17	1.28	0.49	0.51
band1ca	1.13	1.2	0.78	1.15	1.05	0.82	0.7	0.78	0.77	0.09	1.34	0.89	1.08	0.93	0.99	0.56	1.39	1.06	1.02	0.92	1.19	0.92	1.55	0.58
band1mn	0.72	0.58	0.79	0.93	0.91	0.79	1.11	1.18	0.6	1.51	0.99	1.27	2.08	0.54	1	0.88	0.83	0.95	0.82	0.87	1.1	1.12	0.55	1.33
band1sd	1.07	1.21	0.82	1.15	1.03	0.8	0.71	0.83	0.79	0.14	1.35	0.98	1.21	0.89	1.1	0.47	1.4	1.08	0.97	0.91	1.29	1.05	1.58	0.3
band2	0.92	0.81	0.29	1.34	0.86	0.83	1.26	1.05	1.13	1.12	0.57	0.98	1.46	0.76	1.33	0.88	0.33	1.17	0.43	1.47	1.74	1.28	0.3	0.13
band2ca	1.23	1.33	0.15	1.15	1.02	0.78	0.64	0.69	0.91	0.47	1.29	0.69	0.36	0.78	0.95	0.32	1.5	1.06	1.02	0.95	1.16	0.88	1.5	0.04
band2mn	0.68	0.65	0.36	1.03	1.1	0.9	0.97	1.08	0.85	1.34	0.68	1.14	1.65	0.71	1.27	0.75	0.94	1.09	0.88	0.89	1.51	1.18	0.56	0.87
band2sd	1.55	1.31	0.17	1.2	1.02	0.77	1.88	0.74	0.96	0.25	1.34	0.78	0.61	0.85	1.16	0.24	1.54	1.13	1.03	0.91	1.37	1.04	1.52	0.12
band3	0.98	1.36	0.14	1.63	0.87	0.82	1.09	0.84	0.24	0.07	0.92	1.23	1.28	0.62	1.15	0.27	0.66	0.88	0.2	1.09	1.53	1.18	0.6	0.04
band3ca	1.22	1.29	0.57	1.08	1	0.89	0.84	0.78	0.87	0.57	1.11	0.6	0.67	1.17	0.78	0.78	1.46	0.89	1.1	0.89	0.67	0.87	1.61	0.12
band3mn	0.77	1.02	0.77	1.11	0.73	1.03	1	0.92	0.65	0.68	0.95	1.29	1.4	0.67	1.83	0.52	1.21	1.4	0.62	1.07	1.84	1.2	0.46	1.17
band3sd	1.17	1.25	0.45	1.09	0.98	0.81	0.82	0.8	0.87	0.45	1.17	0.62	0.76	1.15	0.9	0.78	1.45	1.14	1.1	0.97	1.01	1.01	1.68	0.63
band4	0.65	0.82	0.7	1.86	1.42	0.74	0.88	1.04	1.69	0.37	0.45	0.87	0.25	0.67	1.59	0.03	0.78	1.24	0.35	1.05	1.86	1.33	0.5	0.21
band4ca	1.18	1.18	1.12	1.52	0.96	0.85	0.67	0.74	1.14	0.21	1.73	0.57	0.71	0.79	0.7	0.05	1.18	0.91	1.07	0.61	0.91	0.83	2	0.42

band4mn	1.27	0.97	0.82	1.41	0.93	1.78	1.14	1.25	0.93	0.42	1.01	0.95	0.15	0.93	1.39	0.07	1.38	1.23	0.73	1.26	1.6	1.17	0.63	0.16
band4sd	1.18	1.21	1.15	1.36	0.96	0.73	0.69	0.75	1.16	0.15	1.67	0.64	0.71	0.89	0.91	0.07	1.31	1.08	1.09	0.64	1.18	1.03	2.05	0.44
band5	0.82	0.39	0.95	0.81	0.96	1.04	1.18	1.07	1.36	1.27	0.5	0.85	1.84	0.86	1.08	1.31	0.15	0.98	0.71	0.8	1.07	1.37	1.04	0.29
band5ca	0.68	0.76	0.34	0.76	0.69	0.83	1.18	0.96	0.5	0.73	0.96	0.86	0.41	0.81	0.8	0.12	0.8	0.87	0.81	1.29	0.97	0.86	0.95	1.05
band5mn	0.81	0.24	1.13	0.84	0.88	1.21	0.91	1.04	1.03	1.36	0.47	0.91	1.23	0.92	1.15	1.69	0.36	0.97	0.84	0.76	1.18	0.92	0.26	1.33
band5sd	0.82	0.69	0.63	0.87	0.67	0.92	1.16	0.96	0.68	0.13	0.96	0.87	0.22	0.79	0.78	0.82	0.89	0.8	0.9	1.31	0.99	0.8	0.94	0.6
band6	0.84	0.35	0.69	0.77	1.33	1.03	1.08	1.12	1.27	1.12	0.52	0.81	1.99	1	1.03	1.33	0.37	0.91	0.82	0.85	1	1.29	0.69	0.26
band6ca	0.81	0.83	1.31	0.78	0.73	0.94	1.09	0.9	0.44	0.85	1.02	0.78	0.34	0.78	0.85	0.01	1.09	0.86	0.9	1.2	0.99	0.89	0.51	0.96
band6mn	0.83	0.21	1.12	0.83	0.69	1.17	0.99	1.04	1	1.35	0.49	0.93	1.38	0.8	1.09	1.71	0.34	0.93	0.85	0.82	1.09	0.84	0.13	1.39
band6sd	0.77	0.75	0.1	0.87	0.69	1.04	1.06	0.89	0.73	0.18	1.01	0.82	0.06	0.84	0.81	0.78	1.1	0.8	0.96	1.22	1.08	0.8	0.5	0.41
band7	0.85	0.5	0.98	1.14	0.62	0.94	1.24	1.03	1.4	1.48	0.7	0.86	1.21	0.99	0.96	1.21	0.36	0.97	0.82	1.22	1	1.22	0.18	0.39
band7ca	0.81	0.86	1.81	0.7	0.79	0.76	1.18	1.03	0.5	1.44	1	0.91	0.53	0.89	1.19	0.07	1.19	1.02	0.93	1.76	0.95	0.89	0.24	1.28
band7mn	0.9	0.28	1.34	0.86	0.76	1.19	0.95	1.04	1.02	1.27	0.47	0.89	0.94	0.93	1.1	1.69	0.48	0.93	0.84	0.75	1.1	0.82	0.25	1.52
band7sd	0.87	0.78	0.2	0.88	0.77	0.84	1.18	1.06	0.71	0.75	1.01	0.89	0.46	0.98	0.89	0.75	1.28	0.83	0.98	1.74	0.91	0.86	0.23	0.65
ca_zong	1.05	1.25	0.04	1	0.97	0.94	0.75	0.78	0.76	0.81	1.18	0.76	0.2	0.88	0.73	0.31	1.33	0.76	0.73	0.95	0.64	0.79	1.43	0.41
DVIca	0.86	0.94	0.59	0.78	0.95	0.71	0.97	0.91	0.61	1.43	0.99	0.8	0.27	0.83	0.9	0.69	1.4	0.87	1.32	1.05	0.54	0.68	1.24	0.34
DVImn	0.9	0.3	1.43	0.88	0.56	1.21	0.83	1.02	0.99	1.18	0.45	0.85	0.71	0.99	1.06	1.76	0.13	0.9	0.83	0.86	0.94	0.82	0.56	1.76

DVIsd	1	0.72	1.33	1.1	0.77	0.88	1.04	0.99	0.84	0.67	1.05	0.84	0.29	0.92	0.92	0.28	1.45	0.79	1.14	1.18	0.84	0.79	1.51	1.31
EVIca	0.91	1.07	0.12	0.87	0.92	0.75	0.86	0.86	0.58	1.42	0.96	0.78	0.2	0.82	0.81	0.82	1.38	0.79	1.45	1.06	0.47	0.68	1.79	0.3
EVImn	0.84	0.75	1.38	0.72	0.54	0.8	0.98	1.23	1.42	1.38	0.48	0.87	0.96	1.61	0.99	1.79	0.04	0.85	0.84	0.95	0.82	0.8	0.52	1.65
EVIsd	1.02	0.98	1.7	0.81	0.8	0.81	1.11	1.01	1.13	0.75	1.1	0.81	0.5	1.34	0.99	0.14	1.44	0.82	1.19	1.12	0.61	0.74	1.82	1.26
GCVI	1.24	0.58	1.36	0.88	1.03	0.98	0.86	0.96	1.11	0.85	0.69	1.47	0.08	0.91	0.96	1.11	1.23	0.81	0.72	0.74	1.11	0.91	0.81	0.52
GCVIca	0.98	1.84	0.31	0.96	1.04	0.91	0.7	0.83	0.54	0.16	0.95	0.93	0.05	0.52	0.92	0.07	0.69	0.88	1.7	0.62	0.36	0.84	0.45	0.59
GCVImn	0.95	0.17	1.89	1.02	1.1	1.02	0.63	1.07	1.04	0.5	0.31	1.09	0.44	1.48	1.1	1.53	0.84	1	0.72	0.68	1.07	0.85	0.75	1.47
GCVIsd	0.8	1.14	1.29	0.85	1.12	0.78	0.76	0.92	0.9	0.06	0.94	0.82	0.1	0.83	0.86	0.59	0.9	0.87	1.45	0.71	0.93	1.18	0.58	0.55
GNDVIca	0.83	1	0.91	0.95	0.9	0.88	0.63	0.88	0.83	1.4	1.06	0.73	0.24	0.84	0.98	0.75	1.33	0.74	1.43	0.89	0.24	0.74	1.1	0.19
GNDVImn	1.01	0.42	1.74	0.93	0.71	1.2	0.66	1.04	0.72	0.81	0.52	0.81	0.42	1.11	0.96	1.76	0.22	0.85	0.7	0.87	0.73	0.88	0.8	1.87
GNDVIsd	0.76	0.96	0.21	0.95	0.95	0.84	0.68	0.9	0.84	1.29	1.15	0.78	0.05	0.88	1.07	0.42	1.35	0.75	1.36	0.93	0.39	0.76	1.5	0.81
MCARIca	1.2	1	0.63	0.92	1.05	0.75	0.68	0.99	0.85	0.41	1.95	0.63	0.63	0.94	0.77	0.66	1.37	0.7	0.76	0.73	0.67	0.84	0.66	2.14
MCARImn	0.86	0.92	1.28	0.59	1.06	0.7	1.05	1.15	1.29	0.24	1.49	0.83	0.66	1.6	1.47	1.49	0.21	1.17	0.78	0.57	1.52	0.99	0.36	2.02
MCARIsd	1.04	0.92	1.28	0.59	1.17	0.7	1.12	1.06	1.1	0.37	1.37	0.78	0.66	1.56	0.94	0.21	1.43	1.07	0.9	0.65	1.02	1.12	0.5	0.89
MTVI	1.28	0.49	1.26	0.84	0.82	0.65	1.36	1.03	0.64	1.57	0.83	1.2	1.53	1.04	1.05	1.4	0.1	0.93	0.95	0.98	1.26	0.94	0.84	0.33

MTVIca	0.82	1.63	1.43	1.02	0.85	1.02	1.02	0.71	1.28	0.57	1.11	0.96	0.13	0.66	0.95	0.22	0.8	0.9	1.58	0.57	0.3	1.12	0.12	0.63
MTVImm	1.11	0.19	1.16	0.78	0.77	0.5	1.29	0.93	1.04	1.52	0.85	1.23	1.69	1.3	1.03	1.42	0.11	0.96	0.96	0.98	1.23	0.87	0.61	1.14
MTVIsd	1.31	1.11	0.75	1.16	0.73	1	1.34	1.04	0.16	0.55	0.82	1.2	0.96	0.56	0.86	1.36	0.24	0.98	0.99	0.99	0.97	1.17	0.44	0.15
NDGIca	0.98	1.25	0.89	0.82	0.96	1.38	0.63	0.99	0.48	1.15	0.86	1.23	0.66	0.86	0.37	0.57	1.41	0.93	0.92	0.9	0.58	1.07	0.17	1.5
NDGImn	1.16	0.54	0.32	0.87	1.23	1.68	1.46	1.26	0.89	1.3	0.75	0.79	1.27	0.99	1.12	1.59	0.49	1.03	0.85	0.97	1.07	0.96	0.32	1.4
NDGIsd	1.07	0.68	1.95	1.04	1.2	0.56	0.75	1	0.97	0.6	0.72	0.78	0.57	1.26	0.77	0.9	1.38	1.14	0.75	1.16	0.72	1	2.05	1.43
NDREI	0.97	0.8	0.87	0.8	1.49	1.23	0.83	0.99	0.79	1.09	0.68	1.03	1.74	0.37	0.89	1.21	1.31	0.87	0.29	0.72	1.26	0.84	0.22	0.45
NDREIca	0.88	1.48	0.71	1.06	0.8	1.4	0.63	0.79	0.63	0.58	0.72	1.02	0.48	0.46	0.76	0.79	0.8	0.91	1.36	0.65	0.16	0.99	0.32	2.26
NDREImn	0.82	0.4	1.16	0.96	0.57	1.31	0.47	0.93	0.87	0.97	0.44	0.87	1.71	0.98	0.96	1.58	0.69	0.79	0.85	0.8	1	1	0.69	1.19
NDREIsd	0.88	1.74	0.74	0.94	0.78	1	0.68	0.8	0.72	0.34	0.98	0.89	0.16	0.53	0.8	0.24	1.09	1.01	1.13	0.71	0.28	1.07	0.53	1.73
NDVIca	0.86	1.14	0.4	0.92	0.86	0.78	0.63	0.88	0.82	1.31	0.92	0.69	0.18	0.78	0.7	0.89	1.33	0.71	1.52	1.02	0.39	0.77	1.82	0.29
NDVImn	0.84	0.47	1.31	0.88	0.52	1.26	0.39	0.95	0.78	0.96	0.62	0.78	0.12	1.01	0.9	1.73	0.39	0.82	0.73	0.94	0.56	0.89	0.55	1.84
NDVIsd	0.92	1.15	0.82	0.98	0.89	0.82	0.72	0.81	0.72	1.19	1.09	0.78	0.08	0.8	0.89	0.71	1.34	0.81	1.36	1	0.42	0.79	1.81	1.1

NGBDIca	1.02	0.94	0.67	1.05	1.1	0.63	1.95	0.82	2.17	0.19	1.75	0.59	0.29	1.38	0.89	0.98	0.67	1.17	0.57	1.22	0.99	1.01	0.62	0.82
NGBDImn	1.17	1.44	1.15	1.16	1.27	1.21	1.29	1.41	0.96	1.49	1.63	1.25	2.28	1.11	1.07	0.91	0.37	1.29	1.3	1.49	1.11	0.94	0.52	0.17
NGBDIsd	1.38	0.84	0.34	1.17	1.21	0.49	1.44	1.51	0.98	1.07	0.26	1.04	2.19	0.5	0.83	0.44	0.71	1.04	1.02	1.05	0.85	1.46	0.57	1.23
NPCI	0.84	1.14	0.28	1.05	0.96	1.43	1.21	1.27	0.5	1.84	0.78	0.88	2.21	0.23	0.75	1.36	0.6	0.98	0.82	1.02	0.39	1.22	0.7	1.17
NPCIca	0.86	0.69	0.83	1.34	1.34	0.72	1.02	0.87	0.93	0.86	0.55	1.57	0.26	0.81	1.26	0.54	0.66	2.43	1	0.9	1.5	2.32	0.68	0.32
NPCImn	0.91	0.69	0.09	0.72	1.38	1.58	1.51	1.34	0.54	1.63	0.83	0.9	2.49	0.49	0.75	1.55	0.27	1.1	0.78	1.12	0.57	1.22	0.52	0.27
NPCIsd	1.15	1.09	0.52	1.2	1.1	1.2	0.8	1.03	0.44	0.39	0.66	1.49	0.34	0.66	0.8	0.77	1.45	1.35	0.59	1	0.86	1.14	0.69	0.38
OSAVI	1.17	0.51	1.55	1.01	1.68	1.24	0.85	1.15	1.28	1.69	0.89	2.18	0.66	1.02	1.1	0.95	1.1	0.93	1.85	1.2	0.95	0.79	0.83	1.25
OSAVIca	2.03	2.04	0.18	0.79	2.01	1.36	0.53	1.15	0.14	0.14	1.21	1.35	0.21	0.63	0.65	0.32	1.35	1.09	1.09	0.81	0.47	0.83	0.26	0.49
OSAVImn	1.46	0.46	2.07	1.1	1.97	1.29	0.61	0.97	1.22	1.38	0.54	1.51	0.96	1.54	1.22	1.11	0.77	0.86	1.77	0.66	0.85	0.93	0.65	0.45
OSAVIsd	0.75	2.16	0.9	0.92	1.09	0.95	0.57	0.9	0.44	0.01	1.01	1.36	0.08	0.56	0.92	0.11	0.96	1.07	1.03	0.68	0.54	1.06	0.45	0.13
RVIca	0.91	1.06	0.79	1.02	0.89	0.82	0.8	0.83	0.73	0.97	1.22	0.79	0.35	0.81	0.8	0.55	1.42	0.85	1.36	1.08	0.43	0.84	1.86	1.28
RVImn	0.88	0.38	1.52	0.95	0.47	1.23	0.46	0.93	0.86	0.84	0.45	0.77	0.04	0.99	0.96	1.71	0.1	0.83	0.77	0.93	0.7	0.87	0.58	1.31
RVIsd	0.98	0.74	1.62	1.15	0.83	0.88	0.85	0.9	0.92	0.66	1.18	0.8	0.48	0.88	0.96	0.03	1.45	0.92	1.23	1.13	0.82	0.86	1.8	1.75
SAVIcc	0.95	1.2	0.08	0.91	0.91	0.85	1.01	0.79	0.62	1.46	0.92	0.76	0.01	0.95	0.83	0.82	1.36	0.75	1.48	1	0.41	0.7	1.82	1.91

SAVImn	0.78	1.03	0.13	0.63	0.53	1.03	1.08	1.13	1.3	1.12	0.51	0.8	0.39	1.68	1	1.76	0.07	0.87	0.8	0.88	0.81	0.83	0.55	0.31
SAVIsd	0.64	0.41	0.24	0.64	0.86	0.59	0.9	1.3	1.22	1.1	1.1	0.82	0.67	1.82	0.98	0.29	1.41	0.78	1.27	1.08	0.5	0.81	1.81	1.8
SDVI	0.9	0.49	0.97	0.84	0.76	0.91	1.02	0.99	1.42	1.17	0.73	1.03	1.33	0.89	0.97	1.2	0.87	0.93	0.44	1.03	0.9	0.88	0.66	1.21
SEVI	0.84	0.72	1.31	0.65	0.85	0.79	0.99	1.2	1.42	1.35	0.71	0.91	1.03	1.56	0.94	1.26	0.82	0.99	0.31	1.15	0.89	0.74	0.54	0.29
SGNDVI	1.2	0.66	1.27	0.91	0.97	1.01	0.73	0.95	1.11	0.75	0.82	1.42	0.19	1.08	0.83	1.06	1.07	0.94	0.76	0.23	1.09	0.83	1.2	0.26
SMCARI	0.83	0.91	1.28	0.59	1.77	0.7	1.06	1.19	1.44	0.87	1.53	0.8	0.66	1.64	1.42	0.49	0.37	1.35	0.72	1.44	1.77	0.66	0.75	0.32
SNDGI	1.23	0.77	0.07	0.77	1.58	1.76	1.91	1.05	1.77	1.64	1.35	0.83	0.31	1.14	1.08	1.12	0.46	1.23	0.4	1.55	1.35	0.83	0.51	0.06
SNDVI	0.89	0.75	0.92	0.86	0.86	1.12	0.61	0.93	1.3	1.01	1.03	1.28	0.33	1.12	0.86	1.1	0.99	1.01	0.53	0.94	1.01	0.83	0.86	0.33
SNGBDI	1.1	1.31	0.72	1.97	0.8	0.75	1.29	1.14	2.06	0.91	1.71	1.29	1.89	0.93	1.02	0.9	0.22	1.1	0.87	0.79	1.25	1.39	0.77	0.32
SRVI	0.94	0.59	1	0.81	0.86	1.11	0.62	0.96	1.29	1.06	0.77	1.29	0.32	1.12	0.92	1.15	1.02	0.85	0.56	0.95	0.93	0.85	0.77	0.41
SSAVI	0.83	1.25	0.37	0.71	0.78	0.66	1.11	1.05	0.99	1.11	0.84	1.12	0.26	1.72	0.92	1.15	0.91	0.98	0.48	1	0.94	0.78	1.55	0.32
TVI	0.87	0.48	1.02	0.81	0.81	0.99	1.03	1	1.42	1.34	0.75	1.01	1.19	0.9	0.99	1.22	0.98	0.88	0.46	1.18	0.92	1.01	0.85	0.37
TVIca	1.15	2.31	0.29	0.94	0.94	1.04	0.57	0.78	0.25	0.04	1.11	1.02	0.58	0.49	1	0.31	0.71	0.9	1.61	0.52	0.12	1.12	0.17	0.87
TVImn	0.82	0.19	1.42	0.8	0.79	1.14	0.79	1.08	1.14	1.03	0.53	0.99	1.42	1.49	1.02	1.61	0.88	0.91	0.82	0.72	0.93	0.86	0.62	1.2
TVIsd	0.77	1.41	1.5	0.88	0.78	0.85	0.73	0.85	0.67	0.48	1.23	0.85	0.03	0.82	1.06	0.55	1.08	0.93	1.25	0.7	0.98	1.16	0.43	0.22

Table S5 The VIP values of each variable of the different PLSR models constructed based on the Landsat data

Variable	ConiferousforestVIP	ConiferousforestRLVIP	ConiferousforestRMVIP	ConiferousforestRHVIP	Coniferous forest NDVILVIP	Coniferous forestNDVIMVIP	Coniferous forest NDVIHVIP	DBFVIP	DBFRLVIP	DBFRMVIP	DBFRHVIP	DBFNDVILVIP	DBFNDVIMVIP	DBFNDVIHVIP	C&B mixed forestVIP	C&B mixed forestRLVIP	RMVIP	RHVIP	NDVILVIP	NDVIMVIP	NDVIHVIP	EBFVIP	grassland VIP	shrubs VIP
Band_1	0.74	0.77	1.48	1.74	0.04	1.6	1.27	0.93	0.5	0.96	1.15	0.56	0	0.96	1.5	1.23	0.62	1.34	1.69	0.37	1.18	0.6	1.78	1.6
Band_2	0.98	1.98	0.81	2.09	0.77	1.12	1.23	0.86	0.6	1.22	1.12	0.09	0.01	0.76	1.43	1.28	0.76	1.36	1.56	0.13	1.22	0.91	0.84	1.05
Band_3	1.39	2.07	0.66	2.34	1.48	1.08	1.2	0.8	1.11	0.32	1.41	0.6	0.65	0.89	1.25	1.01	0.35	1.44	1.27	1.27	1.24	0.98	1.25	0.64
Band_4	0.77	1.46	0.51	2.62	0.35	0.85	0.73	0.81	0.98	0.71	0.47	0.87	0.25	0.92	1.22	1.07	0.44	1.16	0.81	1.62	1.24	1.62	0.41	0.74
Band_5	1	1.58	0.87	1.47	0.86	0.81	0.08	0.82	1.23	0.39	1.5	0.21	0.57	0.89	0.96	0.8	0.37	1.36	1.33	1.09	1.16	0.9	0.6	1.06
band1ca	0.95	0.82	1.59	0.83	2.81	1.06	0.45	0.96	0.32	1.29	0.54	0.19	1.02	0.9	1.26	1.37	0.59	1.24	1.35	0.96	1.05	0.97	1.12	0.38
band1mn	0.95	1.03	0.84	0.39	1.35	1.72	0.68	0.89	1.24	2.21	1.5	0.14	0.32	0.73	1.26	1.17	1.51	1.25	1.43	0.11	1.07	1.26	1.38	0.66
band1sd	0.4	0.53	1.82	0.76	0.62	0.94	0.66	1.03	0.78	1.38	0.17	0	1.02	0.85	0.82	1.26	0.96	0	0.09	0.93	0.83	0.53	1.07	0.11
band2ca	1.02	1.53	1.16	0.44	2.1	1.01	0.88	0.95	0.17	1.95	0.46	0.31	1.03	0.8	1.22	0.9	1.12	1.28	1.5	1.02	1.22	1.16	1.03	0.5
band2mn	0.58	1.9	0.46	0.44	0.95	0.69	0.68	0.77	1.78	2.03	1.56	0.52	0.56	0.83	1.29	1.03	1.61	1.27	1.48	0.37	1.08	1.12	1.46	0.7
band2sd	0.4	0.44	1.77	0.37	0.64	0.92	1.08	1.02	0.46	1.46	0.1	0.21	1.07	0.81	0.8	0.84	1.36	0.06	0.07	0.97	0.87	0.48	0.98	0.1

band3ca	0.92	1.59	1.24	0.06	1.3	0.76	0.93	0.91	0.75	2.36	0.35	1.43	0.84	0.82	1.04	1.08	1.79	1.11	0.95	0.81	1.21	1.25	0.88	0.75
band3mn	0.65	1.95	0.25	0.29	1.42	0.72	1.04	1.03	1.65	0.87	1.8	0.89	0.13	1.23	1.21	0.91	1.15	1.38	1.33	0.14	1.12	1.04	1.5	0.71
band3sd	0.35	0.33	1.7	0.05	0.62	0.91	1.19	0.87	0.8	1.57	0.03	1.06	0.81	0.78	0.79	1.25	1.81	0.5	0.15	0.84	1.01	0.74	0.83	0.33
band4ca	0.98	1.4	1.23	0.48	0.87	0.97	1.38	0.88	0.66	2.08	0.01	1.4	0.95	0.73	0.96	0.86	1.63	1.02	0.77	0.78	1.2	1.15	0.86	0.74
band4mn	1.06	1.44	0.88	0.7	0.51	0.81	1.89	0.87	1.56	0.64	0.87	0.98	0.99	0.87	1.1	0.99	0.64	1.03	1.12	0.53	0.83	0.71	1.49	0.85
band4sd	0.35	0.38	1.51	0.26	0.7	0.89	1.6	0.91	0.75	1.28	0.28	0.98	1.01	0.78	0.82	0.92	1.63	0.55	0.11	0.83	0.97	0.76	0.84	0.23
band5ca	0.66	0.17	1.14	0.54	0.98	0.77	0.36	0.85	1.13	0.25	0.69	1.28	1.11	1.21	0.86	0.94	0.94	0.9	0.3	1.1	1.03	1.3	0.88	0.55
band5mn	1.3	0.32	1.27	0.7	1.21	1.11	0.96	1.3	1	0.3	1.6	0.81	1.43	1.1	0.91	0.85	0.78	1.41	1.09	1.21	1.15	0.78	1.08	0.82
band5sd	0.57	0.13	1.92	0.83	0.57	1.33	0.65	0.81	1	0.28	0.41	1.35	1.03	1.21	0.67	0.98	1.09	0.12	0.45	0.87	0.52	1.47	0.88	0.8
ca_zong	0.96	1.27	0.73	0.46	1.61	1.02	0.93	0.89	0.28	1.85	0.4	0.68	1.07	0.86	0.98	0.77	1.27	0.93	0.87	0.97	1.2	0.88	0.91	0.6
DVI	1.05	1.18	0.89	1.34	0.81	0.81	0.13	0.86	1.29	0.48	1.57	0.43	0.59	0.89	0.93	0.79	0.35	1.32	1.34	1.05	1.15	0.72	0.6	1.01
DVIca	0.76	0.68	0.7	0.53	0.08	0.91	0.82	0.88	0.98	0.22	0.48	1.22	1.16	1.19	0.83	0.92	0.93	0.82	0.15	1.14	0.99	1.14	0.75	1.31
DVImn	1.25	0.06	1.32	0.71	1.2	1.14	0.77	1.31	0.96	0.16	1.51	1.13	1.5	1.08	0.9	0.85	0.73	1.38	1.04	1.22	1.14	0.81	0.86	0.81

DVIsd	0.88	0.48	0.28	0.94	0.12	1.15	1	0.79	0.92	0.21	0.22	1.34	1.08	1.19	0.74	0.97	1.06	0	0.54	0.9	0.41	1.09	0.79	1.32
EVI	1.07	0.79	0.93	1.17	0.51	0.82	0.06	0.87	1.31	0.37	1.76	0.73	0.49	0.89	0.99	0.75	0.15	1.39	1.51	0.87	1.2	0.76	0.53	0.89
EVIca	0.77	0.81	0.77	0.58	0.06	1	0.95	0.92	0.84	0.14	0.35	1.03	1.21	1.11	0.87	0.82	0.81	0.8	0.15	1.22	1.02	1.09	0.89	0.92
EVImn	1.2	0.33	1.25	0.72	1.1	1.14	0.74	1.23	1.04	0.54	1.59	1.35	1.43	0.98	0.89	0.8	1.05	1.44	1.1	1.36	1.17	0.86	0.82	0.88
EVIsd	0.86	0.5	0.09	0.86	0.15	1.19	1.07	0.8	0.82	0.21	0.17	1.18	1.17	1.12	0.73	0.88	0.94	0.12	0.46	1.05	0.54	0.85	0.91	1.1
GCVI	1.6	0.3	0.66	1.08	0.81	1.29	1.19	0.9	1.49	0.99	0.13	1.13	0.14	1.23	0.94	0.95	0.12	1.2	1.57	0.34	1.04	1.07	1.36	1.07
GCVIca	0.91	1.4	0.26	0.62	0.4	0.94	1.13	0.89	0.19	1.09	0.42	0.49	1.12	1.03	1.29	0.88	1.33	1.35	1.54	1.34	1.23	0.98	1.3	0.83
GCVImn	1.55	1.03	1.11	0.79	0.43	1.27	0.66	1.29	1.34	0.82	0.69	2.33	1.24	0.88	1.02	1.15	0.33	0.37	0.68	0.97	0.79	1.64	1.29	0.79
GCVIsd	0.93	0.72	0.4	1.01	0.34	1.08	1.08	0.89	0.62	1.23	0.2	0.13	1.06	1.23	1.15	0.93	1.27	1.1	0.96	1.37	1.19	0.98	1.46	1.28
GNDVI	1.2	0.43	0.7	1.41	0.07	1.13	1.41	0.89	1.2	1.19	0.16	1.11	0.1	1.27	0.9	0.9	0.1	0.44	0.33	0.36	1.1	0.27	1.3	1.24
GNDVIca	0.88	0.32	0.73	0.74	0.48	0.91	1.44	0.94	0.26	0.91	0.52	0.66	1	0.95	0.89	0.9	1.24	0.5	0.27	1.18	1	0.63	1.23	0.86
GNDVImn	1.22	0.34	0.72	0.63	0.07	1.33	0.08	1.04	0.69	0.81	0.67	1.84	1.16	0.91	0.95	1.03	0.43	0.09	0.36	1.06	0.5	1.57	1.22	0.75
GNDVIsd	0.89	1.37	0.49	0.79	0.52	0.9	1.41	0.89	0.23	0.95	0.5	0.7	1.03	0.97	0.9	0.92	1.22	0.6	0.36	1.17	1.03	0.58	1.33	0.96
MTVI	1.05	1.02	0.93	1.36	0.9	0.81	0.06	0.88	1.32	0.49	1.67	0.47	0.64	0.84	0.95	0.79	0.34	1.34	1.39	1.07	1.15	0.68	0.59	0.98

MTVIca	0.76	0.94	0.59	0.57	0.11	0.9	0.9	0.9	0.9	0.28	0.45	1.11	1.18	1.18	0.85	0.92	0.96	0.87	0.13	1.07	1	1.12	0.77	1.44
MTVImn	1.18	0.17	1.35	0.7	1.25	1.09	0.68	1.32	0.98	0.19	1.54	1.1	1.5	1.12	0.92	0.83	0.8	1.4	1.06	1.21	1.14	0.81	0.97	0.83
MTVIsd	0.87	0.6	0.38	0.94	0	1.12	1.06	0.81	0.86	0.26	0.2	1.22	1.09	1.17	0.7	0.99	1.09	0.02	0.58	0.82	0.41	0.99	0.78	1.32
NDGI	1.17	0.7	0.83	1.75	0.57	1.15	0.06	1.19	1.23	0.68	2.48	0.92	0.59	0.95	0.89	1.1	0.14	1.11	1.25	0.03	1.01	1.91	1.06	0.91
NDGIca	0.73	0.78	0.58	0.1	0.23	0.84	1.46	1.29	1.16	0.64	0.29	0.06	1.35	0.87	0.8	0.92	1.43	1.04	0.19	1.09	1.06	0.68	0.75	0.66
NDGImn	1.34	0.89	1.13	1.58	1.2	1.06	1.2	1.3	1.43	0.25	1.13	1.48	1.3	1	1.05	0.81	1.23	1.22	0.44	0.52	0.84	0.77	1.31	1.06
NDGIsd	1.05	0	0.47	0.8	0.37	0.98	1.34	0.84	1.22	0.72	0.24	0.36	1.16	0.9	0.86	0.89	1.5	0.55	0.13	1.06	0.85	0.76	0.78	1.14
NDVI	1.09	0.72	0.69	1.91	0.03	0.8	1.34	0.97	1.18	1.13	1.77	1.16	0.79	0.89	0.63	0.89	0.02	0.31	1.53	0.7	0.24	1.19	0.52	1.17
NDVIca	0.76	1.8	0.84	0.62	0.05	0.95	1.57	0.96	0.21	0.73	0.36	0.77	1.12	0.82	0.84	0.92	1.27	0.75	0.16	0.96	0.97	0.49	0.94	0.62
NDVImn	1.11	1.04	1.04	0.64	0.77	0.94	0.74	1.04	1.04	0.51	0.1	1.52	1.42	0.85	0.83	0.83	0.07	0.61	0.11	1.18	0.43	1.04	1	0.8
NDVIsd	0.79	1.66	0.69	0.69	0.07	0.94	1.55	0.87	0.33	0.76	0.34	0.92	1.14	0.82	0.83	0.93	1.26	0.77	0.21	0.94	0.97	0.48	0.96	0.68
NGBDI	0.9	0.02	0.52	0.87	0.66	0.91	0.96	1.02	0.54	1.21	0.52	0.47	0.32	0.92	1.3	1.24	1.07	0.64	1.63	0.44	0.68	0.53	0.98	2.05
NGBDIca	1.15	0.43	1.5	0.82	2.97	1.23	0.44	1.01	0.89	1.4	0.12	0.93	0.92	0.96	1.25	1.66	0.73	1.31	1.57	1.1	1.19	1.39	0.99	0.72

NGBDImn	0.55	0.15	0.8	0.61	0.89	0.38	0.43	0.99	0.76	2.22	0.48	0.31	0.58	1.32	1.29	1.18	0.88	1.3	1.55	0.54	1.21	1.45	1.53	0.93
NGBDIsd	1.22	0.47	1.39	0.55	3.22	1.2	0.76	1.24	0.74	1.06	0.06	0.78	0.81	1.34	1.27	1.66	0.6	1.32	1.56	1.15	1.19	1.41	0.97	0.88
NPCI	0.98	0.7	1.1	0.36	0.17	0.43	1.12	0.98	1.24	0.7	1.76	0.89	0.09	0.71	1.41	1.14	1.2	0.41	1.63	0.51	0.45	0.96	0.64	1.88
NPCIca	1.3	1.71	0.94	0.43	1.14	0.46	1.03	1.69	0.94	0.35	1.3	1.36	0.06	1	0.3	1.56	1.22	0.18	0.13	1.52	0.07	1.18	1.05	1.24
NPCImn	1.08	0.88	0.86	0.78	1.08	0.74	0.28	0.78	1.11	2.16	1.1	1.18	0.04	0.92	1.25	1.16	1.4	1.3	1.35	0.96	1.21	0.9	1.03	1.31
NPCIsd	1.24	0.58	1.84	0.79	1.36	0.79	1.23	1.22	0.91	0.46	0.26	0.33	1.04	1.12	0.62	1.04	1.06	0.37	0.53	1.3	1.2	0.79	1.06	1.16
OSAVI	1.1	0.29	0.8	1.07	0.18	0.74	0.64	0.94	1.32	0.86	1.77	0.82	0.65	0.85	1.03	0.79	0.27	1.38	1.46	0.85	1.2	1.01	0.51	1.2
OSAVIca	0.8	1.51	0.91	0.8	0.42	0.96	1.27	0.96	0.49	0.33	0.04	0.14	1.16	1.03	1.16	0.93	0.89	1.06	0.69	1.18	1.08	0.89	0.84	0.99
OSAVImn	1.12	0.62	1.07	0.72	0.79	1.1	0.46	1.21	0.85	0.21	1.09	1.56	1.5	1.02	0.99	0.83	0.53	1.36	0.84	1.28	1.14	1.16	0.81	0.81
OSAVIsd	0.82	1.23	0.57	0.89	0.38	0.99	1.28	0.85	0.46	0.28	0.06	0.19	1.2	1.03	1.27	1.02	0.91	0.86	0.46	1.13	0.93	1.52	0.86	1.11
RVI	1.29	0.56	0.82	1.55	0.01	0.79	0.94	0.96	1.6	0.84	1.76	1.24	0.78	0.9	0.92	0.84	0.03	0.16	1.33	0.68	0.22	0.23	0.58	0.98
RVIca	1.08	1.42	0.13	0.65	0.6	0.95	1.55	0.81	0.6	0.78	0.27	0.32	1.16	1.12	0.84	0.88	1.56	0.58	0.22	1.11	0.98	0.44	0.89	0.64
RVImn	1.18	0.98	1.25	1.19	1.23	1.06	0.13	1.46	1.54	0.27	0.42	2.36	1.66	0.79	0.99	1.02	0.42	0.35	0.33	1.15	0.02	0.9	1.05	0.87
RVIsd	1.07	0.59	0.73	1.11	1.14	0.99	1.49	0.9	1.05	0.83	0.19	0.14	0.82	1.27	1.04	0.89	1.57	0.64	0.46	0.92	1.03	0.43	0.91	1.2

SAVI	1.08	0.78	0.89	1.09	0.41	0.75	0.39	0.89	1.32	0.67	1.67	0.57	0.61	0.87	0.99	0.79	0.33	1.39	1.5	0.98	1.19	0.8	0.56	1.15
SAVIca	0.79	1.09	0.77	0.7	0.26	0.9	1.03	0.94	0.78	0.2	0.26	0.76	1.16	1.12	0.88	0.87	0.82	0.95	0.36	1.2	1.07	1.04	0.94	1.17
SAVImn	1.18	0.35	1.2	0.74	0.93	1.13	0.72	1.26	0.9	0.02	1.35	1.35	1.5	1.07	0.86	0.84	0.64	1.4	1.06	1.26	1.15	0.83	0.77	0.82
SAVIsd	0.82	0.76	0.19	0.9	0.27	1.05	1.11	0.82	0.73	0.16	0.17	0.9	1.18	1.12	0.79	0.97	0.88	0.48	0.14	1.08	0.76	0.94	0.97	1.31
TVI	1.05	1.08	0.91	1.35	0.87	0.81	0.09	0.87	1.31	0.49	1.63	0.45	0.62	0.85	0.95	0.79	0.35	1.34	1.37	1.07	1.15	0.69	0.57	0.99
TVIca	0.76	0.85	0.62	0.56	0.04	0.91	0.87	0.89	0.93	0.25	0.47	1.16	1.17	1.18	0.84	0.92	0.95	0.85	0.14	1.1	0.99	1.13	0.76	1.39
TVImn	1.21	0.13	1.34	0.7	1.23	1.11	0.71	1.32	0.97	0.18	1.53	1.11	1.5	1.11	0.91	0.84	0.77	1.39	1.05	1.22	1.14	0.81	0.94	0.82
TVIsd	0.87	0.56	0.35	0.94	0.05	1.13	1.04	0.8	0.88	0.24	0.21	1.27	1.09	1.18	0.71	0.98	1.08	0.01	0.57	0.85	0.41	1.03	0.77	1.33
